# Biarylacetamides: a novel class of late-stage autophagy inhibitors

**DOI:** 10.1080/27694127.2025.2541597

**Published:** 2025-08-07

**Authors:** Mélissa Lallier, Rani Robeyns, Freke Mertens, Angela Sisto, Guido R.Y. De Meyer, Koen Augustyns, Maya Berg, Winnok H. De Vos, Vincent Timmerman, George M.C. Janssen, Peter van Veelen, Alexander L.N. van Nuijs, Nikolai Engedal, Wim Martinet, Pieter Van der Veken

**Affiliations:** a Laboratory of Medicinal Chemistry, Department of Pharmaceutical Sciences, University of Antwerp, Antwerp, Belgium; b Laboratory of Physiopharmacology, Department of Pharmaceutical Sciences, University of Antwerp, Antwerp, Belgium; c Toxicological Centre, Department of Pharmaceutical Sciences, University of Antwerp, Antwerp, Belgium; d Peripheral Neuropathy Research Group, Department of Biomedical Sciences, University of Antwerp, Antwerpen, Belgium; e Infla-Med Centre of Excellence, University of Antwerp, Antwerpen, Belgium; f Antwerp Centre for Advanced Microscopy, University of Antwerp, Antwerp, Belgium; g µNEURO Centre of Excellence, University of Antwerp, Antwerp, Belgium; h Laboratory of Cell Biology and Histology, Department of Veterinary Sciences, University of Antwerp, Antwerp, Belgium; i Center for Proteomics and Metabolomics, Leiden University Medical Center (LUMC), Leiden, Netherlands; j Autophagy in Cancer Lab, Department of Tumor Biology, Oslo University Hospital, Oslo, Norway

**Keywords:** Autophagy inhibitor, biarylacetamide derivatives, proteomics, metabolomics, lipidomics, cholesterol biosynthesis, medicinal chemistry, drug discovery

## Abstract

Targeting autophagy is believed to hold great promise for the treatment of various diseases, including cancer. However, since the therapeutic efficacy of currently available autophagy-modulating drugs is limited by off-target effects and the requirement of high dosage, there is an urgent need to develop novel autophagy-targeting compounds. In this study, we report molecules of the biarylacetamide class as novel autophagy inhibitors. These molecules were identified via phenotypic high-throughput screening, and a series of analogues was subsequently synthesized. Among these, **5d** and **5j** were retained as potent autophagy blockers in HeLa and LNCaP cells. Both compounds inhibited autophagy at a late-stage in the pathway, as evidenced by the strong accumulation of RFP-GFP-LC3 puncta as well as LC3-II, GABARAP-II and SQSTM1 protein levels, resembling the effects obtained with the well-known late-stage autophagy inhibitor Bafilomycin A1. Quantitative proteome profiling combined with metabolomic and lipidomic studies revealed that **5j** significantly altered lipid metabolism. These alterations included activation of the cholesterol biosynthesis pathway and changes in the distribution of key lipid classes, such as phospholipids, ceramides and triglycerides. Further mechanistic studies indicated that **5d** and **5j** triggered an ER stress response and may impair lysosomal function, as suggested by the accumulation of pro-cathepsin D. Collectively, these findings demonstrate that **5j** is a novel and potent late-stage autophagy inhibitor with a distinct mechanism of action compared to currently available inhibitors.

## Introduction

Macroautophagy, hereafter termed autophagy, maintains cellular homeostasis by removing superfluous or damaged proteins and organelles. This cellular waste is engulfed within double-membrane vesicles known as autophagosomes, which then fuse with lysosomes, forming autolysosomes, where the cargo is degraded and recycled into valuable building blocks such as amino acids, lipids, and sugars that fuel metabolic processes^[1–4]^. Autophagy ensures cellular resilience under stress conditions and safeguards against malignant growth. However, in an established tumor, autophagy’s recycling mechanisms often support tumor survival and proliferation^[5]^. This way, autophagy can contribute to cancer resistance. Autophagy inhibition has been proposed as a strategy to prevent cancer cells from accessing recycled nutrients, thereby reducing their ability to grow and potentially restoring balance to the cellular ecosystem. Early-stage inhibitors, such as Unc-51-like kinase (ULK1) and vacuolar protein-sorting 34 (VPS34) inhibitors, impair autophagy initiation. Conversely, late-stage inhibitors such as lysosomal inhibitors (V-ATPase inhibitors, protease inhibitors, lysosomotropic agents) block autophagosome-lysosome fusion and/or inhibit lysosomal and autolysosomal degradation^[6,7]^. Chloroquine and its derivatives have shown potential in sensitizing tumor cells to chemotherapy and radiotherapy in preclinical studies^[8,9]^. From these positive outcomes, chloroquine and hydroxychloroquine in combination with classical anticancer therapies were evaluated in clinical trials. These, however, have yielded mixed results due to dose-limiting toxicity and ample off-target effects^[10,11]^. Subsequent *in vitro* and *in vivo* studies further supported these limitations, highlighting toxicity and various cellular alterations^[12,13]^. A novel lysosomotropic agent, GNS561, targeting palmitoyl-protein thioesterase 1 (PPT1), has recently entered clinical trials, however, limited efficacy and adverse side effects have been reported^[14]^. Overall, targeting autophagy in cancer remains a promising strategy to enhance the efficacy of anticancer therapies in advanced cancer, but more specific and effective inhibitors with a higher therapeutic index need to be developed. In this work, we report the discovery of a novel class of autophagy blockers, the biarylacetamides, identified by phenotypic high-throughput screening (HTS). Chemical structure optimization allowed improving potency and removing the cytotoxicity of the derivatives. A multi-omics strategy (proteomics, metabolomics and lipidomics) revealed that our lead compound **5j** resulted in a disruption of lipid metabolism pathways, potentially associated with autophagy impairment. In addition, our data suggested that **5j** triggered an ER stress response, which may further contribute to its mechanism of action.

## Results

### Design and synthesis of biarylacetamide derivatives

**Figure 1.**
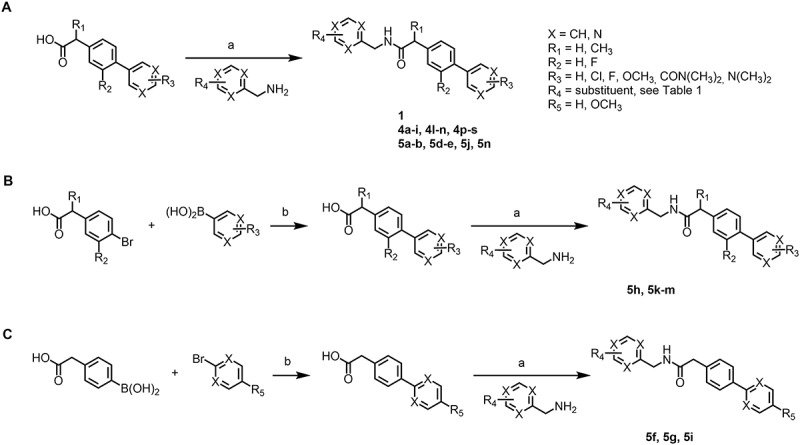
Synthetic routes used to prepare the biarylacetamide derivatives.

We screened a curated library of 10,240 drug-like molecules (Enamine, DDS-10) in L929 fibroblasts stably expressing GFP-LC3 using high-content fluorescence microscopy imaging, to identify autophagy-modulating agents (unpublished results). The compounds were evaluated for their ability to stimulate or inhibit autophagy at 10 µM for 24 h and ranked based on their potential to increase GFP-LC3-positive puncta per cell. Compounds **1** and **2** (Table 1), belonging to the biarylacetamide class, were identified among the most promising autophagy modulators. To explore the chemical space and enhance the potency of the selected derivatives, we applied chemical modifications to the biarylacetamide core in an explorative, diversity-oriented manner. We synthesized 37 novel derivatives divided into three series (Table 1). In series 3, the biaryl group was replaced by an aryl, creating “truncated” derivatives. In series 4, modifications were focused on the *N*-substituent, while in series 5, modifications were introduced on the biaryl scaffold. These modifications were designed to explore various drug-like parameters, which are physicochemical and structural features known to influence a compound’s pharmacokinetics and oral bioavailability. Specifically, we evaluated lipophilicity, hydrophilicity, and acidity/basicity, while fulfilling Lipinski’s rule of five. Most derivatives were prepared using the synthetic route depicted in Figure 1. A major number of final products was obtained by coupling a commercial biarylacetate and an amine (Figure 1A). To further derivatize the biaryl moiety, a Suzuki-Miyaura cross-coupling between commercially available boronic acids and aryl halides was relied on to synthesize a set of biarylacetate derivatives. Final products were subsequently obtained via peptide coupling of a synthesized biarylacetate and a selected commercially available amine (Figure 1B,C).

Reagents and conditions: (a) HATU, DIPEA, DMF, room temperature, 18 h; (b) Pd(PPh_3_)_4_, Cs_2_CO_3_, MeCN:H2O (1:1), 90°C, 24–48 h.

**Table 1.** Chemical Structures of Compound 1 and 2, and the Novel Biarylacetamide Derivatives from Series 1, 2 and 3.

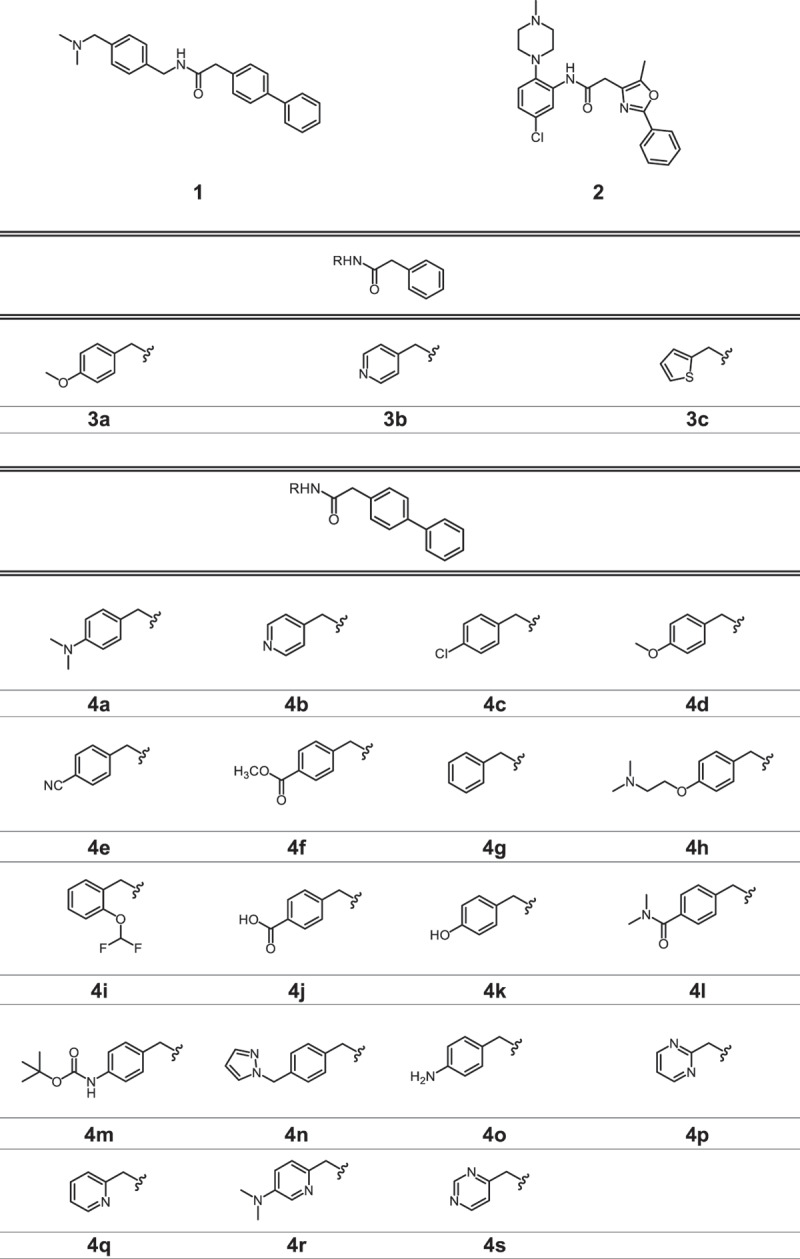

**Table 1.** Chemical Structures of Compound **1** and **2**, and the Novel Biarylacetamide Derivatives from Series 1, 2 and 3. (Continued).

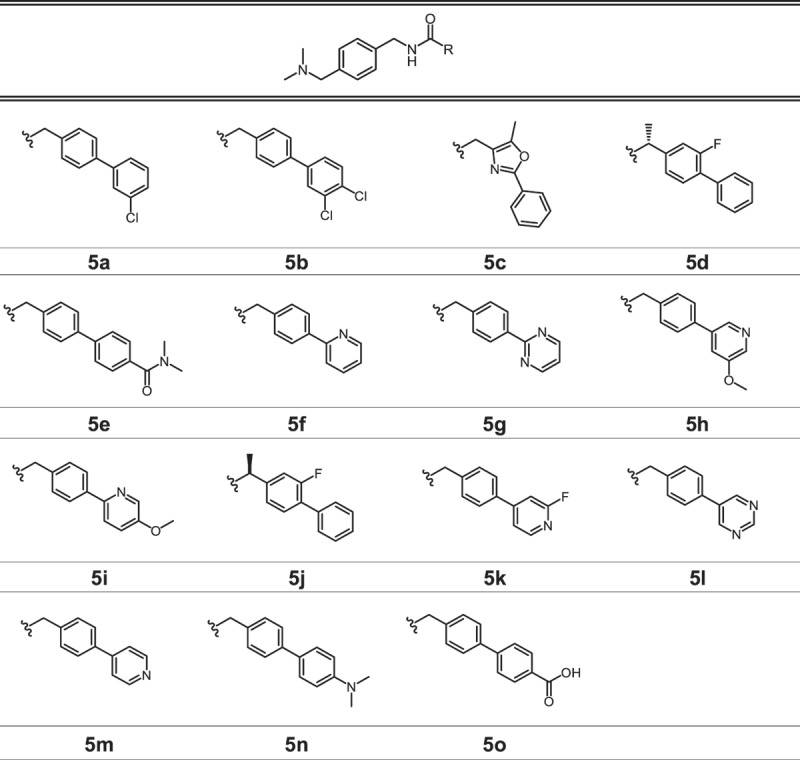

### In vitro biological evaluation

#### Biarylacetamide derivatives have variegated effects on cell viability

**Figure 2.**
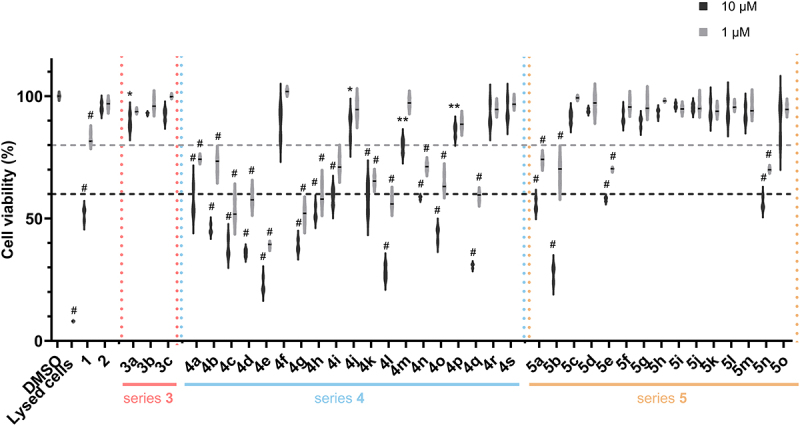
Assessment of biarylacetamides effects on cell viability.

We first assessed the cell viability of HeLa cells exposed to the biarylacetamide derivatives at 1 and 10 µM for 24 h using the cell proliferation reagent WST-1 (Figure 2). Interestingly, compound **1**, HTS-hit **1**, which had not exhibited cytotoxicity in L929 fibroblasts during the HTS campaign in L929 cells, reduced HeLa cells viability to 52% at 10 µM (Figure 2). Neither compound **2** nor Series 3, comprising “truncated” derivatives, exhibited any signs of toxicity. The majority of compounds of series 4, in which modifications were carried out on the *N*-substituent, elicited substantial cytotoxicity. The diversity of substituents used to introduce variation does not allow us to draw a conclusion on specific structural features responsible for the observed cytotoxicity effects. From this series, only **4f**, **4g**, **4m**, **4p**, **4r** and **4s** maintained cell viability at 10 µM. In contrast, series 5, in which modifications were performed on the biaryl moiety, displayed a lower number of molecules with cytotoxic effects. Only **5a**, **5b**, **5f** and **5n** reduced cell viability below 60% at 10 µM, with **5b** (bearing a dichlorobiphenyl, a well-known cytotoxic moiety) being the most cytotoxic compound. To be able to investigate the mechanism of action of the biarylacetamides, we selected compounds with no or negligible cytotoxic effect detected at 10 and 1 µM for further biological studies. In addition, we included a small subset of molecules with statistically significant but relatively limited cytotoxic effects, applying the following cutoff: cell viability > 80% at 1 µM and > 60% at 10 µM. This inclusion was based on the hypothesis that the observed signs of cytotoxicity may be related to alterations to the autophagy flux, warranting further investigation.

HeLa cells were treated with biarylacetamide derivatives at 1 µM or 10 µM for 24 h. Cell viability was assessed using WST-1 reagent. Lysed cells were used as negative control, and DMSO (control) was set to 100% cell viability. The bar plot represents the mean ± SD of three independent experiments, *p*-values were calculated by Tukey’s HSD test (*, *p* < 0.05; **, *p* < 0.01; #, *p* < 0.0001).

#### The biarylacetamide derivatives block autophagic flux in HeLa and LNCaP cells

**Figure 3.**
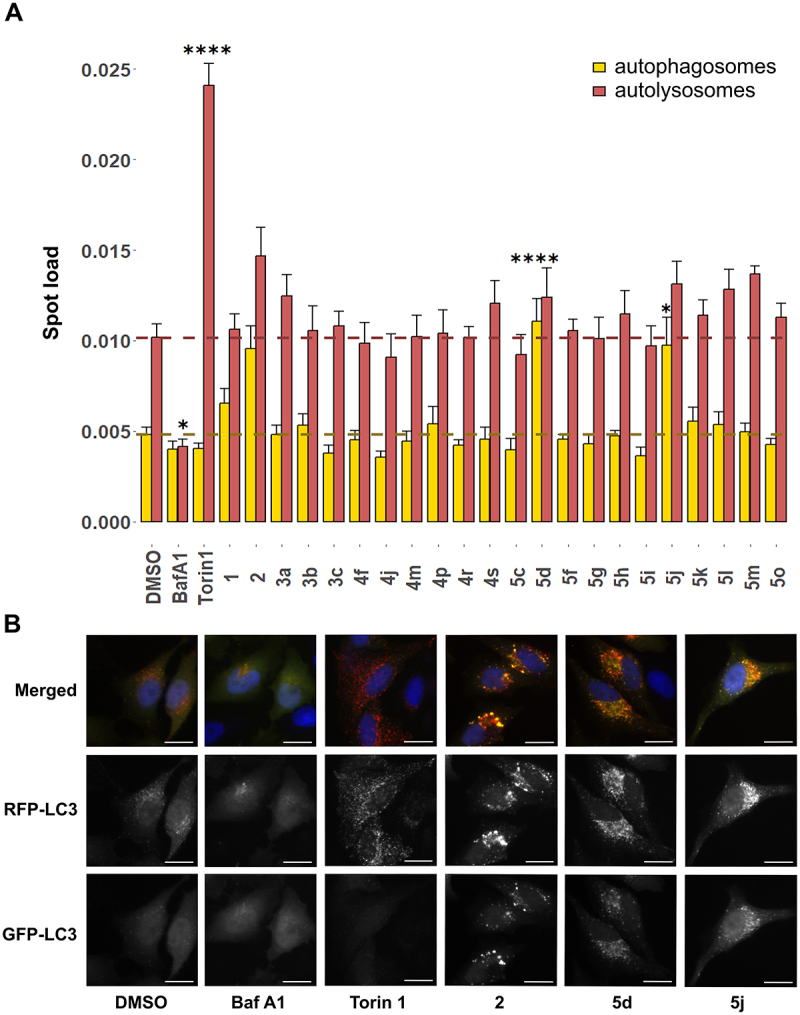
Biarylacetamide derivatives inhibit the autophagic flux at a late-stage in RFP-GFP-LC3 HeLa cells.

We examined the effect of the 22 selected derivatives on autophagic flux using high-content fluorescence microscopy in HeLa cells stably expressing an RFP-GFP-LC3 construct (HeLa-Difluo^TM-^hLC3)^[15]^. This model system relies on the selective quenching of GFP fluorescence in the lysosomal environment due to its low pH and protease content, whereas RFP is more resistant to this environment and retains fluorescence. Therefore, the colocalization of RFP and GFP signals (yellow puncta) represents autophagosomes and RFP signal (red puncta only) autolysosomes. The autophagy inducer Torin 1, and inhibitor Bafilomycin A1 (Baf A1) were used as positive and negative controls, respectively. Treatment with Torin 1 drastically increased the number of red-only puncta (autolysosomes) compared to DMSO-control-treated cells, indicative of enhanced autophagic flux (Figure 3A). Conversely, treatment with Baf A1 (for the last 2 h) significantly decreased the number of autolysosomes. As a technical note, while 2 h Baf A1 treatment was not sufficient to significantly increase the number of yellow puncta in this experiment, a longer treatment with Baf A1 (18 h) very potently increased the autophagosome (yellow) spot load, along with decreased autolysosome (red-only) spot load, as expected (Figure S1). In contrast to Torin 1, treatment with HTS-hits **1** and **2** led to an increase in yellow puncta compared to DMSO, suggesting an accumulation of autophagosomes. Notably, among the novel derivatives, **5d** and **5j** emerged as strong hits in this screening, with both strongly and significantly increasing the number of yellow puncta compared to control. Interestingly, treatment with **5d**, **5j** and **2** resulted in clustered puncta localized in the perinuclear region of cells, contrasting with Torin 1 treatment, where puncta were dispersed throughout the cell (Figure 3B). These observations suggest an accumulation of autophagosomes in the perinuclear region, potentially indicating an impairment of the autophagosome-lysosome fusion, a critical process that typically occurs in this area. Overall, these results already suggest that the biarylacetamide derivatives inhibit autophagic flux.

HeLa cells stably expressing the RFP-GFP-LC3 reporter were treated with the biarylacetamide derivatives at 10 µM for 24 h, or Torin 1 (1 µM, 24 h), or Baf A1 (161 nM, 2 h). A. The bar graph represents spot load ([puncta number per cell × puncta area mean per cell]/cell area) of autophagosomes (yellow) and autolysosomes (red) per cell. The mean ± SEM of three independent experiments is plotted, *p*-values were calculated by Tukey’s HSD test (*, *p* < 0.05; ****, *p* < 0.0001). B. Representative fluorescence images of RFP-GFP-LC3 HeLa cells treated with controls (DMSO, Baf A1, Torin 1) or **2**, **5d**, **5j**. Nuclei were stained with Hoechst, scale bar = 20 µm.

We attempted to draw a structure-activity relationship of the biarylacetamide derivatives by comparing the chemical features and biological activities of the novel derivatives from series 3, 4, and 5 with HTS-hit **1**. The new hits **5d** and **5j** are enantiomers and differ from HTS-hit **1** by the addition of a methyl group on the acetamide moiety and a fluorine in position 2 of the biaryl. These modifications, which increase lipophilicity of the compounds, appear to be beneficial for enhancing autophagy inhibition. This observation, however, might also be related to a lipophilicity-driven increase in cell permeability. In contrast, most of the other derivatives from series 3, 4 and 5 exhibited autophagosome and autolysosome levels comparable to DMSO. In series 3, comprising “truncated” derivatives, molecule **3a** showed a non-statistically significant trend toward increased autolysosomes levels relative to control, possibly through a distinct mechanism than HTS-hit **1** and **2** (Figure 3A). In series 4, only **4s**, featuring a 4-pyrimidinyl group and lacking the dimethylaminomethyl moiety compared to HTS-hit **1**, demonstrated a modest but non-statistically significant increase in autolysosome levels compared to DMSO. Within series 5, several derivatives with biaryl heteroaromatic substitutions, such as **5h** (biphenyl replaced by 4-(5-methoxypyridin-3-yl)phenyl), **5l** (biphenyl replaced by 4-(pyrimidin-5-yl)phenyl) and **5m** (biphenyl replaced by 4-(pyridin-4-yl)phenyl) showed increased autolysosome levels compared to control. However, none of these achieved statistical significance. Compound **5o**, bearing a para-carboxylic acid moiety which adds hydrophilicity to the biphenyl scaffold, showed a slight but not statistically significant increase in autolysosomes levels. Overall, except for **5d** and **5j**, none of the novel biarylacetamide derivatives resulted in a relative increase in autophagosome levels (yellow puncta). Based on these findings, we selected compounds **5d** and **5j**, along with HTS-hit **1** and **2** for further exploration.

**Figure 4.**
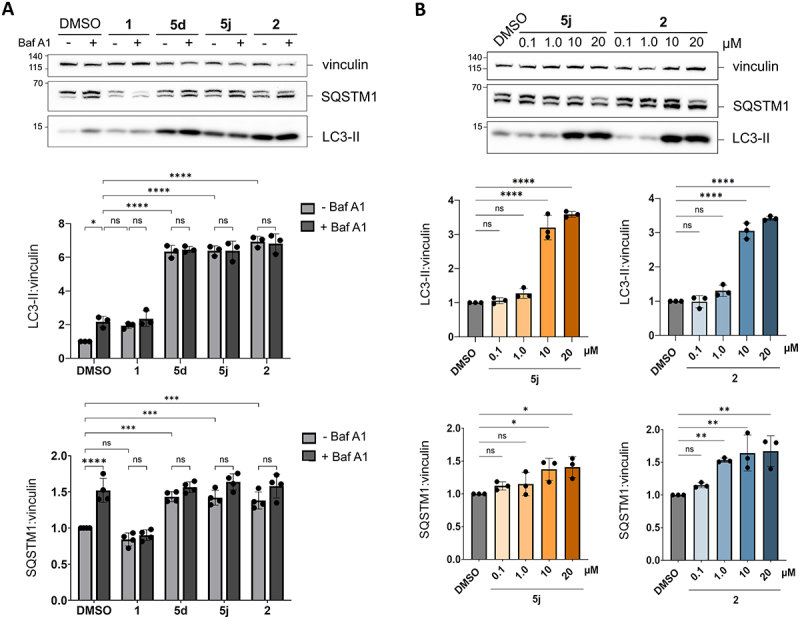
Biarylacetamide derivatives inhibit autophagic flux at a late-stage in HeLa cells.

To further assess the effect of the compounds on autophagy, we monitored the protein levels of several autophagy markers in HeLa cells by western blotting. During autophagy, cytoplasmic LC3-I is lipidated to LC3-II and thereby covalently attached to the autophagosomal membrane and subsequently degraded in autolysosomes. When the autophagic flux is inhibited at a late-stage, LC3-II attached to the inner membrane of autophagosomes or autolysosomes is no longer degraded, leading to LC3-II accumulation. Co-treatment with Baf A1 allows for assessment of autophagic flux. Treatment with compounds **1**, **2**, **5d**, and **5j** at 10 µM for 24 h resulted in increased LC3-II levels compared to DMSO (Figure 4A). Co-treatment with Baf A1 (the last 2 h of treatment) and the biarylacetamide derivatives failed to further increase LC3-II levels compared to biarylacetamide treatment alone (Figure 4A). SQSTM1/p62, a cargo adaptor protein that is degraded in autolysosomes, also serves as an autophagy marker. Elevated levels of SQSTM1 indicate a potential blockade of the autophagic flux. Notably, treatment with **5d**, **5j** and **2** significantly increased SQSTM1 levels compared to DMSO, while compound **1** did not (Figure 4A). Furthermore, co-treatment with Baf A1 did not significantly further increase SQSTM1 levels (Figure 4A). These results suggest that autophagosomes accumulate without being degraded. Compounds **5j** and **2** blocked autophagy (as assessed by accumulation of LC3-II and SQSTM1) in a dose-dependent manner (Figure 4B).

(A) Western blot analysis of SQSTM1/p62 and LC3-II levels in HeLa cells treated with DMSO, **1**, **2**, **5d**, **5j** (10 µM) for 24 h in the presence or absence of Baf A1 (the last 2 h of treatment). The bar graphs represent the mean ± SD of minimum three independent experiments, *p*-values were calculated by HSD Tukey’s test (ns, non-significant; *, *p* < 0.05; ***, *p* < 0.001; ****, *p* < 0.0001). (B) Western blot analysis of SQSTM1/p62 and LC3-II levels in HeLa cells treated with DMSO, 5j and 2 (0.1, 1.0, 10, 20 µM) for 24 h. The bar graphs represent the mean ± SD of three independent experiments, *p*-values were calculated by HSD Tukey’s test (ns, non-significant; *, *p* < 0.05; **, *p* < 0.01; ***, *p* < 0.001; ****, *p* < 0.0001).

**Figure 5.**
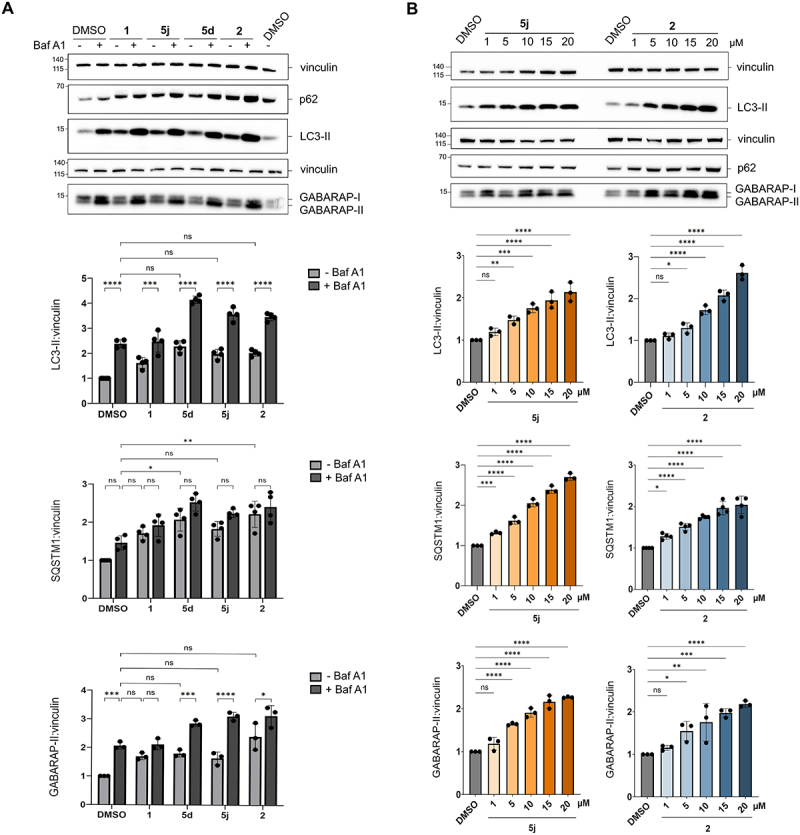
Biarylacetamide derivatives inhibit autophagic flux at a late-stage in LNCaP cells.

To explore the generality of these observations, we reproduced these experiments in LNCaP cells, a human prostate adenocarcinoma cell line. Treatment with **1**, **2**, **5d** and **5j** increased LC3-II levels compared to DMSO. Interestingly, co-treatment with Baf A1 (added the last 2 h of treatment) and the biarylacetamides further increased LC3-II levels but not SQSTM1 levels (Figure 5A). We also examined GABARAP, which, similarly to LC3, is a mammalian orthologue of yeast Atg8 that is subjected to lipidation to autophagosomal membranes and degradation in autolysosomes. The results obtained for GABARAP-II mirrored those obtained for LC3-II (Figure 5A). Treatment with **5d**, **5j**, and **2** (alone) resulted in comparable increases in LC3-II and GABARAP-II levels to those observed with Baf A1. Dose-dependent effects of the biarylacetamides on the accumulation of these three autophagy markers (LC3-II, SQSTM1, and GABARAP-II) were also confirmed in LNCaP cells (Figure 5B). Together, these results indicate that **5d**, **5j** and **2** induce a virtual complete block in autophagic processing of the LC3-II, SQSTM1, GABARAP-II autophagy markers in HeLa cells, while they induce a strong, yet incomplete block in LNCaP cells. Similarly, **5j** partially inhibited autophagic flux in mouse embryonic fibroblasts (MEFs), as revealed by accumulation of LC3-II without a corresponding decrease in SQSTM1 levels, which contrasts with the observed decrease obtained with the autophagy inducer Torin 1 (Figure S2). The compounds thus appear to inhibit autophagy at a late stage across different cell types, albeit with different potencies.

(A) Western blot analysis of LC3-II, SQSTM1/p62, and GABARAP-II levels in LNCaP cells treated with DMSO, **1**, **2**, **5d**, and **5j** (10 µM) for 24 h in the presence or absence of Baf A1 (only last 2 h of treatment). The bar graphs represent the mean ± SD of minimum three independent experiments, *p*-value were calculated by HSD Tukey’s test (ns, non-significant; *, *p* < 0.05; ***, *p* < 0.001; ****, *p* < 0.0001). (B) Western blot analysis of LC3-II, SQSTM1/p62, and GABARAP-II levels in LNCaP cells treated with DMSO; **5j** and **2** (0.1, 1.0, 10, 20 µM) for 24 h. the bar graphs represent the mean ± SD of three independent experiments, *p*-value were calculated by HSD Tukey’s test (ns, non-significant; *, *p* < 0.05; **, *p* < 0.01; ***, *p* < 0.001; ****, *p* < 0.0001).

To validate that the increased levels of LC3-II, SQSTM1 and GABARAP-II observed in compound-treated cells were not due to increased autophagic flux, we employed retinal pigment epithelial cells stably expressing a tetracycline-inducible lactate dehydrogenase B (LDHB)-mKeima probe (LDHB-mKeima RPE-1). This probe is used to assess bulk autophagic cargo flux^[16]^. Upon reaching the lysosomal environment, the fluorescence excitation maximum of LDHB-mKeima shifts, allowing ratiometric quantification of the probe’s flux. In this assay, treatment with compounds **5d**, **5j**, and **2** did not increase the acidic ratio of LDHB-mKeima, confirming that the derivatives do not stimulate autophagic cargo flux (Figure S3). Instead, and in line with the compounds decreasing basal autophagic flux, a tendency of a slight decrease in acidic ratio of LDHB-mKeima was observed with **5d**, **5j** and **2**. Of note, RPE-1 cells have relatively low levels of basal autophagy, whereas autophagy is readily induced by Torin 1^[16,17]^. In agreement, and as a control for the assay, treatment with Torin 1 induced a substantial SAR405-sensitive increase in LDHB-mKeima flux (Figure S3).

The novel biarylacetamide derivatives **5d** and **5j** appear to be promising autophagy inhibitors. We selected compound **5j** for further investigation of the molecular effects of this compound class.

#### 5j stimulates the cholesterol biosynthesis pathway

**Figure 6.**
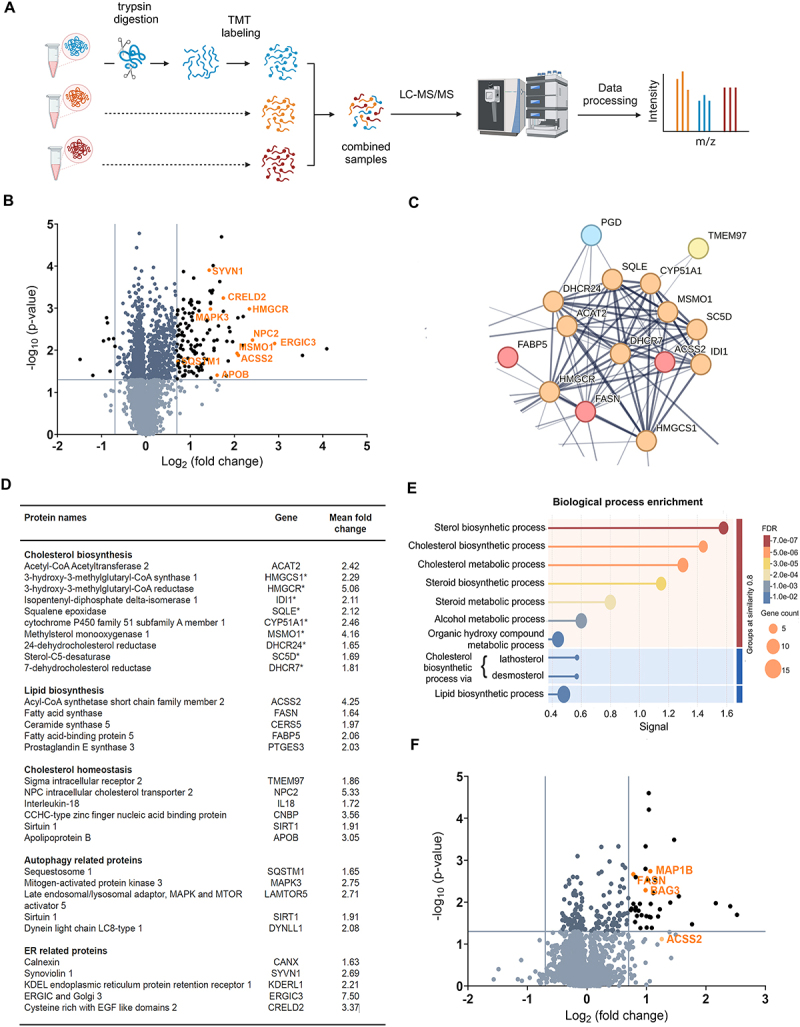
Proteins and pathways regulated by compound **5j**.

To explore the potential mechanism of action by which compound **5j** inhibits autophagy, we performed a quantitative proteome and phosphoproteome profiling using tandem mass tag (TMT) labeling combined with liquid chromatography-tandem mass spectrometry (LC-MS/MS) for the quantification of phosphopeptides, non-phosphopeptides and associated proteins in HeLa cells (Figure 6A)^[18,19]^. Over 5,000 proteins and 2,000 phosphoproteins were identified in the samples of cells treated with **5j** (10 µM, 18 h) and DMSO as control (Tables S1, S2).

A. Schematic illustration of proteomics workflow using TMT-labeling. B. Proteomics analysis of proteins differentially expressed in HeLa cells treated with **5j** and DMSO for 18 h (three independent experiments), represented as a volcano plot. Proteins with *p*-value < 0.05 (threshold) and log_2_ fold change < −0.7 (threshold) or log_2_ fold change > 0.7 (threshold) were considered to be significantly changed in abundance; *p*-values were calculated using two-sided Student’s *t*-test. C. Zoom in on the protein–protein interactions network analysis of the significantly upregulated proteins performed using STRING (full network can be found in supplemental materials Figure S4). Cluster of proteins linked to cholesterol and lipid biosynthesis. D. List of proteins significantly upregulated by **5j** treatment with their fold change and related pathway(s). Proteins highlighted with ”*” are associated with the cholesterol biosynthesis pathway and lipid biosynthesis pathway. E. Plot showing the top 10 biological processes (Gene Oncology) of the significantly upregulated proteins by **5j** performed with STRING. F. Volcano plot of phosphoproteomics analysis of phosphosites associated proteins differentially expressed in HeLa cells treated with **5j** and DMSO for 18 h (three independent experiments). Phosphosites with *p*-value < 0.05 (threshold) and log_2_ fold change < −0.7 (threshold) or log_2_ fold change > 0.7 (threshold) were considered to be significantly changed in abundance; *p*-values were calculated using two-sided Student’s *t*-test.

We first evaluated the effect on the proteome. To focus on relevant changes, we only retained up- or downregulated proteins whose log_2_ fold changes were < −0.70 and > 0.70 with *p* < 0.05, corresponding to the blue horizontal and vertical thresholds on the volcano plot in Figure 6B (Table S3). By these criteria, 8 proteins were downregulated, and 125 proteins were upregulated. Among the upregulated proteins was SQSTM1, which is consistent with our western blot results, validating the impact on the autophagy pathway. Protein-protein interaction network analysis (performed using STRING, v12, https://string-db.org/) of the significantly upregulated proteins revealed a cluster of proteins involved in the sterol biosynthesis pathway (Figure 6C, Figure S4). This cluster includes 10 proteins directly associated with the cholesterol biosynthesis process (Figure 6C,D). Additionally, 11 other upregulated proteins are related to lipid biosynthesis and cholesterol homeostasis (in Figure 6D). Enrichment analysis further supported the predominance of sterol biosynthesis and cholesterol metabolism pathways among the upregulated proteins (Figure 6E). Interestingly, some autophagy- and ER-related proteins were also significantly upregulated (Figure 6D).

Next, we identified 37 significantly upregulated phosphosites in cells treated with **5j** (Figure 6F and Table S4). Among the associated phosphoproteins were fatty acid synthase (FASN; T2204) and microtubule-associated protein 1B (MAP1B; S1298, T2034) (Figure 6F). The phosphorylation of FASN may reflect increased FASN activity^[20]^. MAP1B is a microtubule-associated protein involved in autophagy, and its upregulated phosphorylation at two sites may be indicative of a change in microtubule stability^[21]^ and might conceivably affect its autophagy-regulatory function, thus helping explain the effects of **5j** on autophagy. Notably, no significantly downregulated phosphoproteins were detected.

Collectively, these findings indicate that **5j** modulates lipid metabolism, including the sterol biosynthesis pathway, which may affect autophagy, as further discussed below. Moreover, altered expression (and phosphorylation levels) of several autophagy-regulatory and ER-related proteins by **5j** provides additional information to the potential mechanism of how **5j** mediates its late-stage inhibition of autophagic flux.

#### 5j dysregulates lipid metabolism

**Figure 7.**
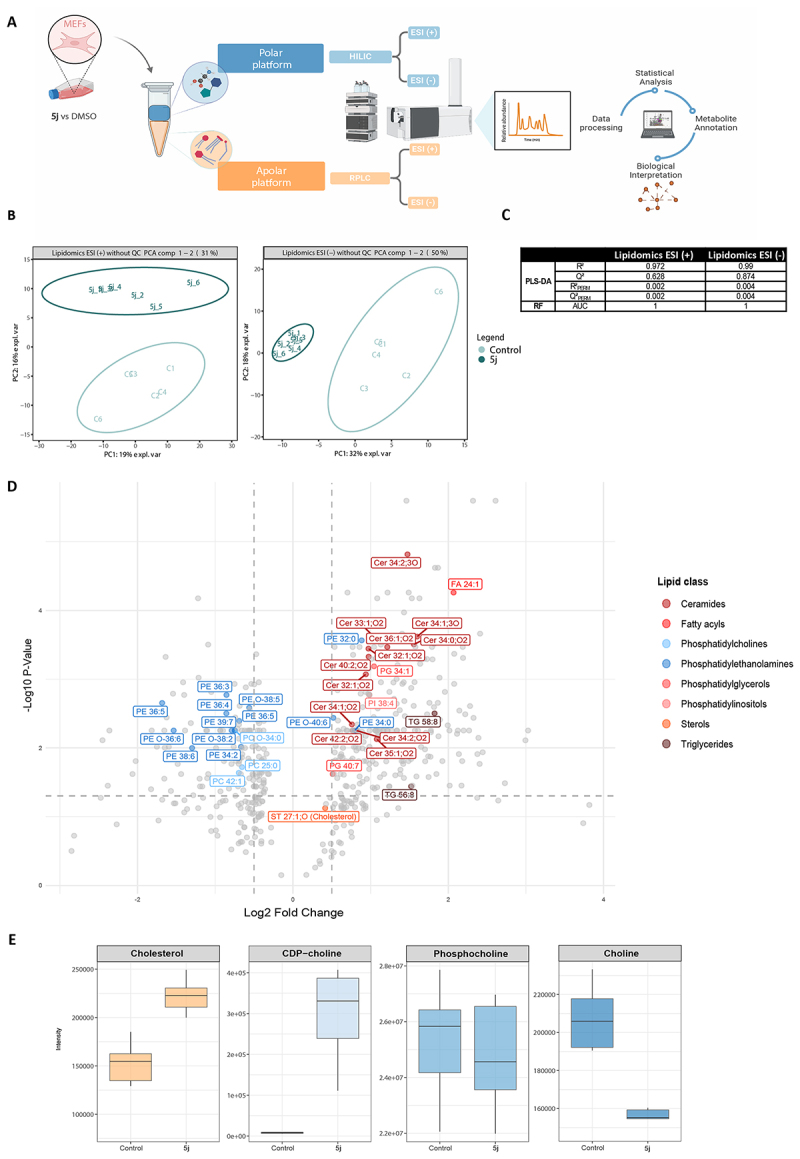
Metabolites regulated by compound **5j**.

To further investigate the effects of compound **5j**, an untargeted metabolomics experiment was performed for exploration of its influence on the metabolome of mouse embryonic fibroblasts (MEFs). MEFs were selected for this study as our metabolomic and lipidomic methods had been previously developed and validated in these cells using known autophagy modulators. Figure 7A provides an overview of the experimental workflow. MEFs were treated with **5j** (10 µM, *N* = 6) or DMSO (0.1%, *N* = 6) for 18 h. Polar metabolites and lipids were extracted and analyzed using a multi-platform approach based on LC-high resolution MS^[22]^.

A. Schematic overview outlining the metabolomics workflow used with MEFs. B. Lipidomics principal component analysis (PCA) plots showing the clustering patterns of lipid profiles in apolar fractions of MEF cells treated with compound **5j** or DMSO for 18 h. C. Multivariate model evaluation. Model performance was evaluated by R^2^, Q^2^, R^2^_PERM_, and Q^2^_PERM_ for PLS-DA, and by AUC for random forest (RF). D. Volcano plot displaying features that were selected based on multivariate statistical analysis. Lipids meeting the criteria of *p*-value < 0.05 and log_2_ fold change thresholds > 0.5 or < −0.5 were considered statistically up- or downregulated. Lipid classes of interest were highlighted with colored dots. E. Boxplots of cholesterol, CDP-choline, choline, and phosphocholine in MEF cells treated with 5j or DMSO for 18 h.

A curated data matrix was prepared for statistical analysis using strict preprocessing criteria. The principal component analysis (PCA) plots of the lipid fraction showed both inter- and intra-group variability (Figure 7B). The first two components (PC1 and PC2) revealed a clear separation between the exposed group (dark green) and control group (light green), suggesting significant lipid profile alterations induced by compound **5j**. For polar metabolites (Figure S5), PC1 and PC2 showed a separation between the control group and **5j** treatment group in electron spray ionization positive mode (ESI (+)). However, for ESI negative mode, an overlap was observed between the two groups, indicating weaker difference after **5j** treatment in this analytical platform. Overall, these findings demonstrated a substantial metabolic impact of compound **5j**, particularly evident in the lipid fractions.

To identify the most discriminative lipids following **5j** treatment, we performed multivariate statistical analyses using Partial Least Squares Discriminant Analysis (PLS-DA) and random forest (RF) models (further described in Methods). The reliability of these models was confirmed, both models showed strong performance in distinguishing treated from control samples, with minimal risk of overfitting (Figure 7C). Key lipids were selected for further analyses based on their importance in both models, specifically, those with a Variable Importance in Projection (VIP) score above 1 in PLS-DA and with a Variable Importance Measure (VIM) above 0.1 in the RF model. Lipids were visualized with a volcano plot (Figure 7D), lipids with a *p*-value < 0.05 and a log_2_ fold change > 0.5 or < −0.5 were considered significantly altered. Lipid classes were color-coded to highlight the different classes. This analysis revealed several lipid classes that were significantly increased or decreased following **5j** treatment.

Upregulated lipids included multiple ceramide (Cer) species, triglycerides (TG), phosphatidylglycerols (PG), phosphatidylinositol (PI) 38:4 | PI 18:0_20:4 and fatty acid (FA) 24:1. Notably, ceramides containing a hydroxy group on the fatty acyl chain, such as Cer 18:1;2O/16:0;(2OH) and Cer 34:2;3O (class Cer_AS/Cer_BS), exhibited a higher fold change compared to non-hydroxylated ceramides. Additionally, Cer 18:0;O2/16:0, a dihydroceramide, showed the second highest fold change among the ceramide classes. Dihydroceramides are precursors in the *de novo* synthesis of ceramides, suggesting that **5j** may upregulate this pathway and thereby increase the levels of multiple ceramide species. Upregulation of PI 18:0_20:4 May indicate increased demand for phosphatidylinositol phosphates (PIPs) or disruptions in its conversion to PIPs. The latter are essential in early autophagy, orchestrating the recruitment and activation of autophagy-related proteins during phagophore nucleation and membrane expansion^[23]^. PIPs are also involved in autophagosome-lysosome fusion^[24]^. Disrupted PIPs turnover could impair these processes, thereby inhibiting autophagic flux. However, more dedicated analytical methods are needed to detect these PIPs and draw further conclusions on their regulation after **5j** treatment.

Conversely, downregulated lipids included multiple (ether)-phosphatidylethanolamine (PE) and (ether)-phosphatidylcholine (PC) species. Despite the overall downregulation of PE species, specific unsaturated PE species such as PE 34:0 (PE 16:0_18:0) and PE 32:0 were significantly upregulated following **5j** exposure, suggesting a redistribution of membrane phospholipids. This shift might reflect autophagy inhibition, as reduced PCs biosynthesis is associated with impairment in autophagosome completion^[25]^ and PE is important for lipidation of LC3s and GABARAPs.

Compound **5j** induced varying responses in polar metabolites related to lipid metabolism as shown in Figure 7E. CDP-choline, choline, and phosphocholine displayed distinct responses to **5j** treatment. CDP-choline demonstrated a significant upregulation, whereas choline was significantly downregulated. Phosphocholine displayed no definitive trend, indicating a relatively stable response. This observation points to a perturbation in CDP-choline pathway, which is crucial for PC synthesis and which could potentially alter membrane composition and cellular signaling pathways related to lipid metabolism. An increase in cholesterol (ST 27:1;O) levels was detected, which aligns with the observed upregulation of cholesterol biosynthesis in our proteomics data. However, despite being identified in the multivariate analysis, this change was not statistically significant in the univariate analysis.

To validate lipid identification, mirror plots of authentic standards and acquired MS/MS spectra were generated for Ceramide 18:1;2O/16:0 (collision energy 20) and cholesterol (collision energies 10 and 20) (Figure S6). A strong spectral match was observed for both lipids, confirming their identification in our lipidomics data.

These results support previous findings that compound **5j** substantially alters lipid metabolism and further suggest a disruption in lipid homeostasis. Of note, however, it remains to be determined whether similar alterations will be induced also in other cell lines than in MEFs.

#### 5j and 5d cause ER stress

Our proteomics data revealed the upregulation of several ER-related proteins, following **5j** treatment. These included cysteine-rich EGF-like Domain 2 (CRELD2), a protein secreted during ER stress^[26]^; calnexin (CANX), a molecular chaperone involved in protein folding quality control^[27,28]^, and synoviolin 1 (SYVN1), an E3 ubiquitin ligase that mediates the degradation of misfolded proteins at the ER^[29,30]^. In parallel, our lipidomic data suggested potential lipid membrane remodeling evidenced by altered distribution of several membrane phospholipid classes. Building on these findings, we hypothesized that **5j** might cause ER stress, which in turn activates the unfolded protein response (UPR).

**Figure 8.**
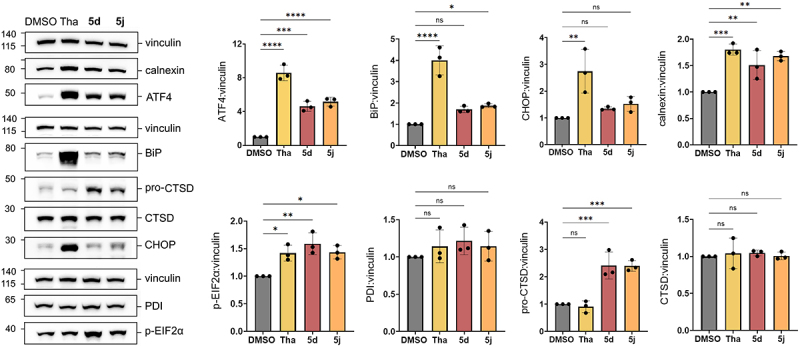
Compound **5j** and **5d** cause ER stress and accumulation of pro-cathepsin D.

To test this hypothesis, we assessed the expression of canonical UPR markers in HeLa cells by western blotting. Thapsigargin, a well-known, highly potent ER stress inducer and autophagy inhibitor^[31]^, was used as positive control. Treatment with thapsigargin (Tha, 100 nM) led to a strong and significant increase in BiP (GRP78), ATF4 and CHOP, along with elevated levels of phosphorylated IF2α and calnexin, but not of protein disulfide isomerase (PDI) (Figure 8). Treatment with **5d** and **5j** significantly increased ATF4, phosphorylated eIF2α and calnexin levels. BiP and CHOP were also upregulated, although the increase only reached statistical significance for BiP after **5j** treatment. These results suggest that **5d** and **5j** induce ER stress, albeit to a lesser extent than thapsigargin.

Western blot analysis of ATF4, BiP/GRP78, CHOP, calnexin, phosphorylated IF2α; PDI, and (pro-)cathepsin D protein levels in HeLa cells treated with DMSO, thapsigargin (100 nM), **5d**, and **5j** (10 µM) for 18 h. The bar graphs represent the mean ± SD of three independent experiments, *p*-value were calculated by HSD Tukey’s test (ns, non-significant; *, *p* < 0.05; **, *p* < 0.01; ***, *p* < 0.001; ****, *p* < 0.0001).

Given the established relationship between late-stage autophagy inhibition and lysosomal function, we next evaluated the effects of **5d** and **5j** on cathepsin D, a lysosomal protease involved in autophagosome maturation and protein degradation. No changes were observed in cathepsin D levels after **5d** and **5j** treatments (Figure 8). However, unlike thapsigargin, both compounds significantly increased levels of pro-cathepsin D (Figure 8), suggesting a defect in its processing and/or altered trafficking in response to biarylacetamide treatment.

Overall, these findings demonstrate that **5d** and **5j** trigger an ER stress response and interfere with cathepsin D maturation, potentially contributing to altered lysosomal trafficking. These results provide additional mechanistic insights into the late-stage autophagy inhibition observed with biarylacetamides.

## Discussion

In this study, we identified biarylacetamide derivatives as novel autophagy inhibitors. Chemical structure optimization allowed improving cell viability, drug-like properties and potency, yielding compounds **5d** and **5j** as the most promising molecules. We showed that these molecules impaired late-stage autophagic flux in HeLa and LNCaP cells, leading to accumulation of autophagosomes, as evidenced by clustered and accumulated yellow (RFP-GFP) LC3 puncta and increased LC3-II, GABARAP-II and SQSTM1 protein levels. We attempted to uncover the underlying molecular mechanism(s) by which **5j** inhibits autophagy by employing multi-omics approaches. Quantitative proteome profiling in HeLa cells revealed upregulation of pathways involved in sterol biosynthesis, particularly cholesterol biosynthesis, as well as broader lipid metabolism. These findings were supported by lipidomics data in MEF cells, which showed elevated levels of cholesterol, multiple ceramide species, triglycerides, phosphatidylglycerols, PI 18:0_20:4, FA 24:1 and CDP-choline. Further investigations revealed that **5d** and **5j** induced an ER stress response, as demonstrated by increased expression of UPR markers. In addition, both compounds interfered with cathepsin D maturation, which may contribute to the observed blockage of the autophagic flux.

Of note, there could be a functional or even causal link between the observed autophagy blockade and metabolic changes, which will be further discussed below. However, it should be emphasized that the number of omics studies focusing on autophagy or autophagy modulators remains limited. The lack of reliable reference data complicates the interpretation of our results and underscores the need for more (multi-)omics studies in autophagy research.

The metabolic changes observed following **5j** treatment could result from various cellular alterations. Multiple studies have reported that elevated cholesterol is associated with lysosomal dysfunction leading to autophagy impairment^[32–36]^. In these studies, cholesterol accumulates within the lysosome and the lysosomal membrane, reducing lysosomes’ ability to fuse with other membranes, such as the autophagosome membrane. In our study, it is plausible that compound **5j** leads to a similar cholesterol buildup in the lysosomes and/or at the lysosomal membrane, eventually impairing the autophagosome-lysosome fusion, contributing to autophagy inhibition. Similar effects were described for the synthetic cannabinoid, CP-55,940, which promotes cholesterol biosynthesis genes via SREBP2 activation, impairing intracellular cholesterol trafficking and late-stage autophagy^[36]^. Further supporting lysosomal involvement, we observed a significant accumulation of pro-cathepsin D after **5d** and **5j** treatment, while mature cathepsin D levels were unchanged. This observation may indicate a partial defect in processing pro-cathepsin D, potentially reflecting lysosomal dysfunction^[37]^. Additionally, a recent study has shown that indatraline and sertraline promote accumulation of cholesterol and potentially interfere with cholesterol binding to the NPC intracellular cholesterol transporter NPC1 and NPC2^[38]^. The intracellular cholesterol transporters NPC1 and NPC2 play a crucial role in cholesterol trafficking and transport by facilitating its export from the lysosomes to other cellular compartments for membrane synthesis^[39–41]^. Interestingly, our proteomics data showed an upregulation of NPC2 following **5j** treatment, suggesting a cellular response to enhance cholesterol trafficking capacity to potentially restore cholesterol transport or clear excess cholesterol from lysosomes. Overall, these observations indicate that **5j** may disrupt autophagic flux through a distinct mechanism involving cholesterol accumulation and lysosomal dysfunction.

One other potential hypothesis on the underlying mechanism of these metabolic alterations is ER stress. The unfolded protein response (UPR), triggered during ER stress, orchestrates a set of signaling cascades to restore ER functions. UPR activation disturbs lipid metabolism by promoting lipogenesis, through *de novo* synthesis of ceramides, cholesterol and fatty acids^[42,43]^. Elevated levels of lipids, particularly ceramides, are often linked to lipotoxicity and cellular dysfunction^[44–49]^. These lipids may also accumulate at the ER, exacerbating ER stress and creating a detrimental feedback loop. In line with **5j** putatively inducing an ER stress response, our proteomics data revealed increased levels of several ER-associated proteins. These included cysteine-rich with EGF-like domain protein 2 (CRELD2), a protein secreted during ER stress^[26]^; calnexin (CANX), a protein involved in quality control of protein folding, and synoviolin 1 (SYVN1), an E3 ubiquitin ligase that degrades misfolded proteins accumulating at the ER^[27–30]^. Supporting these findings, we detected increased protein levels of canonical UPR markers such as phosphorylated IF2α, BiP, ATF4 and calnexin after **5d** and **5j** treatment. We also observed alterations in lipid metabolism consistent with ER stress. Our lipidomics data showed elevated levels of various lipids, and particular upregulation of multiple ceramide species. Dihydroceramides, precursors in the *de novo* synthesis of ceramides, were upregulated, as well as ceramide synthase 5 (CERS5), a key enzyme involved in both sphingolipids salvage and *de novo* synthesis pathways, suggesting that the ceramide biosynthesis pathway might be activated after **5j** treatment. Additionally, our analysis also indicated shifts in membrane phospholipids distribution (PCs, PEs, PGs and PI) with selective preservation of saturated PE species, potentially indicating membrane rigidity or remodeling processes. Furthermore, we detected an accumulation of CDP-choline, a precursor for the *de novo* synthesis of PC lipids, while downstream PC levels were reduced. These findings point toward altered membrane lipid dynamics, which may reflect an adaptive response to ER stress or impaired autophagosome biogenesis and completion^[25,47,50]^. Overall, these observations suggest an adaptive cellular response to ER stress, potentially contributing to the lipidomic and metabolic changes observed in our study.

This study illustrates a possible interplay between lipid metabolism, ER stress and/or impaired autophagy. A crosstalk between ER stress and autophagy has already been established: different ER stress pathways can either stimulate or inhibit autophagy, and autophagy inhibition can exacerbate ER stress^[51–54]^. These interconnected processes might trigger additional cellular pathways to mitigate stress and restore homeostasis, at least to the point that **5j** does not result in measurable cellular cytotoxicity after 24 h of treatment.

In summary, we identified specific biarylacetamides as a novel class of autophagy late-stage inhibitors. Chemical optimization improved potency and drug-like properties, yielding **5d** and **5j** as novel biarylacetamide derivatives with strong autophagy inhibitory effects across different cell types. Further chemical and biological efforts to optimize their potency are underway. Our multi-omics analyses suggested that **5j** alters lipid metabolism, as evidenced by the activation of the cholesterol biosynthesis pathway and change in the distribution of several lipid classes, in particular ceramides and phospholipids. However, it remains unclear whether the observed accumulation of lipids is a consequence or contributing factor to the autophagy blockade, or both. As part of mechanistic investigations, we demonstrated that **5j** induces an ER stress response and an accumulation of pro-cathepsin D, suggesting inefficient processing and potentially a lysosomal defect. Further studies are required to gain deeper insights into the underlying mechanism of action in the context of autophagy inhibition. Additionally, exploration of biarylacetamide-affected signaling pathways, as exemplified by those that alter phosphorylation of autophagy-regulatory proteins such as MAP1B, is warranted. Finally, evaluating the derivatives in combination with anticancer agents will help establish their potential efficacy for cancer treatment.

## Materials and methods

### Materials and chemicals

Chemicals, reagents and solvents for synthesis were purchased from commercial sources (ACROS, BLD Pharmatech, Enamine, Fluorochem, Sigma Aldrich, VWR International) and used without further purification.

Dulbecco’s Modified Eagle Medium (DMEM 41,965), Roswell Park Memorial Institute (RPMI) 1640 Medium (11875093), RPMI 1640 GlutaMAX (61870036), fetal bovine serum (FBS, A5256701), penicillin-streptomycin (15140122), DPBS (14190094) were purchased from Gibco. Normocin^TM^ (ant-nr-1) and Zeocin^TM^ (ant-zn-1) were purchased from InvivoGen. 12% Bolt™ Bis-Tris precast gels (NW00125BOX), 4–12% Bolt™ Bis-Tris precast gels (NW04125BOX) and HCS CellMask™ Deep Red Stain (H32721) were purchased from Invitrogen. Pierce BCA Protein Assay Kit (23225), HRP substrate Pierce™ ECL Plus Western Blotting Substrate (32132), SuperSignal™ West Atto Ultimate Sensitivity Substrate (A3855), round-bottom polystyrene test tubes with cell strainer snap cap (Falcon™, 10585801), Permanox 1-well Lab-Tek chamber slides (Nunc 177,410), Reacti-Vials™ (TS-13097) were purchased from Thermo Fisher Scientific. RIPA buffer (ab156034) was purchased from Abcam, Bafilomycin A1 (sc -201,550) from Santa Cruz Biotechnology. DMSO (D2438), doxycycline hyclate (D9891), *N*-ethylmaleimide (E3876), EDTA (99.995%, 431788), HEPES (H3375), Hoechst (B2883), poly-ᴅ-lysine Hydrobromide (P6407), Torin 1 (475991), Thapsigargin (T9033), hippuric acid-(phenyl-^13^C_6_) (93739), L-lysine-^13^C_6_−^15^N_2_ hydrochloride (643459), leucine-5,5,5-D_3_ (486825), D-glucose-^13^C_6_ (389374), glyceryl tri(palmitate-1-^13^C) (425907), and cholic acid-2,2,4,4-D_4_ (614149), ammonium formate (LC-MS grade 70,221), ammonium acetate (LC-MS grade 73,594), ammonium carbonate (379999), Accutase (A6964), L-ascorbic acid (≥99%, A5960), butylated hydroxytoluene (≥99%, 47168) were purchased from Sigma Aldrich. PVDF membrane (Immobilon-P, IPVH00010), acetic acid (100%, LC-MS grade 533,001), ammonia solution (25%, LC-MS grade 533,003), isopropanol (ACS Reagent 109,634), and chloroform (1.07024) were obtained from Merck Millipore. Protease inhibitor cocktail cOmplete^TM^ EDTA-free (11836170001), phosphatase inhibitor PhosSTOP^TM^ (4906845001) and cell proliferation reagent WST-1 (5015944001) were purchased from Roche. Primary antibodies: anti-vinculin (13901S), anti-SQSTM1/p62 (5114S), anti-LC3B (2775S), anti-GABARAP (13733S), anti-ATF4 (11815S), anti-CHOP (2895S), anti-BiP (3177S), anti-phosphorylated eIF2α (9721 L), anti-PDI (3501S) and HRP secondary antibody anti-rabbit IgG (7074S) and anti-mouse IgG (7076S) were purchased from Cell Signaling Technology Europe. Anti-cathepsin D antibody (MA1–26773), which detects both pro-cathepsin D and cathepsin D, was obtained from Thermo Fisher Scientific. Anti-calnexin antibody (610523) was obtained from BD Transduction Laboratories. Caffeine-^13^C_3_ was obtained from Cerilliant Corporation, 18:1-D_7_ lysophosphatidylethanolamine, 18:1-D_7_ lyso-phosphatidylcholine from Avanti Polar Lipids, and octanoyl-L-carnitine-(N-methyl-D_3_), ceramide (d18:1/18:1-[9Z]-^13^C_18_) and L-phenylalanine-13C9 − 15N from Cambridge Isotope Laboratories. Laemmli SDS sample buffer (1610737) and nonfat dry milk (100–04504) were purchased from Bio-rad. 0.2 μm nylon centrifugal filters (82031–356) were acquired from VWR. Black 96-well plates glass-like polymer (P96–1.5P, IBL), transparent 96-well plates (655180), transparent 6-well plates (657160), transparent 12-well plates (655180) were purchased from Greiner Bio One. Ultrapure water (18.2 MΩ) was obtained from an Elga Pure Lab apparatus (Tienen, Belgium). Sterile water (HYCLSH30529.FS) was purchased from Cytiva. Methanol (MeOH), acetonitrile (MeCN), and formic acid (HCOOH, 99%) were purchased with ULC/MS – CC/SFC grade from Biosolve.

### Cell culture

HeLa-Difluo^TM^ hLC3 cells were purchased from InvivoGen and cultured in DMEM supplemented with 10% FBS, penicillin-streptomycin (100 μg/mL), Normocin^TM^ (100 μg/mL) and Zeocin^TM^ (100 µg/mL). For experiments, HeLa-Difluo^TM^ hLC3 cells were resuspended in test media consisting of DMEM supplemented with 10% FBS and penicillin–streptomycin (100 µg/mL). MEFs were generated according to the protocol by Xu (2005) through timed pregnancy in C57BL/6J mice^[55]^. HeLa cells (obtained from ATCC) and MEF cells were cultured in DMEM supplemented with 10% FBS and penicillin/streptomycin (100 μg/mL). LNCaP cells obtained from ATCC were cultured in RPMI 1640 Glutamax, supplemented with 10% FBS and penicillin/streptomycin (100 μg/mL). LDHB-mKeima RPE-1 (generated as described by Engedal *et al*^[16]^) were cultured in RPMI 1640 medium supplemented with 10% FBS and penicillin/streptomycin (100 μg/mL). Cells were maintained at 37°C, 5% CO_2_.

### Cell viability assay

Cell viability was determined with the Cell Proliferation Reagent WST-1 (Roche 5,015,944,001) according to manufacturer’s procedure. Cells were seeded (5 000 cells/well) in 96-well plates, incubated overnight at 37°C, 5% CO_2_. Then, cells were treated for 24 h with biarylacetamide derivatives (1 and 10 µM) or DMSO (0.1%). DMSO-treated cells were used as positive control and lysed cells as negative control. WST-1 (10 µL) was added to each well and plates were incubated for 3 h. The absorbance of the samples was measured using a microplate reader at 440 nm against background control as blank. Three independent experiments and two technical replicates were carried out. Cell viability of control cells (DMSO) was set to 100%.

### Fluorescence microscopy

HeLa-DiFluo^TM^ hLC3 cells were seeded (5 000 cells/well) in black 96-well plates (IBL, glass-like polymer) and incubated overnight. Cells were treated with controls: Torin 1 (1 µM), DMSO (0.1%), and Bafilomycin A1 (161 nM, added for the last 2 h of treatment) or biarylacetamide derivatives (10 µM) for 24 h. After treatment, the cells were fixed with a solution of 4% paraformaldehyde in PBS and stained with HCS CellMask™ Deep Red Stain (0.02 mg/mL) and nuclear marker Hoechst (0.01 mg/mL) and placed on a gyratory shaker for 20 min at room temperature. Fixed cells were washed twice with PBS and stored at 4°C prior imaging with an automated Nikon Ti-E inverted microscope (Nikon Instruments Europe, Amsterdam, Netherlands) equipped with a SPECTRA light engine® solid-state light source (Lumencor, Beaverton, USA) and a Nikon DS-Qi2 digital camera. Nikon NIS-elements software was used to control image acquisition. Per well, 16 images were taken in 4 channels (405, 488, 561 and 640 nm excitation) using a 40x objective (numerical aperture 0.75). The fluorescence channels were separated using standard emission filters and dichroic mirrors. Three technical and biological replicates were performed. Quantification of autophagosomes and autolysosomes was carried out with FIJI image analysis freeware using the Cellblocks.ijm (v09) analysis suite (https://github.com/DeVosLab/CellBlocks), according to a previously published approach^[56,57]^. In brief, the image analysis pipeline relies on the segmentation of nuclei (using the Hoechst channel), cells (using the HCS CellMask channel) and intracellular spots (LC3 spots in two channels) and subsequent feature (number, shape and intensity) extraction for all regions of interest. LC3 spots were specifically enhanced using a Laplacian operator (scale 2) and segmented after setting a user-defined threshold. Only spots residing in the cytoplasm were retained for further analysis. An overlap of more than 1 pixel was considered a spot positive for both channels (GFP and RFP). The spot load was quantified as: spot number per cell ×spot area per cell. All secondary calculations and statistical analysis were performed in RStudio (v4.3.0).

### Western blotting

HeLa cells (50 000 cells/well) and for LNCaP cells (250 000 cells/well) were seeded in 6-well plates and incubated at 37°C and 5% CO_2_ overnight. Thereafter, cells were treated with the desired biarylacetamides (10 µM) for 24 h. Baf A1 (161 nM for HeLa cells, 100 nM for LNCaP cells) was added for the last 2 h of the treatment. Cells were washed with ice-cold DPBS prior to lysis on ice using RIPA lysis buffer containing protease inhibitor cocktail, phosphatase inhibitor and *N*-ethylmaleimide (unless otherwise stated). Lysates were cleared by centrifugation at 14,000 rcf at 4°C for 20 min and the resulting pellet was discarded. Total protein levels were quantified using Pierce BCA Protein Assay Kit and lysates were diluted to equal concentrations before adding laemmli SDS sample buffer containing 5% β-mercaptoethanol and heated to 95°C for 5 min. Equal amounts of sample (16 µg total protein per lane) were loaded into lanes of a 15-well 4–12% or 12% Bolt™ Bis-Tris precast gels and run at a constant voltage of 75 V for 15 min followed by 165 V constant for 45 min. Separated proteins were transferred to PVDF membranes and blocked in 5% skimmed milk in tris-buffered saline with Tween 20 (TBS-T) for 1 h at room temperature. The membranes were incubated with primary antibody overnight at 4°C in TBS-T containing 1% skimmed milk. After washing with TBS-T (3 × 5 min), the membranes were incubated with the HRP-conjugate secondary antibody in TBS containing 1% skimmed milk and 1% SDS for 1 h at room temperature. After washing with TBS-T (3 × 15 min), the membranes were visualized using HRP substrate followed by exposure on Bio-Rad ChemiDoc imaging system. Densitometry analysis was performed using ImageLab (v6.1, Bio-Rad), graphs and statistical analysis were generated in GraphPad Prism (v10). Intensity values were normalized to the loading control intensity values and reported as fold change to the DMSO-treated cells.

### TMT labeling and liquid chromatography-tandem mass spectrometry

HeLa cells were treated in triplicate with either 5j (10 µM, 18 h) dissolved in DMSO, or with DMSO as a control. Cell lysis, digestion and TMT labeling were performed as described^[58]^. In short, cells were lysed in SDS lysis buffer (SDS [5%], Tris-HCl [100 mM, pH 7.6]) at 95°C for 4 min. Protein concentration was determined by Pierce BCA protein assay. 100 µg of protein was used for subsequent reduction with 5 mM TCEP, alkylation with 15 mM iodoacetamide and quenching with 10 mM DTT. Protein lysates were purified by methanol-chloroform precipitation. The resulting protein pellets were resuspended in 40 mM HEPES pH 8.4 and digested with trypsin (10 µg) for 16 h at 37°C. Peptide concentration was measured with Pierce BCA assay. Samples were arranged in a TMTpro 15-plex experiment. 10 µg of each peptide preparation was dissolved in 25 µl of HEPES (40 mM, pH 8.4) and incubated with 40 µg of the appropriate amino reactive TMTpro Label Reagent for 1 h at ambient temperature. Excess TMT label was quenched by addition of 6 μL 5% hydroxylamine and incubation for 15 min at ambient temperature. Labeled peptide samples were then mixed and freeze-dried. The TMT-labeled mixture was subsequently fractionated by reversed phase C18 chromatography using a Zorbax RRHD Eclipse Plus C18 2.1x150 mm column, 1.8 µm particle size, on an Agilent 1200 high performance liquid chromatography system at a flow of 100 μL/min at 30°C. The binary gradient ran from 2% B to 90% B (solvent A: 10 mM ammonium bicarbonate in water pH 8.4, solvent B: 10 mM ammonium bicarbonate in water pH 8.4/acetonitrile 20/80 v/v). Fractions were collected in 12 vials in a “circular fashion” in the time window 16–42 min. In each collection vial the effluent was collected for 20 seconds. After reaching collection vial 12 the collection was continued in vial 1. After collection, the fractions were lyophilized. Peptide fractions were dissolved in water/formic acid (100/0.1 v/v) and subsequently analyzed twice by on‐line C18 nano-HPLC MS/MS with a system consisting of an Ultimate3000nano gradient HPLC system (Thermo Fisher Scientific, Bremen, Germany) and an Exploris480 mass spectrometer (Fisher Scientific). Fractions were injected onto a cartridge precolumn (300 μm × 5 mm, C18 PepMap, 5 µm, 100 A) and eluted via a homemade analytical nano-HPLC column (50 cm × 75 μm; Reprosil-Pur C18-AQ 1.9 µm, 120 A (Dr. Maisch, Ammerbuch, Germany)). The gradient was run from 2% to 40% solvent B (20/80/0.1 water/acetonitrile/formic acid (FA) v/v) in 120 min. The nano-HPLC column was drawn to a tip of ∼5 µm and acted as the electrospray needle of the MS source. The mass spectrometer was operated in data-dependent MS/MS mode for a cycle time of 3 seconds, with a HCD collision energy at 30 V and recording of the MS2 spectrum in the orbitrap, with a quadrupole isolation width of 1.2 Da. In the master scan (MS1) the resolution was 120,000, the scan range 400–1500, at standard AGC target with a maximum fill time of 50 ms. A lock mass correction on the background ion m/z = 445.12 was used. Precursors were dynamically excluded after *n* = 1 with an exclusion duration of 45 s, and with a precursor range of 20 ppm. Charge states 2–5 were included. For MS2 the first mass was set to 110 Da, and the MS2 scan resolution was 45,000 at an AGC target of 200% with a maximum fill time of 60 ms. In a post-analysis process, raw data were first converted to peak lists using Proteome Discoverer version 2.4 (Thermo Fisher Scientific), and submitted to the Uniprot database (*Homo sapiens*, 20596 entries), using Mascot v. 2.2.07 (www.matrixscience.com) for protein identification. Mascot searches were with 10 ppm and 0.02 Da deviation for precursor and fragment mass, respectively, and trypsin as enzyme. Up to two missed cleavages were allowed. Methionine oxidation and acetyl on protein *N*-terminus were set as a variable modification; carbamidomethyl on Cys, TMTpro on *N*-terminus and Lys were set as fixed modification. Protein False Discovery Rate (FDR) was set to 1%. Normalization was on total peptide amount. The values for each TMT channel were normalized to the average of the values for the DMSO samples for the same peptide.

### Lipidomics

#### Sample preparation

MEF cells were seeded at a density of 75,000 cells in collagen-coated Permanox 1-well Lab-Tek chamber slides and maintained at 37°C, 5% CO_2_. After 2 days, cells were treated with **5j** (10 µM, *N* = 12) or DMSO (0.1%, *N* = 12) for 18 h. Extraction blanks were prepared from chamber slides containing culture medium without cells. MEF cells were prepared based on previously described methods^[59]^. In short, cells were snap-frozen in liquid nitrogen and scraped from the carrier with 600 µL quenching solution (80% MeOH and 20% 10 mM CH_3_COONH_4_ [v/v]) at −80°C. Two chamber slides were pooled for liquid–liquid extraction (LLE) with MeOH/H_2_O/CHCl_3_ (3/2/2, v/v), resulting in six replicates. A mixture of 12 internal standards, including 6 polar and 6 apolar compounds, was added. Polar and apolar fractions were transferred and divided for the analysis in ESI (+) and ESI (-). Extracts were dried under a N_2_ stream and reconstituted for analysis.

#### Instrumental analysis

The apolar fraction was analyzed using a 1290 Infinity II LC system (Agilent Technologies, USA) coupled to 6560 drift tube-ion mobility (DTIM)-QToF-HRMS (Agilent Technologies, USA) using Dual Jet Stream ESI source (Agilent Technologies, USA). Separation was performed using an ACQUITY UPLC PREMIER BEH C18 column (150 × 2.1 mm, 1.7 μm, Waters, USA), the mobile phases consisting of 5 mM ammonium acetate in H_2_O/MeCN (7:3, v/v) as MPA and 5 mM ammonium acetate in H_2_O/MeCN/IPA (2:10:88, v/v) as MPB. In the ESI (+) mode, 0.1% (v/v) CH_3_COOH was added to the aqueous fraction of MPA and MPB. Mass calibration was achieved with a continuously infused calibrant solution containing purine and hexakis(1 H,1 H,3 H-tetrafluoropropoxy)phosphazine. Samples were randomized and data were acquired in profile mode using a 2 GHz extended dynamic range. A pooled QC sample was injected six times at regular time intervals (*n* = 6) for quality control. Data-dependent acquisition (DDA) with iterative exclusion was performed on the pooled QC sample during system conditioning to optimize lipidomics analysis.

#### Data processing, statistics and annotation

The raw LC-HRMS data files (.d format) were converted to.mzML format using MSConvert^[60]^ and processed using MS-DIAL (v5.1)^[61]^. The resulting data matrices were cleaned in MS-FLO for further deisotoping and duplicate removal^[62]^. Drift correction was performed using the cubic spline method, and low-quality features were filtered out to ensure data integrity. Following blank subtraction; only features present in at least 80% of the sample groups and an RSD < 30% were retained^[63]^. To address missing values, imputation was performed using random forest. The data were then log-transformed and normalized via probabilistic quotient normalization (PQN), and Pareto-scaled^[64–66]^ to enhance comparability. Subsequently, multivariate statistical analyses were performed to identify the most discriminative lipids. This included: Partial Least Squares-Discriminant Analysis (PLS-DA) with 7-fold cross validation and a binary Random Forest (RF) classifier. The robustness of the PLS-DA model was evaluated using permutation testing (*n* = 1000 random class label shuffles) assessing whether the group separation obtained by the model performed better than chance. Model performance was quantified using R^2^ (goodness-of-fit) and Q^2^ (predictive ability). The RF model was assessed based on the area under the curve (AUC). Key discriminative features were selected if they had a variable importance in projection (VIP) > 1 in PLS-DA and a variable importance measure (VIM) > 0.1 in RF^[67,68]^. For these features, a Shapiro-Wilk test was performed on the intensity values to assess normality. Depending on the normality, either a Mann–Whitney U-test or a Student’s *t*-test was performed^[69]^. A volcano plot was generated to visualize differential features and only features with *p*-value < 0.05 and log_2_ fold change (FC) > 0.5 or < 0.5 were annotated. Metabolites were annotated using MS/MS libraries (LipidBlast, MoNA, NIST) and rule-based tool: LipidMatch^[70]^. Annotation confidence was further enhanced through manual spectral evaluation and cross-referencing with an in-house library.

### Statistical analysis

Quantitative data shown in the figures represent the mean ± standard deviation (SD, unless otherwise stated) from at least three independent experiments. Statistical analyses were performed using two-sided Student’s *t*-test, one- or two-way ANOVA followed by multiple comparison using Dunnett’s or Tukey’s test. Differences at *p < 0.05* were considered statistically significant.

## Abbreviation

Baf A1: bafilomycin A1
DMSO: dimethyl sulfoxide
ER: endoplasmic reticulum
ESI: electrospray ionization
FASN: fatty acid synthase
GABARAP: gamma-aminobutyric acid receptor-associated protein
HTS: high-throughput screening
LC3: microtubule-associated protein 1A/1B light chain 3
LC-MS/MS: liquid chromatography tandem mass spectrometry
LDHB: lactate dehydrogenase B
MEFs: mouse embryonic fibroblasts
PCA: principal component analysis
PCs: phosphatidylcholines
PEs: phosphatidylethanolamines
PGs: phosphatidylglycerols
PI: phosphatidylinositol
PIPs: phosphatidylinositol phosphates
SQSTM1/p62: sequestosome-1
TMT: tandem mass tag
UPR: unfolded protein response

## Supplementary Material

Supplemental Material

## Data Availability

All data supporting the findings of this study are available within the paper, its Supplementary Information and from Zenodo public repository https://doi.org/10.5281/zenodo.15631606.
