# Supplementary material for: Biarylacetamides: a novel class of late-stage autophagy inhibitors
Source: Autophagy Rep. 2025 Aug 7;4(1):2541597. doi: 10.1080/27694127.2025.2541597 (PMC12439681; doi:10.1080/27694127.2025.2541597)
Supplement: Supplemental Material [file KAUO_A_2541597_SM3494.docx]

# Supplementary information

**Biarylacetamides: a novel class of late-stage autophagy inhibitors**

Mélissa Lallier ^1,2^, Rani Robeyns ^3^, Freke Mertens ^2,4^, Angela Sisto ^4^, Guido R.Y. De Meyer ^2,8^, Koen Augustyns ^1,8^, Maya Berg ^8^, Winnok H. De Vos ^5-7^, Vincent Timmerman ^4,7^, George M.C. Janssen ^9^, Peter van Veelen ^9^, Alexander L.N. van Nuijs ^3^, Nikolai Engedal ^10^, Wim Martinet ^2^, Pieter Van der Veken ^1^

**Affiliations**

1. Laboratory of Medicinal Chemistry, Department of Pharmaceutical Sciences, University of Antwerp, Antwerp, Belgium
2. Laboratory of Physiopharmacology, Department of Pharmaceutical Sciences, University of Antwerp, Antwerp, Belgium
3. Toxicological Centre, Department of Pharmaceutical Sciences, University of Antwerp, Antwerp, Belgium
4. Peripheral Neuropathy Research Group, Department of Biomedical Sciences, University of Antwerp, Antwerpen, Belgium
5. Laboratory of Cell Biology and Histology, Department of Veterinary Sciences, University of Antwerp, Antwerp, Belgium
6. Antwerp Centre for Advanced Microscopy, University of Antwerp, Antwerp, Belgium
7. µNEURO Centre of Excellence, University of Antwerp, Antwerp, Belgium
8. Infla-Med Centre of Excellence, University of Antwerp, Antwerp, Belgium
9. Center for Proteomics and Metabolomics, Leiden University Medical Center (LUMC), Leiden, Netherlands
10. Autophagy in Cancer Lab, Department of Tumor Biology, Oslo University Hospital, Oslo, Norway

## Additional figures


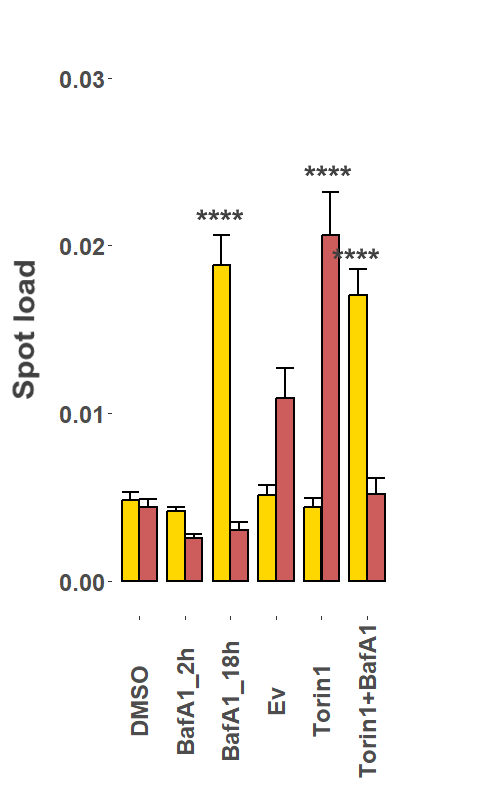


**Figure S1**. Baf A1 potently inhibits autophagy after 18 h treatment.

HeLa cells stably expressing the RFP-GFP-LC3 reporter were treated with Baf A1 (161 nM, 2 h), Baf A1 (50 nM, 18 h), Everolimus (Ev, 10 µM, 18 h), Torin 1 (1 µM, 18 h) and Torin 1 + Baf A1 (Torin 1, 1 µM, 18 h, Baf A1, 161 nM, 2 h). **A.** The bar graph represents spot load ([puncta number per cell x puncta area mean per cell]/cell area) of autophagosomes (yellow) and autolysosomes (red) per cell. The mean ± SEM of three independent experiments is plotted, *p*-values were calculated by Tukey’s HSD test (****, *p* < 0.0001).


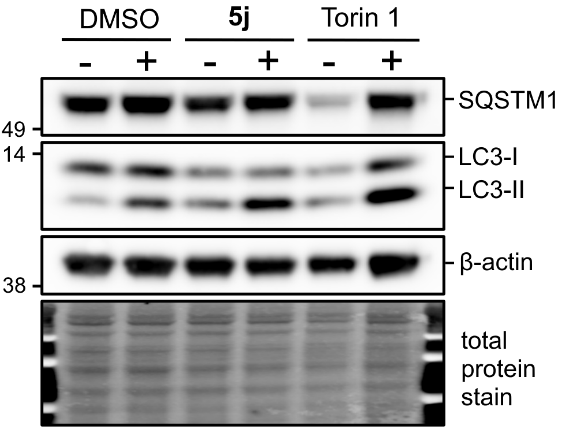


**Figure S2**. Compound **5j** inhibits autophagy in MEFs.

Western blot analysis of SQSTM1/p62, LC3-I, LC3-II and β-actin. Mouse embryonic fibroblasts (MEFs) treated with DMSO (0.1%), **5j** (10 µM) or Torin 1 (1 µM) for 18 h in the presence or absence of Baf A1 (100 nM, last 2 h of treatment).


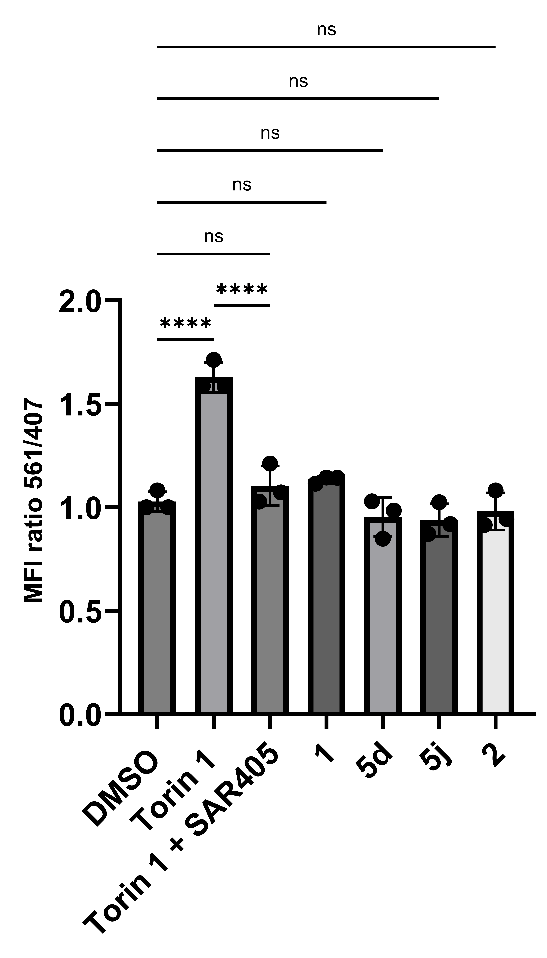


**Figure S3.** The biarylacetamide derivatives do not stimulate bulk autophagic cargo flux.

hTERT RPE-1 LDHB-mKeima cells were treated with positive (Torin 1, 50 nM) and negative (Torin 1 [50 nM] + SAR405 [1 µM]) controls or with the indicated biarylacetamides at 10 µM for 24 h. The bar graph represents the mean ± SD of three independent experiments, *p*-values were calculated by Dunnett’s test (ns, non significant; **, *p* < 0.01; ****, *p* < 0.0001).


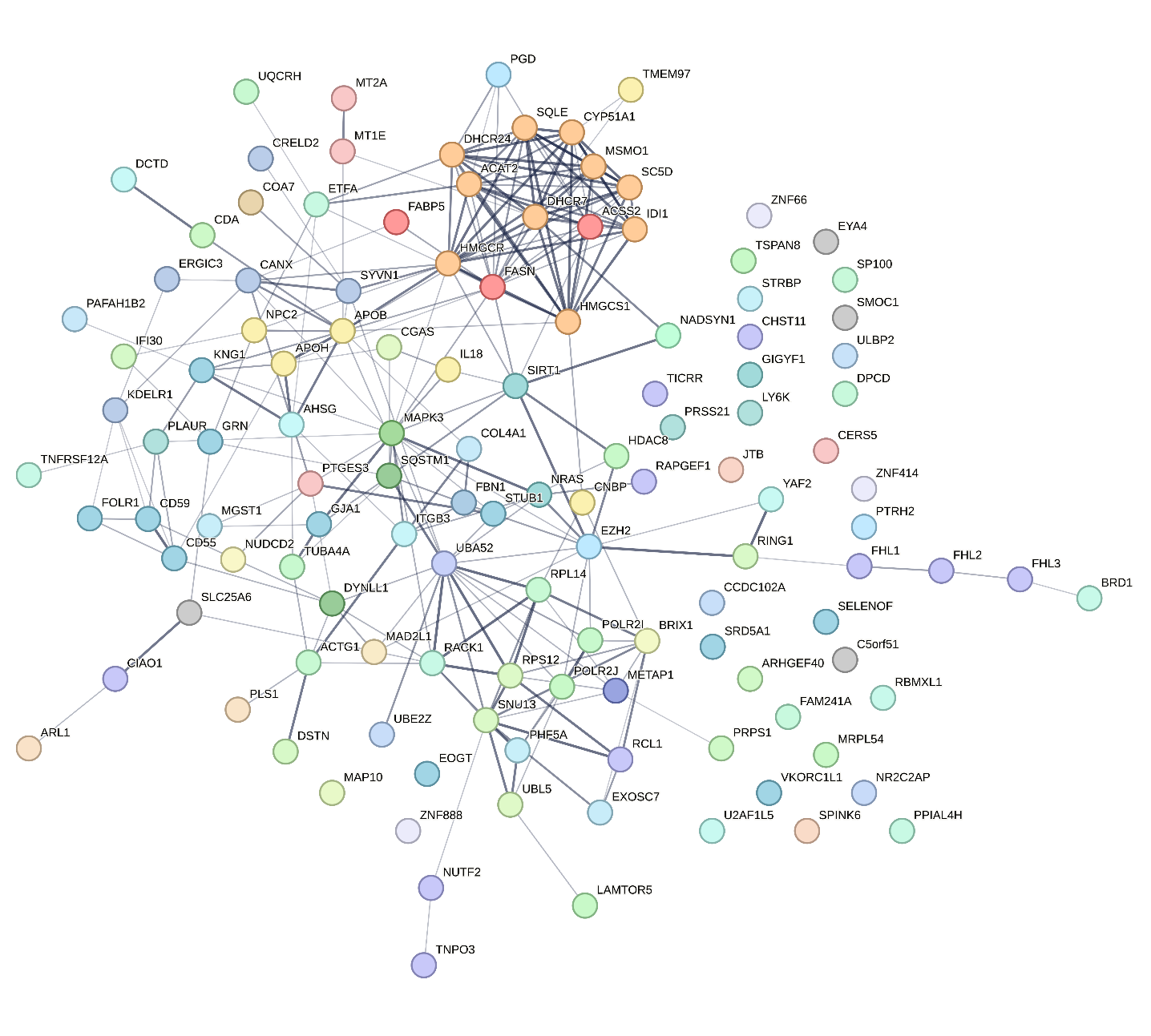


**Figure S4.** Protein-protein interactions network after **5j** treatment in HeLa cells.

The protein-protein interactions network of the significantly upregulated proteins following **5j** treatment (10 µM, 18 h) in HeLa cells was generated by STRING (v12, <https://string-db.org/>).

**
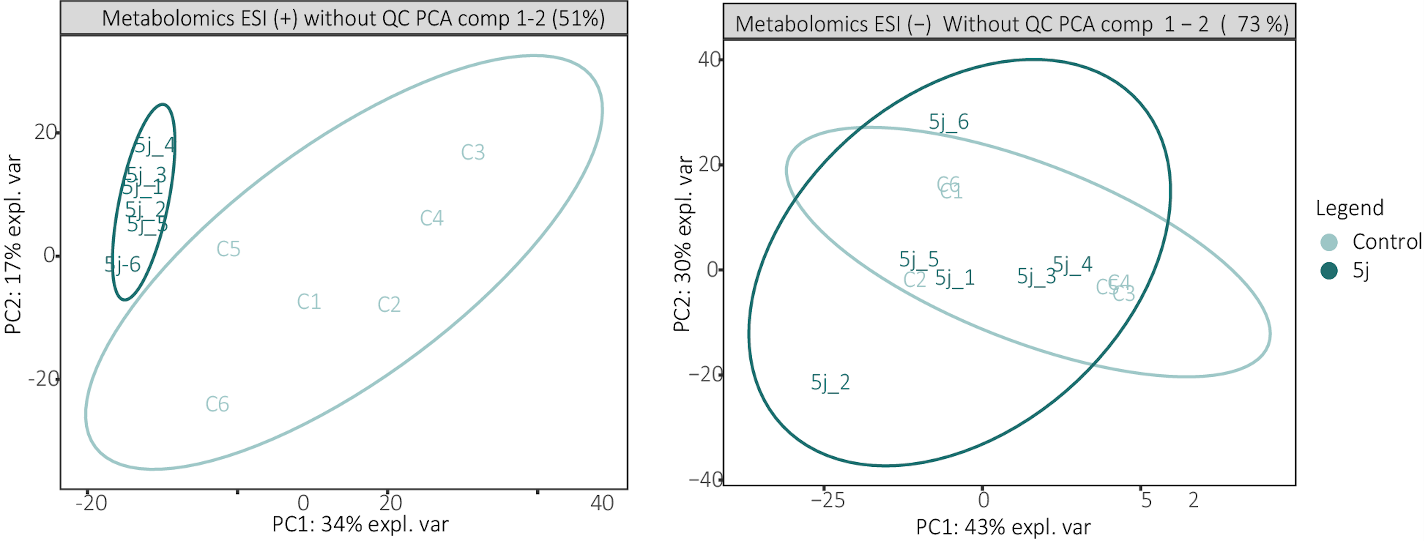
Figure S5.** Metabolomics PCA plots after **5j** treatment.

Metabolomics principal component analysis (PCA) plots in positive and negative ESI mode showing the clustering patterns of metabolites profiles in apolar fractions of MEF cells treated with compound **5j** (10 µM) and DMSO for 18 h.


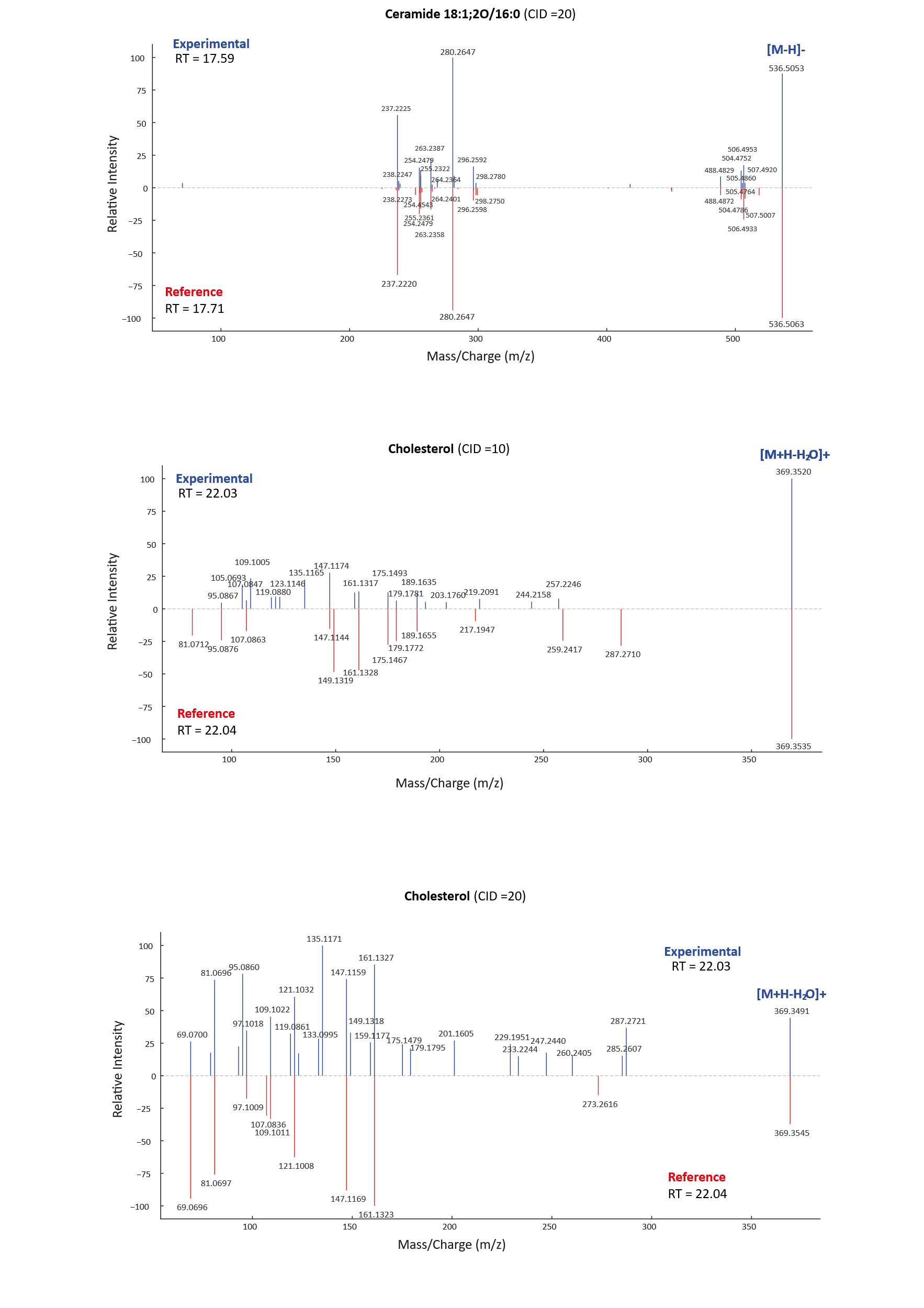


**Figure S6.** Lipid identification validation for Ceramide 18:1;2O/16:0 and cholesterol.

Mirror plots of MS/MS fragmentation pattern of Ceramide 18:1;2O/16:0 with collision energy of 20 and cholesterol with collision energy of 10 and 20 from extracted samples (in blue) and reference standards (in red).

## Additional methods

### LDHB-mKeima assay (flow cytometry)

The procedure was as described by Engedal et al. [1]. hTERT RPE-1 LDHB-mKeima cells were seeded in 12-well plates (40 000 cells/well) and pretreated with doxycycline hyclate for 48 h. The cells were washed with DPBS and treated with controls: DMSO, Torin 1 (50 nM), Torin 1 (50 nM) co-treated with SAR405 (1 µM), Torin 1 co-treated with Baf A1 (100 nM, Baf A1 was added for the last 2 hours of treatment), and the biarylacetamides derivatives (**1**, **2**, **5d**, **5j**) at 10 µM for 24 h. The cells were harvested with Trypsin-EDTA and centrifuged at 4°C for 4 minutes at 400 x g. The cell pellets were resuspended in FACS buffer (HEPES (15 mM), EDTA (1 mM), FBS (1%) in PBS, pH 7.3 – 7.5). The cells were filtered through a 35 µm mesh filter cap into a 5 mL flow tube before acquisition on a NovoCyte Quanteon Flow Cytometer (Agilent, USA). mKeima was excited by 405 nm and 561 lasers with a 615/20 bandpass filter. The ratiometric analysis was performed using FlowJo™ software (BD Biosciences, USA). The gating strategy described in [1] was applied. Then, the derived ratio of 561/405 nm signal intensity per cell was calculated, and the median value of these ratios were normalized to the median value ratio obtained for DMSO.

[1] Engedal N, Sønstevold T, Beese CJ, et al. Measuring Autophagic Cargo Flux with Keima-Based Probes. Methods in Molecular Biology. 2022. p. 99–115.

## Additional data


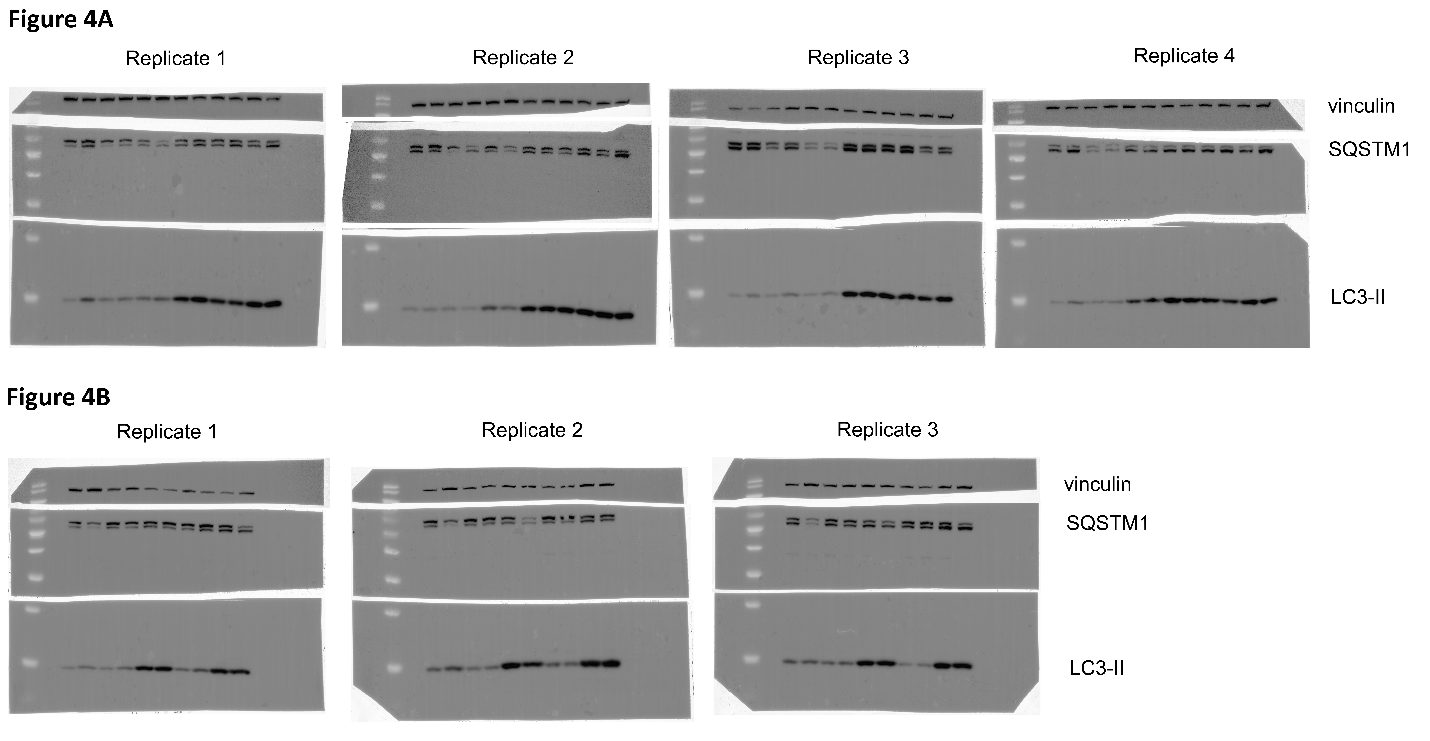


**Data S1.** Uncropped western blots membranes presented in Figure 4A and 4B.


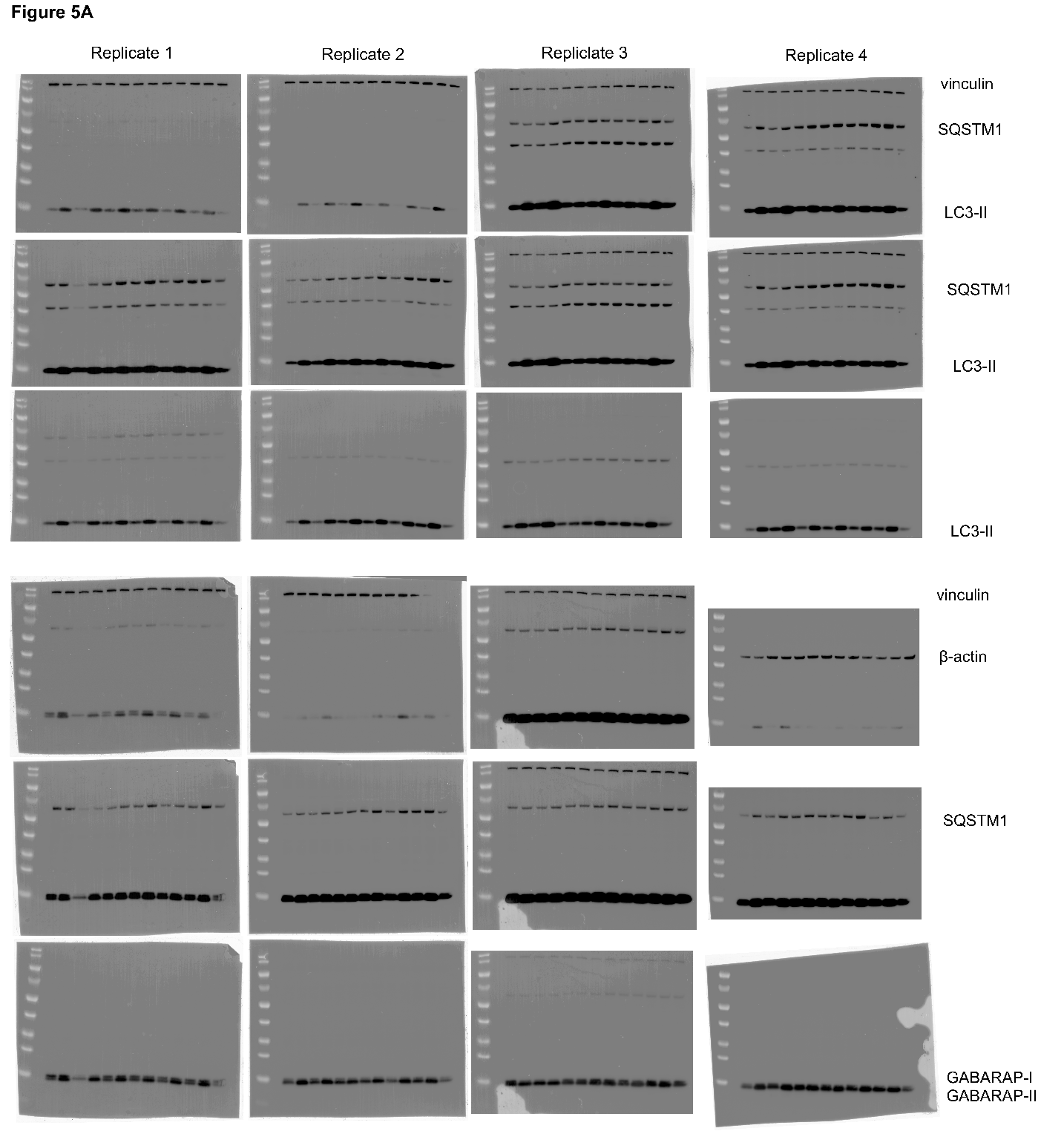


**Data S2.** Uncropped western blots membranes presented in Figure 5A.


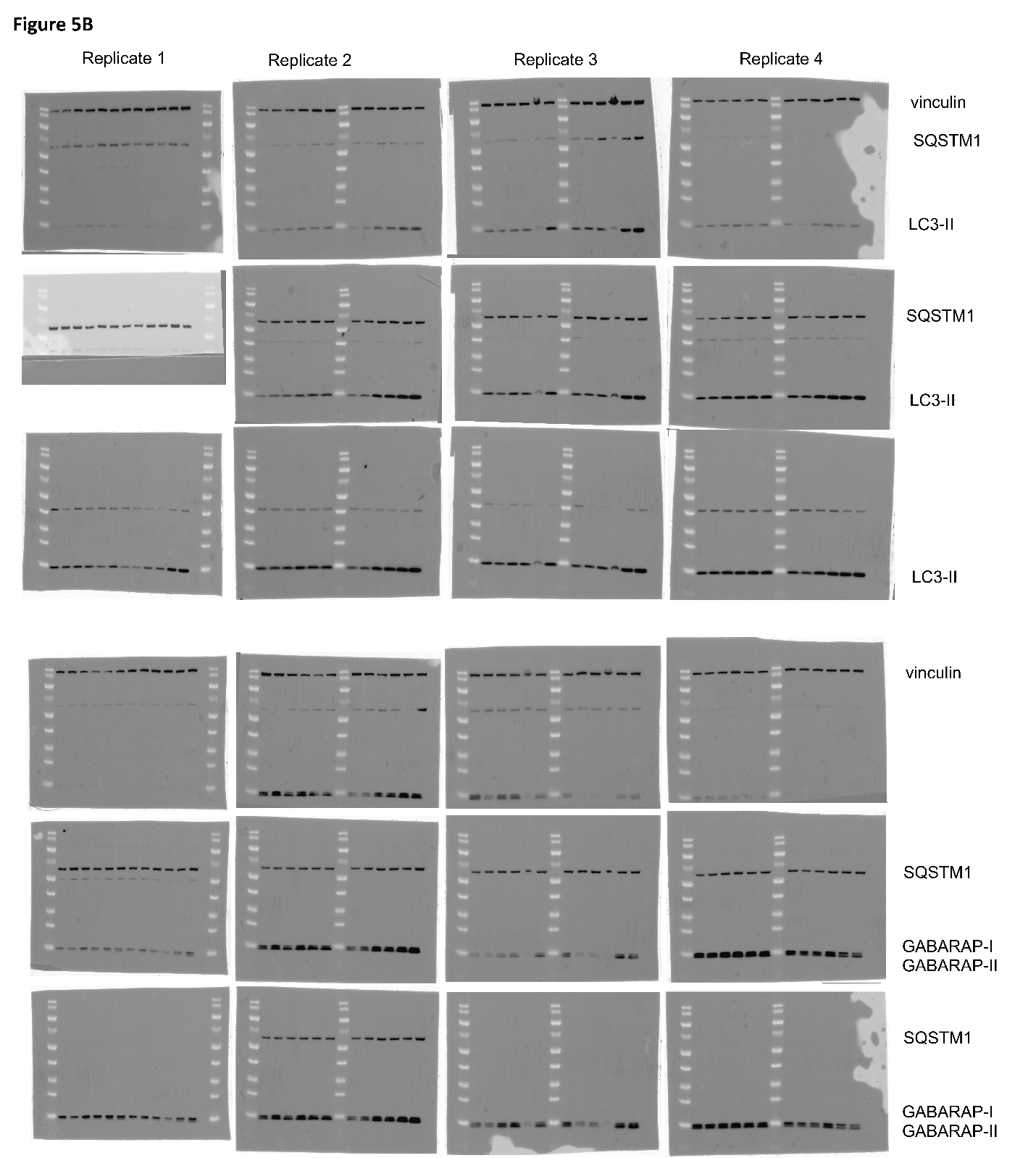


**Data S3.** Uncropped western blots membranes presented in Figure 5B**.**

**
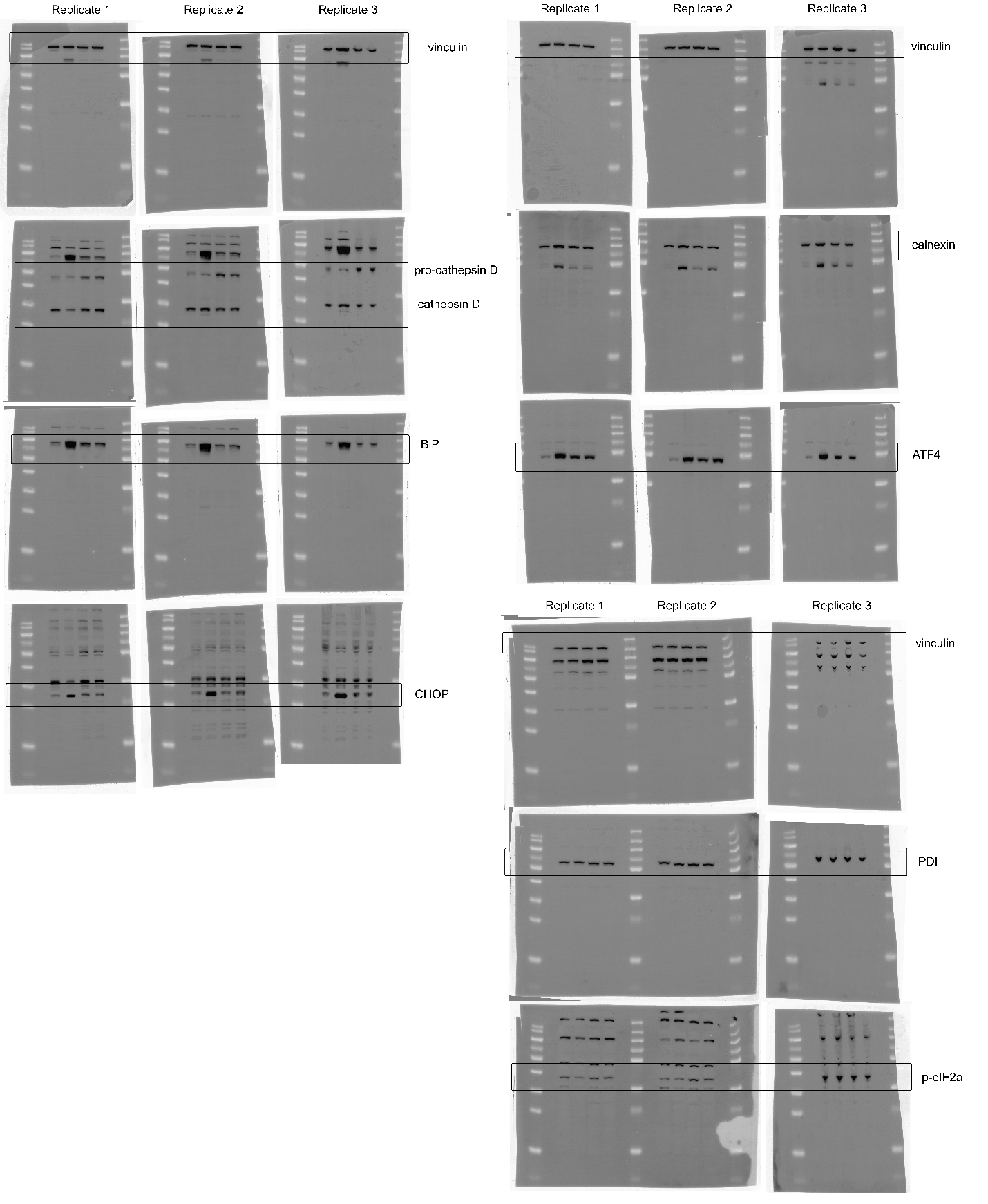
**

**Data S4.** Uncropped western blots membranes presented in Figure 8**.**

## Chemistry

### Synthesis and characterization data for finals and intermediates

#### General Remarks

Reaction progress was monitored by thin-layer chromatography (TLC) carried out on normal phase silica gel (60 F_254_) aluminium plates with UV light (*λ*_max_ = 254/360 nm) detection. TLC plates were stained with ninhydrin solution if necessary. Compounds were purified on Biotage® Isolera One flash system, equipped with internal variable dual-wavelength diode array detector (200–400 nm), using CHROMABOND cartridges (4–25 g) when necessary. Characterization of all compounds was carried out with ^1^H and ^13^C NMR and mass spectrometry. ^1^H NMR and ^13^C NMR spectra were recorded on 400 MHz Bruker Avance III DRX-400 spectrometer.^1^H and ^13^C NMR chemical shifts are given as *δ*_H_ and *δ*_C_, respectively, in parts per million (ppm). Spectra were analyzed using MestNova software.

#### General procedure A for the synthesis of biaryl acetic acid

To a glass vial, the appropriate boronic acid (1.5 eq) and the appropriate aryl bromide (1.0 eq) were dissolved in an aqueous solution of potassium carbonate (4 M) and acetonitrile (1:1). Once the mixture was degassed under an argon flow, Pd(tetrakis)triphenylphosphine (5 mol%) was added and the vial was sealed and heated to 90 °C for 1 to 3 days. The reaction mixture was cooled to room temperature and diluted with dichloromethane (20 mL) and water (10 mL). The phases were separated. The organic layer was extracted with an aqueous solution of NaOH (1 M, 3 x 10 mL). The aqueous layers were combined and acidified with a 4 M HCl aqueous solution. The white suspension was extracted with dichloromethane (3 x 10 mL), the organic phases were combined, washed with brine, dried over anhydrous sodium sulphate and filtered. The solvent was evaporated under reduced pressure. The crude was purified by normal-phase flash chromatography when necessary.

#### General procedure B for the synthesis of biarylacetamide derivatives

The appropriate amine (1.0 eq) and acid (1.0 eq) were dissolved in anhydrous DMF (0.1 M). To this solution, HATU (1.1 eq) was added followed by anhydrous *N,N-*diisopropylethylamine (3 eq). The reaction was stirred at room temperature for 18 hours. The reaction mixture was diluted with dichloromethane and water. The phases were separated, the organic layer was washed with brine, dried over anhydrous sodium sulphate and the solvents were removed under reduced pressure. The crude was purified as indicated below to yield pure compound).

***N*-(4-methoxybenzyl)-2-phenylacetamide (3a).**

Prepared according to general procedure B using phenylacetic acid (100 mg, 0.734 mmol) and 4‑methoxybenzylamine (96 µL, 0.734 mmol). The crude was recrystallized from acetonitrile yielding the desired molecule as white crystals (139 mg, 0.544 mmol, 74% yield). ^1^H NMR (400 MHz, CDCl3) δ 7.38 – 7.31 (m, 2H), 7.31 – 7.23 (m, 3H), 7.11 (d, *J* = 8.7 Hz, 2H), 6.82 (d, *J* = 8.7 Hz, 2H), 5.63 (s, 1H), 4.34 (d, J = 5.6 Hz, 2H), 3.79 (s, 3H), 3.62 (s, 2H). ^13^C NMR (101 MHz, CDCl3) δ 170.8, 159.0, 134.8, 130.2, 129.5, 129.1, 128.9, 127.4, 114.0, 55.3, 43.9, 43.1. MS (ESI^+^) *m/z* 256.2 [M+H]^+^.

***N*-(pyrdin-4-ylmethyl)-2-phenylacetamide (3b).**

Prepared according to general procedure B using phenylacetic acid (100 mg, 0.734 mmol) and 4‑(aminomethyl)pyridine (75 µL, 0.734 mmol). The crude was recrystallized from acetonitrile yielding a crystalline white solid (142 mg, 0.596 mmol, 81% yield). ^1^H NMR (400 MHz, CDCl3) δ 8.48 (d, *J* = 5.4 Hz, 2H), 7.42 – 7.34 (m, 2H), 7.34 – 7.27 (m, 3H), 7.09 (d, *J* = 5.4 Hz, 2H), 6.03 (s, 1H), 4.41 (d, *J* = 6.1 Hz, 2H), 3.66 (s, 2H). ^13^C NMR (101 MHz, CDCl3) δ 171.3, 149.4, 148.0, 134.5, 129.4, 129.2, 127.7, 122.2, 43.7, 42.3. MS (ESI^+^) *m/z* 227.2 [M+H]^+^.

***N*-(thiophen-2-ylmethyl)-2-phenylacetamide (3c).**

Prepared according to general procedure B using phenylacetic acid (150 mg, 1.10 mmol) and thiophen-2-ylmethanamine (113 µL, 1.10 mmol). The crude was recrystallized from acetonitrile yielding white crystals (163 mg, 0.705 mmol, 64% yield). ^1^H NMR (400 MHz, CDCl_3_) δ 7.32 – 7.25 (m, 2H), 7.25 – 7.16 (m, 3H), 7.12 (dd, *J* = 5.1, 1.3 Hz, 1H), 6.84 (dd, *J* = 5.1, 3.5 Hz, 1H), 6.82 – 6.78 (m, 1H), 5.68 (s, 1H), 4.51 (dd, *J* = 5.7, 0.9 Hz, 2H), 3.54 (s, 2H). ^13^C NMR (101 MHz, CDCl_3_) δ 170.6, 140.9, 134.6, 129.5, 129.1, 127.5, 126.9, 125.7, 125.1, 43.7, 38.5. MS (ESI^+^) *m/z* 232.2 [M+H]^+^.

**2-([1,1’-biphenyl]-4-yl)*-N*-(4-(dimethylamino)benzyl)acetamide (4a).**

Prepared according to general procedure B using 4-biphenylacetic acid (100 mg, 0.471 mmol) and 4-dimethylaminobenzylamine dihydrochloride (105 mg, 0.471 mmol). The crude was purified by normal-phase flash chromatography (7% methanol in dichloromethane) and subsequently recrystallized from acetonitrile yielding white crystals (148 mg, 0.408 mmol, 87% yield). ^1^H NMR (400 MHz, CDCl_3_) δ 7.62 – 7.53 (m, 4H), 7.44 (t, *J* = 7.5 Hz, 2H), 7.40 – 7.30 (m, 3H), 7.10 (d, *J* = 8.7 Hz, 2H), 6.69 (s, 2H), 5.65 (s, 1H), 4.34 (d, *J* = 5.5 Hz, 2H), 3.64 (s, 2H), 2.93 (s, 6H). ^13^C NMR (101 MHz, CDCl_3_) δ 170.6, 149.6, 140.6, 140.2, 133.9, 129.9, 128.9, 128.8, 127.7, 127.4, 127.1, 43.5, 43.4, 40.7. MS (ESI^+^) *m/z* 345.1 [M+H]^+^.

**2-([1,1’-biphenyl]-4-yl)*-N*-(pyridine-4-ylmethyl)acetamide (4b)**

Prepared according to general procedure B using 4-biphenylacetic acid (150 mg, 0.707 mmol) and 4‑(aminomethyl)pyridine (71 µL, 0.707 mmol). The crude was purified by normal-phase flash chromatography (7% methanol in dichloromethane) and subsequently recrystallized from acetonitrile yielding white crystals (148 mg, 0.626 mmol, 89% yield). ^1^H NMR (400 MHz, CDCl_3_) δ 8.51 (d, *J* = 6.2 Hz, 2H), 7.65 – 7.54 (m, 4H), 7.48 – 7.41 (m, 2H), 7.41 – 7.31 (m, 3H), 7.12 (d, *J* = 6.1 Hz, 2H), 6.03 (s, 1H), 4.44 (d, *J* = 6.1 Hz, 2H), 3.71 (s, 2H). ^13^C NMR (101 MHz, CDCl_3_) δ 171.2, 149.5, 147.9, 140.6, 140.3, 133.4, 129.9, 128.9, 127.9, 127.6, 127.0, 122.2, 43.3, 42.4. MS (ESI^+^) *m/z* 303.2 [M+H]^+^.

**2-([1,1’-biphenyl]-4-yl)*-N*-(4-chlorobenzyl)acetamide (4c)**

Prepared according to general procedure B using 4-biphenylacetic acid (150 mg, 0.707 mmol) and 4‑chlorobenzylamine (86 µL, 0.707 mmol). The crude was recrystallized from acetonitrile yielding white crystals (184 mg, 0.520 mmol, 74% yield). ^1^H NMR (400 MHz, DMSO-*d_6_*) δ 8.63 (t, *J* = 6.0 Hz, 1H), 7.69 – 7.56 (m, 4H), 7.46 (t, *J* = 7.7 Hz, 2H), 7.42 – 7.31 (m, 5H), 7.27 (d, *J*= 8.5 Hz, 2H), 4.27 (d, *J* = 5.9 Hz, 2H), 3.52 (s, 2H). ^13^C NMR (101 MHz, DMSO-*d_6_*) δ 170.6, 140.5, 139.0, 138.8, 136.0, 131.8, 130.1, 129.6, 129.4, 128.7, 127.8, 127.0, 42.4, 42.0. MS (ESI^+^) *m/z* 336.2 [M+H]^+^.

**2-([1,1’-biphenyl]-4-yl)*-N*-(4-methoxybenzyl)acetamide (4d).**

Prepared according to general procedure B using 4-biphenylacetic acid (150 mg, 0.707 mmol) and 4‑methoxybenzylamine (92 µL, 0.707 mmol). The crude was recrystallized from acetonitrile yielding white crystals (201 mg, 0.606 mmol, 86% yield). ^1^H NMR (400 MHz, CDCl_3_) δ 7.69 – 7.48 (m, 4H), 7.44 (t, *J* = 7.6 Hz, 2H), 7.40 – 7.31 (m, 3H), 7.14 (d, *J* = 8.4 Hz, 2H), 6.83 (d, *J* = 8.5 Hz, 2H), 5.71 (s, 1H), 4.37 (d, *J* = 5.1 Hz, 2H), 3.78 (s, 3H), 3.66 (s, 2H). ^13^C NMR (101 MHz, CDCl_3_) δ 170.8, 159.0, 140.5, 140.3, 133.7, 130.1, 129.9, 129.0, 128.8, 127.8, 127.5, 127.0, 114.1, 55.3, 43.5, 43.2. MS (ESI^+^) *m/z* 332.3 [M+H]^+^.

**2-([1,1’-biphenyl]-4-yl)*-N*-(4-cyanobenzyl)acetamide (4e).**

Prepared according to general procedure B using 4-biphenylacetic acid (150 mg, 0.707 mmol) and 4‑(aminomethyl)benzonitrile (92 µL, 0.707 mmol). The crude was recrystallized from acetonitrile yielding white crystals (181 mg, 0.555 mmol, 78% yield). ^1^H NMR (400 MHz, CDCl_3_) δ 7.58 – 7.46 (m, 6H), 7.42 – 7.34 (m, 2H), 7.33 – 7.25 (m, 3H), 7.23 (dd, *J* = 7.9, 0.7 Hz, 2H), 5.84 (s, 1H), 4.40 (d, *J* = 6.1 Hz, 2H), 3.62 (s, 2H). ^13^C NMR (101 MHz, CDCl_3_) δ 171.2, 143.8, 140.6, 140.3, 133.4, 132.5, 129.9, 128.9, 128.0, 127.9, 127.6, 127.0, 118.7, 111.3, 43.4, 43.1. MS (ESI^+^) *m/z* 327.3 [M+H]^+^.

**Methyl 4-((2-([1,1'-biphenyl]-4-yl)acetamido)methyl)benzoate (4f).**

Prepared according to general procedure B using 4-biphenylacetic acid (150 mg, 0.707 mmol) and methyl 4-(aminomethyl)benzoate hydrochloride (143 mg, 0.707 mmol). The crude was recrystallized from acetonitrile yielding white crystals (172 mg, 0.479 mmol, 68% yield). ^1^H NMR (400 MHz, DMSO) δ 8.70 (t, *J* = 6.0 Hz, 1H), 7.91 (d, *J* = 8.3 Hz, 2H), 7.78 – 7.53 (m, 4H), 7.46 (dd, *J* = 8.4, 6.9 Hz, 2H), 7.42 – 7.31 (m, 5H), 4.36 (d, *J* = 5.9 Hz, 2H), 3.83 (s, 3H), 3.54 (s, 2H). ^13^C NMR (101 MHz, DMSO) δ 170.74, 166.55, 145.69, 140.46, 138.82, 136.02, 130.09, 129.70, 129.39, 128.58, 127.82, 127.77, 127.03, 52.55, 42.44, 42.41. MS (ESI^+^) *m/z* 360.3 [M+H]^+^.

**2-([1,1’-biphenyl]-4-yl)*-N*-benzylacetamide (4g).**

Prepared according to general procedure B using 4-biphenylacetic acid (100 mg, 0.471 mmol) and benzylamine (52 µL, 0.471 mmol). The crude was recrystallized from acetonitrile yielding white crystals (114 mg, 0.378 mmol, 80% yield). ^1^H NMR (400 MHz, CDCl_3_) δ 7.51 (d, *J* = 8.3 Hz, 4H), 7.37 (t, *J* = 7.5 Hz, 2H), 7.31 – 7.25 (m, 3H), 7.23 (d, *J* = 7.4 Hz, 2H), 7.21 – 7.15 (m, 2H), 7.13 (d, *J* = 6.7 Hz, 2H), 5.68 (s, 1H), 4.37 (d, *J* = 5.8 Hz, 2H), 3.60 (s, 2H). ^13^C NMR (101 MHz, CDCl_3_) δ 170.80, 140.52, 140.38, 138.12, 133.71, 129.92, 128.85, 128.71, 127.80, 127.57, 127.50, 127.46, 127.05, 43.66, 43.48. MS (ESI^+^) *m/z* 302.2 [M+H]^+^.

**2-([1,1’-biphenyl]-4-yl)*-N*-(4-((dimethylamino)methyl)benzyl)acetamide (1).**

Prepared according to general procedure B using 4-biphenylacetic acid (150 mg, 0.707 mmol) and 1-(4-(aminomethyl)phenyl)-*N*,*N*-dimethylmethanamine hydrochloride (142 mg, 0.707 mmol). The crude was purified by normal-phase flash chromatography (8% methanol in dichloromethane) yielding an off-white powder (207 mg, 0.577 mmol, 82% yield). ^1^H NMR (400 MHz, CDCl_3_) δ 7.65 – 7.49 (m, 4H), 7.49 – 7.39 (m, 2H), 7.40 – 7.28 (m, 3H), 7.24 (d, *J* = 8.1 Hz, 2H), 7.15 (d, *J* = 8.2 Hz, 2H), 5.73 (s, 1H), 4.43 (d, *J* = 5.8 Hz, 2H), 3.67 (s, 2H), 3.38 (s, 2H), 2.21 (s, 6H). ^13^C NMR (101 MHz, CDCl_3_) δ 170.78, 140.51, 140.36, 138.20, 136.87, 133.72, 129.43, 128.84, 127.78, 127.48, 127.45, 127.05, 63.99, 45.37, 43.49, 43.41. HRMS (ESI^+^) *m/z* [M+H]^+^ calculated for C_24_H_26_N_2_O_1_ 239.2118, found 359.2114.

**2-(3’-chloro-[1,1’-biphenyl]-4-yl)*-N*-(4-((dimethylamino)methyl)benzyl)acetamide (5a).**

Prepared according to general procedure B using 2-(3'-chloro-[1,1'-biphenyl]-4-yl)acetic acid (150 mg, 0.608 mmol) and 1-(4-(aminomethyl)phenyl)-*N*,*N*-dimethylmethanamine hydrochloride (122 mg, 0.608 mmol). The crude was purified by normal-phase flash chromatography (8% methanol in dichloromethane) and subsequently recrystallized from acetonitrile yielding a white solid (168 mg, 0.410 mmol, 68% yield). ^1^H NMR (400 MHz, CDCl_3_) δ 7.55 (d, *J* = 7.6 Hz, 3H), 7.46 (d, *J* = 6.7 Hz, 1H), 7.37 (q, *J* = 7.1 Hz, 4H), 7.27 (d, *J* = 7.0 Hz, 2H), 7.18 (d, *J* = 7.7 Hz, 2H), 5.77 (s, 1H), 4.44 (d, *J* = 5.8 Hz, 2H), 3.67 (s, 2H), 3.43 (s, 2H), 2.25 (s, 6H). ^13^C NMR (101 MHz, CDCl_3_) δ 170.59, 142.37, 138.92, 137.07, 134.72, 134.46, 130.08, 130.02, 129.55, 127.72, 127.57, 127.44, 127.18, 125.19, 63.82, 45.20, 43.44, 43.42. MS (ESI^+^) *m/z* 393.3 [M+H]^+^.

**2-(3’,4’-dichloro-[1,1’-biphenyl]-4-yl)*-N*-(4-((dimethylamino)methyl)benzyl)acetamide (5b).**

Prepared according to general procedure B using 2-(3',4’-dichloro-[1,1'-biphenyl]-4-yl)acetic acid (150 mg, 0.534 mmol) and 1-(4-(aminomethyl)phenyl)-*N*,*N*-dimethylmethanamine hydrochloride (107 mg, 0.534 mmol). The crude was purified by normal-phase flash chromatography (8% methanol in dichloromethane) and subsequently recrystallized from acetonitrile yielding white crystals (177 mg, 0.297 mmol, 56% yield). ^1^H NMR (400 MHz, CDCl_3_) δ 7.65 (d, *J* = 2.1 Hz, 1H), 7.51 (dd, *J* = 8.3, 6.7 Hz, 3H), 7.40 (d, *J* = 2.2 Hz, 1H), 7.36 (d, *J* = 8.2 Hz, 2H), 7.26 (d, *J* = 8.0 Hz, 2H), 7.17 (d, *J* = 8.1 Hz, 2H), 5.75 (s, 1H), 4.43 (d, *J* = 5.8 Hz, 2H), 3.66 (s, 2H), 3.43 (s, 2H), 2.25 (s, 6H). ^13^C NMR (101 MHz, CDCl_3_) δ 170.59, 140.68, 137.99, 137.19, 134.89, 133.04, 131.72, 130.88, 130.23, 129.69, 128.98, 127.72, 127.67, 126.38, 63.91, 45.29, 43.57, 43.52. MS (ESI^+^) *m/z* 427.3 [M+H]^+^.

**2-([1,1’-biphenyl]-4-yl)*-N*-(4-(2-(dimethylamino)ethoxyl)benzyl)acetamide (4h).**

Prepared according to general procedure B using 4-biphenylacetic acid (150 mg, 0.707 mmol) and 2-(4-(aminomethyl)phenoxy)-*N*,*N*-dimethylethanamine (134 µL, 0.534 mmol). The crude was recrystallized from acetonitrile yielding white powder (212 mg, 0.545 mmol, 77% yield). ^1^H NMR (400 MHz, CDCl_3_) δ 7.64 – 7.48 (m, 4H), 7.44 (t, *J* = 7.6 Hz, 2H), 7.39 – 7.28 (m, 3H), 7.12 (d, *J* = 8.5 Hz, 2H), 6.85 (d, *J* = 8.6 Hz, 2H), 5.69 (s, 1H), 4.36 (d, *J* = 5.7 Hz, 2H), 4.07 (t, *J* = 5.6 Hz, 2H), 3.65 (s, 2H), 2.77 (t, *J* = 5.6 Hz, 2H), 2.37 (s, 6H). ^13^C NMR (101 MHz, CDCl_3_) δ 170.69, 158.11, 140.52, 140.32, 133.76, 130.42, 129.90, 128.94, 128.84, 127.75, 127.45, 127.04, 114.73, 65.74, 58.15, 45.74, 43.49, 43.15. MS (ESI^+^) *m/z* 389.3 [M+H]^+^.

**2-([1,1’-biphenyl]-4-yl)*-N*-(4-(2-(difluoromethoxy)benzyl)acetamide (4i).**

Prepared according to general procedure B using 4-biphenylacetic acid (100 mg, 0.471 mmol) and (2-(difluoromethoxy)phenyl)methanamine (68 µL, 0.471 mmol). The crude was recrystallized from toluene yielding colorless crystals (needle shape) (102 mg, 0.278 mmol, 59% yield). ^1^H NMR (400 MHz, CDCl_3_) δ 7.58 (dd, *J* = 8.4, 2.4 Hz, 4H), 7.45 (t, *J* = 7.5 Hz, 2H), 7.40 – 7.35 (m, 1H), 7.33 (dd, *J* = 7.9, 1.8 Hz, 3H), 7.28 (dd, *J* = 7.8, 1.8 Hz, 1H), 7.16 (td, *J* = 7.5, 1.1 Hz, 1H), 7.12 – 7.01 (m, 1H), 6.68 – 6.24 (m, 1H), 5.89 (s, 1H), 4.46 (d, *J* = 6.1 Hz, 2H), 3.64 (s, 2H). ^13^C NMR (101 MHz, CDCl_3_) δ 170.80, 149.38, 140.59, 140.40, 133.66, 130.33, 129.86, 129.54, 128.99, 128.84, 127.78, 127.44, 127.06, 125.66, 118.45, 115.97, 113.38, 77.24, 43.43, 38.86. MS (ESI^+^) *m/z* 368.3 [M+H]^+^.

**4-((2-([1,1'-biphenyl]-4-yl)acetamido)methyl)benzoic acid (4j).**

An aqueous solution of LiOH (445 µL, 0.890 mmol, 4 eq, 2M) was added to a solution of methyl 4-((2-([1,1'-biphenyl]-4-yl)acetamido)methyl)benzoate (**4f**) (80.0 mg, 0.223 mmol, 1 eq) in THF:MeOH (1:1). The reaction was left to stir at room temperature for 18 hours. After concentration under reduced pressure, the reaction mixture was acidified with HCl (1M) to pH 3. The precipitate was filtered off, washed with cold water and dried to give the pure compound **4j**, as a white powder (55 mg, 0.159 mmol, 72% yield). ^1^H NMR (400 MHz, DMSO-*d_6_*) δ 12.91 (s, 1H), 8.69 (t, *J* = 6.0 Hz, 1H), 7.89 (d, *J* = 8.0 Hz, 2H), 7.65 (d, *J* = 8.0 Hz, 2H), 7.61 (d, *J* = 7.9 Hz, 2H), 7.46 (t, *J* = 7.6 Hz, 2H), 7.36 (t, *J* = 7.8 Hz, 5H), 4.35 (d, *J* = 5.9 Hz, 2H), 3.54 (s, 2H). ^13^C NMR (101 MHz, DMSO) δ 170.71, 167.64, 145.16, 140.46, 138.82, 136.04, 130.10, 129.85, 129.74, 129.39, 127.76, 127.67, 127.03, 42.47, 42.41. MS (ESI^+^) *m/z* 346.2 [M+H]^+^.

**2-([1,1’-biphenyl]-4-yl)*-N*-(4-(4-hydroxybenzy)acetamide (4k).**

To a solution of 2-([1,1’-biphenyl]-4-yl)*-N*-(4-methoxybenzyl)acetamide (**4d**) (100 mg, 0.302 mmol, 1 eq) in anhydrous DCM (5 mL), boron tribromide (603 µL, 0.604 mmol, 2 eq, 1 M in DCM,) was added at 0 °C under inert atmosphere. The resulting mixture was allowed to warm to room temperature and stir for 18 hours. Ice cold water (5 mL) and DCM (10 mL) were added to the reaction mixture. The organic phase was washed with a saturated aqueous solution of NaHCO_3_ (3 x 5 mL), brine, dried over sodium sulphate and concentrated under reduced pressure, yielding the pure compound **4k** as a white solid (77 mg, 0.243 mmol, 80% yield). ^1^H NMR (400 MHz, DMSO) δ 9.29 (s, 1H), 8.46 (d, *J* = 6.2 Hz, 1H), 7.65 (dd, *J* = 8.0, 2.3 Hz, 2H), 7.62 – 7.54 (m, 2H), 7.50 – 7.41 (m, 2H), 7.35 (d, *J* = 6.7 Hz, 3H), 7.05 (dd, *J* = 8.5, 2.5 Hz, 2H), 6.77 – 6.57 (m, 2H), 4.15 (d, *J* = 3.7 Hz, 2H), 3.49 (s, 2H). ^13^C NMR (101MHz, DMSO) δ 169.81, 156.26, 140.03, 138.27, 135.78, 129.59, 129.51, 128.91, 128.64, 127.27, 126.55, 126.51, 115.01, 41.97, 41.84. MS (ESI^+^) *m/z* 318.2 [M+H]^+^.

***N*-(4-((dimethylamino)methyl)benzyl)-2-(5-methyl-2-phenyloxazol-4-yl)acetamide (5c)**

Prepared according to general procedure B using 2-(5-methyl-2-phenyloxazol-4-yl)acetic acid (100 mg, 0.460 mmol) and 1-(4-(aminomethyl)phenyl)-*N*,*N*-dimethylmethanamine hydrochloride (76 mg, 0.460 mmol). The crude was recrystallized from toluene yielding a light brown solid (103 mg, 0.283 mmol, 62% yield). ^1^H NMR (400 MHz, CDCl_3_) δ 7.99 – 7.85 (m, 2H), 7.48 – 7.38 (m, 3H), 7.31 (s, 1H), 7.24 (s, 4H), 4.47 (d, *J* = 5.6 Hz, 2H), 3.51 (s, 2H), 3.39 (s, 2H), 2.36 (s, 3H), 2.21 (s, 6H). ^13^C NMR (101 MHz, CDCl_3_) δ 169.22, 159.99, 145.31, 137.99, 137.00, 130.25, 130.01, 129.39, 128.77, 127.44, 127.21, 125.99, 64.02, 45.34, 43.37, 33.56, 10.21. MS (ESI^+^) *m/z* 364.3 [M+H]^+^.

**(*R*)-*N*-(4-((dimethylamino)methyl)benzyl)-2-(2-fluoro-[1,1'-biphenyl]-4-yl)propenamide (5d)**

Prepared according to general procedure B using (*R*)-flurbiprofen (100 mg, 0.409 mmol) and 1‑(4-(aminomethyl)phenyl)-*N*,*N*-dimethylmethanamine hydrochloride (82.1 mg, 0.409 mmol). The crude was recrystallized from toluene yielding a white powder (97 mg, 0.248 mmol, 61% yield). ^1^H NMR (400 MHz, CDCl_3_) δ 7.53 (dd, *J* = 7.2, 1.6 Hz, 2H), 7.48 – 7.34 (m, 4H), 7.24 (d, *J* = 8.0 Hz, 2H), 7.19 – 7.10 (m, 4H), 5.73 (s, 1H), 4.42 (qd, *J* = 14.8, 5.8 Hz, 2H), 3.61 (q, *J* = 7.1 Hz, 1H), 3.39 (s, 2H), 2.22 (s, 6H), 1.58 (d, *J* = 7.1 Hz, 3H). ^13^C NMR (101 MHz, CDCl_3_) δ 173.27, 159.76 (d, ^1^*J*_C‑F_ = 249 Hz), 142.59 (d, ^3^*J*_C‑F_ = 7.5 Hz), 138.17, 136.90, 135.33, 131.11 (d, ^4^*J*_C‑F_ = 4.1 Hz), 129.44, 128.90 (d, ^4^*J*_C‑F_ = 3.0 Hz), 128.48, 127.86 (d, ^2^*J*_C‑F_ = 13.6 Hz), 127.73, 127.53, 123.64 (d, ^4^*J*_C‑F_ = 3.5 Hz), 115.19 (d, ^2^*J*_C‑F_ = 23.5 Hz), 63.93, 46.70, 45.32, 43.46, 18.61. HRMS (ESI^+^) *m/z* [M+H]^+^ calculated for C_25_H_27_N_2_O_1_F_1_ 391.2180, found 391.2180.

**4-((2-([1,1'-biphenyl]-4-yl)acetamido)methyl)-*N*,*N*-dimethylbenzamide (4l)**

Prepared according to general procedure B using 4-biphenylacetic acid (150 mg, 0.707 mmol) and 4-(aminomethyl)-*N*,*N*-dimethylbenzamide (126 mg, 0.707 mmol). The crude was purified by normal-phase flash chromatography (7% MeOH in DCM) and subsequently recrystallized from acetonitrile yielding white crystals (192 mg, 0.515 mmol, 73% yield). ^1^H NMR (400 MHz, CDCl_3_) δ 7.63 – 7.53 (m, 4H), 7.44 (dd, *J* = 8.4, 6.9 Hz, 2H), 7.40 – 7.28 (m, 5H), 7.21 (d, *J* = 7.8 Hz, 2H), 5.98 (s, 1H), 4.44 (d, *J* = 5.9 Hz, 2H), 3.68 (s, 2H), 3.09 (s, 3H), 2.95 (s, 3H). ^13^C NMR (101 MHz, CDCl_3_) δ 171.33, 171.00, 140.47, 140.40, 139.78, 135.39, 133.67, 129.91, 128.86, 127.81, 127.48, 127.44, 127.05, 43.41, 43.21, 39.60, 35.38. MS (ESI^+^) *m/z* 373.3 [M+H]^+^.

**Tert-butyl(4-((2-([1,1'-biphenyl]-4-yl)acetamido)methyl)phenyl)carbamate (4m)**

Prepared according to general procedure B using 4-biphenylacetic acid (150 mg, 0.707 mmol) and tert-butyl (4-(aminomethyl)phenyl)carbamate (157 mg, 0.707 mmol). The crude was recrystallized from acetonitrile yielding an off-white shiny powder (256 mg, 0.615 mmol, 87%). ^1^H NMR (400 MHz, CDCl_3_) δ 7.66 – 7.51 (m, 4H), 7.44 (t, *J* = 7.5 Hz, 2H), 7.38 – 7.24 (m, 5H), 7.12 (d, *J* = 8.6 Hz, 2H), 6.52 (s, 1H), 5.75 (s, 1H), 4.36 (d, *J* = 5.8 Hz, 2H), 3.64 (s, 2H), 1.50 (s, 9H). ^13^C NMR (101 MHz, CDCl_3_) δ 170.76, 152.73, 140.52, 140.33, 137.70, 133.72, 132.67, 129.90, 128.84, 128.40, 127.77, 127.44, 127.06, 118.75, 80.63, 43.46, 43.20, 28.34. MS (ESI^+^) *m/z* 416.2 [M+H]^+^.

**Tert-butyl(4-((2-([1,1'-biphenyl]-4-yl)acetamido)methyl)phenyl)carbamate (5e)**

Prepared according to general procedure B using 4′-[(dimethylamino)carbonyl][1,1′-biphenyl]-4-acetic acid (100 mg, 0.371 mmol) and (4-(aminomethyl)phenyl)-*N*,*N*-dimethylmethanamine hydrochloride (61.0 mg, 0.371 mmol). The crude was purified by normal-phase flash chromatography (8% MeOH in DCM) and subsequently recrystallized from acetonitrile yielding a white powder (113 mg, 0.263 mmol, 71% yield). ^1^H NMR (400 MHz, CDCl_3_) δ 7.65 – 7.52 (m, 4H), 7.49 (d, *J* = 8.3 Hz, 2H), 7.36 (d, *J* = 8.2 Hz, 2H), 7.24 (d, *J* = 8.1 Hz, 2H), 7.16 (d, *J* = 8.1 Hz, 2H), 5.81 (s, 1H), 4.42 (d, *J* = 5.8 Hz, 2H), 3.66 (s, 2H), 3.37 (s, 2H), 3.13 5(s, 3H), 3.03 (s, 3H), 2.21 (s, 6H). ^13^C NMR (101 MHz, CDCl_3_) δ 171.39, 170.65, 141.74, 139.41, 138.29, 136.86, 135.23, 134.35, 130.00, 129.42, 127.74, 127.51, 126.96, 64.01, 44.40, 43.42, 39.68, 35.43. MS (ESI^+^) *m/z* 430.4 [M+H]^+^.

***N*-(4-((1H-pyrazol-1-yl)methyl)benzyl)-2-([1,1'-biphenyl]-4-yl)acetamide (4n)**

Prepared according to general procedure B using 4-biphenylacetic acid (150 mg, 0.707 mmol) and 4-(1H-Pyrazol-1-ylmethyl)benzylaminehydrochloride (158 mg, 0.707 mmol). The crude was purified by normal-phase flash chromatography (8% MeOH in DCM) and subsequently recrystallized from acetonitrile yielding an off-white powder (225 mg, 0.590 mmol, 83% yield). ^1^H NMR (400 MHz, DMSO) δ 8.80 – 8.67 (m, 1H), 7.80 (s, 1H), 7.64 (d, *J* = 10.4 Hz, 2H), 7.58 (d, *J* = 11.1 Hz, 2H), 7.51 – 7.40 (m, 3H), 7.40 – 7.30 (m, 3H), 7.24 – 7.17 (m, 2H), 7.15 (dd, *J* = 8.2, 2.4 Hz, 2H), 6.24 (s, 1H), 5.28 (s, 2H), 4.23 (d, *J* = 8.6 Hz, 2H), 3.51 (s, 2H). ^13^C NMR (101 MHz, DMSO) δ 170.52, 140.49, 139.36, 139.31, 138.74, 136.69, 136.20, 130.49, 130.09, 129.38, 128.01, 127.73, 127.01, 126.96, 105.87, 54.85, 42.40, 42.38. MS (ESI^+^) *m/z* 382.3 [M+H]^+^.

**2-([1,1'-biphenyl]-4-yl)-*N*-(4-aminobenzyl)acetamide (4o)**

Trifluoroacetic acid (1.00 mL, 15.4 mmol) was added to a suspension of 2-([1,1'-biphenyl]-4-yl)-N-(4-aminobenzyl)acetamide (**4m**) (150 mg, 0.358 mmol) in dichloromethane. The reaction mixture was stirred at room temperature for 2 hours. Then, it was neutralized with a saturated aqueous solution of sodium carbonate (8 mL). The aqueous layer was extracted with dichloromethane (3 x 15 mL). The combined organic layers were dried over sodium sulphate, filtered and concentrated under reduced pressure to afford white crystals (88 mg, 0.278 mmol, 78% yield). ^1^H NMR (400 MHz, CDCl_3_) δ 7.57 (td, *J* = 5.9, 3.0 Hz, 4H), 7.44 (dd, *J* = 8.4, 6.9 Hz, 2H), 7.39 – 7.29 (m, 3H), 7.00 (d, *J* = 8.3 Hz, 2H), 6.61 (d, *J* = 8.4 Hz, 2H), 5.66 (s, 1H), 4.31 (d, *J* = 5.6 Hz, 2H), 3.64 (s, 2H). ^13^C NMR (101 MHz, CDCl_3_) δ 170.65, 145.83, 140.57, 140.26, 133.84, 129.90, 128.99, 128.84, 127.90, 127.73, 127.42, 127.06, 115.19, 43.50, 43.37. MS (ESI^+^) *m/z* 317.2 [M+H]^+^.

**2-([1,1'-biphenyl]-4-yl)-*N*-(pyrimidin-2-ylmethyl)acetamide (4p)**

Prepared according to general procedure B using 4-biphenylacetic acid (150 mg, 0.707 mmol) and pyrimidin-2-ylmethanamine (68 µL, 0.707 mmol). The crude was recrystallized from acetonitrile yielding a white crystalline solid (172 mg, 0.567 mmol, 80% yield). ^1^H NMR (400 MHz, CDCl_3_) δ 8.66 (d, *J* = 4.9 Hz, 2H), 7.60 (d, *J* = 8.2 Hz, 4H), 7.51 – 7.39 (m, 4H), 7.35 (t, *J* = 7.4 Hz, 1H), 7.18 (t, *J* = 4.9 Hz, 1H), 6.80 (s, 1H), 4.70 (d, *J* = 4.7 Hz, 2H), 3.73 (s, 2H). ^13^C NMR (101 MHz, CDCl_3_) δ 170.97, 165.74, 157.17, 140.73, 140.16, 133.96, 129.96, 128.83, 127.63, 127.37, 127.07, 119.51, 45.42, 43.41. MS (ESI^+^) *m/z* 304.1 [M+H]^+^.

***N*-(4-((dimethylamino)methyl)benzyl)-2-(4-(pyridin-2-yl) phenyl)acetamide (5f)**

Prepared according to general procedure B using 2-(4-(pyridin-2-yl)phenyl)acetic acid (**9**) (150 mg, 0.703 mmol) and (4-(aminomethyl)phenyl)-*N*,*N*-dimethylmethanamine hydrochloride (141 mg, 0.703 mmol). The crude was recrystallized from acetonitrile yielding a crystalline solid (162 mg, 0.451 mmol, 64% yield).^1^H NMR (400 MHz, CDCl_3_) δ 8.68 (ddd, *J* = 4.8, 1.8, 1.0 Hz, 1H), 8.03 – 7.89 (m, 2H), 7.81 – 7.66 (m, 2H), 7.45 – 7.31 (m, 2H), 7.25 – 7.19 (m, 3H), 7.14 (d, *J* = 8.1 Hz, 2H), 5.76 (s, 1H), 4.40 (d, *J* = 5.8 Hz, 2H), 3.68 (s, 2H), 3.38 (s, 2H), 2.21 (s, 6H). ^13^C NMR (101 MHz, CDCl_3_) δ 170.62, 156.87, 149.74, 138.65, 138.00, 136.87, 135.51, 129.95, 129.45, 127.61, 127.52, 122.29, 120.54, 63.94, 45.32, 43.59, 43.40. MS (ESI^+^) *m/z* 360.2 [M+H]^+^.

***N*-(4-((dimethylamino)methyl)benzyl)-2-(4-(pyrimidin-2-yl) phenyl)acetamide (5g)**

Prepared according to general procedure B using 2-(4-(pyrimidin-2-yl)phenyl)acetic acid (**10**) (100 mg, 0,467 mmol) and (4-(aminomethyl)phenyl)-*N*,*N*-dimethylmethanamine hydrochloride (93.7 mg, 0,467 mmol). The crude was recrystallized from acetonitrile yielding a crystalline solid (114 mg, 0.316 mmol, 68% yield). ^1^H NMR (400 MHz, DMSO) δ 8.90 (d, *J* = 4.8 Hz, 2H), 8.75 (t, *J* = 6.0 Hz, 1H), 8.37 – 8.29 (m, 2H), 7.52 – 7.39 (m, 5H), 7.35 – 7.25 (m, 2H), 4.32 (d, *J* = 5.9 Hz, 2H), 4.18 (s, 2H), 3.59 (s, 2H), 2.63 (s, 6H). ^13^C NMR (101 MHz, DMSO) δ 170.37, 163.69, 158.17, 141.26, 139.80, 135.96, 131.40, 129.91, 129.60, 128.06, 127.88, 120.27, 59.72, 42.67, 42.34, 42.03. MS (ESI^+^) *m/z* 361.2 [M+H]^+^.

**2-([1,1'-biphenyl]-4-yl)-*N*-(pyridin-2-ylmethyl)acetamide (4q)**

Prepared according to general procedure B using 4-biphenylacetic acid (150 mg, 0.707 mmol) and pyridin-2-ylmethanamine (73 µL, 0.707 mmol). The crude was recrystallized from acetonitrile yielding white crystals (116 mg, 0.384 mmol, 54% yield). ^1^H NMR (400 MHz, CDCl_3_) δ 8.51 ‑ 8.41 (m, 1H), 7.64 (td, *J* = 7.8, 1.6 Hz, 1H), 7.61 – 7.54 (m, 4H), 7.44 (t, *J* = 7.5 Hz, 2H), 7.41 ‑ 7.29 (m, 3H), 7.22 (d, *J* = 7.8 Hz, 1H), 7.17 (dd, *J* = 7.5, 5.0 Hz, 1H), 6.75 (s, 1H), 4.55 (d, *J* = 5.0 Hz, 2H), 3.70 (s, 2H). ^13^C NMR (101 MHz, CDCl_3_) δ 170.95, 156.25, 149.02, 140.69, 140.19, 136.75, 133.87, 129.87, 128.80, 127.65, 127.34, 127.05, 122.35, 122.00, 44.62, 43.38. MS (ESI^+^) *m/z* 303.2 [M+H]^+^.

***N*-(4-((dimethylamino)methyl)benzyl)-2-(4-(5-methoxypyridin-3-yl)phenyl)acetamide (5h)**

Prepared according to general procedure B using 2-(4-(5-methoxypyridin-3-yl)phenyl)acetic acid (**11**) (100 mg, 0.411 mmol) and (4-(aminomethyl)phenyl)-*N*,*N*-dimethylmethanamine hydrochloride (82.5 mg, 0.411 mmol). The crude was recrystallized from acetonitrile yielding a white solid (113 mg, 0.290 mmol, 70% yield). ^1^H NMR (400 MHz, CDCl_3_) δ 8.42 (d, *J* = 1.9 Hz, 1H), 8.28 (d, *J* = 2.8 Hz, 1H), 7.55 (d, *J* = 7.8 Hz, 2H), 7.39 (d, *J* = 7.8 Hz, 2H), 7.35 – 7.27 (m, 3H), 7.19 (d, *J* = 7.7 Hz, 2H), 5.95 (s, 1H), 4.43 (d, *J* = 5.8 Hz, 2H), 3.91 (s, 3H), 3.67 (s, 2H), 3.54 (s, 2H), 2.32 (s, 6H). ^13^C NMR (101 MHz, CDCl_3_) δ 170.54, 155.80, 140.58, 137.78, 136.79, 136.74, 136.23, 134.89, 130.14, 129.89, 127.84, 127.75, 118.98, 63.28, 55.67, 44.61, 43.39. MS (ESI^+^) *m/z* 390.2 [M+H]^+^.

***N*-(4-((dimethylamino)methyl)benzyl)-2-(4-(5-methoxypyridin-2-yl)phenyl)acetamide (5i)**

Prepared according to general procedure B using 2-(4-(5-methoxypyridin-2-yl)phenyl)acetic acid (**12**) (80.0 mg, 0.329 mmol) and (4-(aminomethyl)phenyl)-*N*,*N*-dimethylmethanamine hydrochloride (66.0 mg, 0.329 mmol). The crude was recrystallized from acetonitrile yielding a white solid (81 mg, 0.208 mmol, 63% yield). ^1^H NMR (400 MHz, CDCl_3_) δ 8.39 (d, *J* = 2.9 Hz, 1H), 8.04 ‑ 7.79 (m, 2H), 7.66 (d, *J* = 8.8 Hz, 1H), 7.41 – 7.32 (m, 2H), 7.30 – 7.22 (m, 3H), 7.15 (d, *J* = 8.0 Hz, 2H), 5.76 (s, 1H), 4.41 (d, *J* = 5.8 Hz, 2H), 3.91 (s, 3H), 3.68 (s, 2H), 3.41 (s, 2H), 2.24 (s, 6H). ^13^C NMR (101 MHz, CDCl_3_) δ 170.72, 154.93, 149.47, 138.38, 137.80, 137.22, 136.96, 134.59, 129.89, 129.45, 127.50, 127.06, 121.25, 120.75, 63.88, 55.69, 45.26, 43.56, 43.36. MS (ESI^+^) *m/z* 390.3 [M+H]^+^.

**(*S*)-*N*-(4-((dimethylamino)methyl)benzyl)-2-(2-fluoro-[1,1'-biphenyl]-4-yl)propenamide (5j)**

Prepared according to general procedure B using (*S*)-flurbiprofen (95.0 mg, 0.389 mmol) and (4‑(aminomethyl)phenyl)-*N*,*N*-dimethylmethanamine hydrochloride (78.0 mg, 0.389 mmol). The crude was purified by normal-phase flash chromatography (8% MeOH in DCM) yielding an amorphous colorless solid (104 mg, 0.266 mmol, 69% yield). ^1^H NMR (400 MHz, CDCl_3_) δ 7.51 (d, *J* = 7.6 Hz, 2H), 7.46 – 7.30 (m, 4H), 7.22 (d, *J* = 7.7 Hz, 2H), 7.18 – 7.02 (m, 4H), 6.14 (t, *J* = 5.8 Hz, 1H), 4.37 (qd, *J* = 14.9, 5.7 Hz, 2H), 3.60 (q, *J* = 7.1 Hz, 1H), 3.37 (s, 2H), 2.20 (s, 6H), 1.54 (d, *J* = 7.2 Hz, 3H). ^13^C NMR (101 MHz, CDCl_3_) δ 173.49, 160.02 (d,^1^ *J*_C-F_ = 249 Hz ), 142.83, 142.76, 137.89, 137.11, 135.42, 135.40, 131.01 (d,^3^*J*_C-F_ = 3.9 Hz ), 129.46, 129.01 (d,
^4^*J*_C-F_ = 2.9 Hz°), 128.94, 128.51, 127.82 (d,^2^*J*_C‑F_ = 13.5 Hz ), 127.75, 127.52, 123.64 (d,^4^ *J*_C-F_ = 3.3 Hz ), 115.34 (d,^2^*J*_C‑F_ = 23.5 Hz ), 63.90, 46.55, 46.53, 45.28, 43.41, 18.63. HRMS (ESI^+^) *m/z* [M+H]^+^ calculated for C_25_H_27_N_2_O_1_F_1_ 391.2180, found 391.2176.

**2-([1,1'-biphenyl]-4-yl)-*N*-((5-(dimethylamino)pyridin-2-yl)methyl)acetamide (4r)**

Prepared according to general procedure B using 4-biphenylacetic acid (150 mg, 0.707 mmol) and 6-(aminomethyl)-*N*,*N*-dimethylpyridin-3-amine (107 mg, 0.707 mmol). The crude was recrystallized from acetonitrile yielding a white solid (220 mg, 0.637 mmol, 90% yield). ^1^H NMR (400 MHz, CDCl_3_) δ 8.01 (d, *J* = 2.3 Hz, 1H), 7.56 (t, *J* = 7.1 Hz, 4H), 7.51 – 7.39 (m, 3H), 7.33 (dd, *J* = 13.5, 7.5 Hz, 3H), 6.50 (d, *J* = 8.8 Hz, 1H), 6.09 (s, 1H), 4.28 (d, *J* = 5.7 Hz, 2H), 3.63 (s, 2H), 3.10 (s, 6H). ^13^C NMR (101 MHz, CDCl_3_) δ 170.98, 157.37, 144.94, 140.54, 140.24, 138.96, 133.75, 129.90, 128.84, 127.72, 127.42, 127.05, 121.35, 106.86, 43.33, 40.65, 38.65. MS (ESI^+^) *m/z* 346.2 [M+H]^+^.

**2-([1,1'-biphenyl]-4-yl)-*N*-(pyrimidin-4-ylmethyl)acetamide (4s)**

Prepared according to general procedure B using 4-biphenylacetic acid (150 mg, 0.707 mmol) and pyrimidin-4-ylmethanamine (77.0 mg, 0.707 mmol). The obtained solid was washed with acetonitrile yielding a white solid (112 mg, 0.369 mmol, 52% yield). ^1^H NMR (400 MHz, CDCl_3_) δ 9.10 (d, *J* = 1.4 Hz, 1H), 8.65 (d, *J* = 5.2 Hz, 1H), 7.64 – 7.55 (m, 4H), 7.49 – 7.41 (m, 2H), 7.41 – 7.32 (m, 3H), 7.25 – 7.20 (m, 1H), 6.58 (s, 1H), 4.54 (d, *J* = 5.4 Hz, 2H), 3.71 (s, 2H). ^13^C NMR (101 MHz, CDCl_3_) δ 171.24, 165.12, 158.58, 157.12, 140.53, 140.46, 133.52, 129.89, 128.87, 127.80, 127.48, 127.06, 119.14, 44.03, 43.30. MS (ESI^+^) *m/z* 304.2 [M+H]^+^.

***N*-(4-((dimethylamino)methyl)benzyl)-2-(4-(2-fluoropyridin-4-yl)phenyl)acetamide (5k)**

Prepared according to general procedure B using 2-(4-(2-fluoropyridin-4-yl)phenyl)acetic acid (150 mg, 0.649 mmol) and (4‑(aminomethyl)phenyl)-*N*,*N*-dimethylmethanamine hydrochloride (130 mg, 0.649 mmol). The crude was recrystallized from acetonitrile yielding a white solid (104 mg, 0.276 mmol, 43% yield). ^1^H NMR (400 MHz, CDCl_3_) δ 8.20 (ddd, *J* = 4.9, 1.9, 1.2 Hz, 1H), 7.86 (ddd, *J* = 9.7, 7.4, 2.0 Hz, 1H), 7.55 (dd, *J* = 8.2, 1.8 Hz, 2H), 7.39 (d, *J* = 8.3 Hz, 2H), 7.28 (ddd, *J* = 7.5, 4.8, 1.6 Hz, 3H), 7.18 (d, *J* = 8.2 Hz, 2H), 5.80 (s, 1H), 4.43 (d, *J* = 5.8 Hz, 2H), 3.67 (s, 2H), 3.47 (s, 2H), 2.27 (s, 6H). ^13^C NMR (101 MHz, CDCl_3_) δ 170.43, 160.39 (*J_C-F_* = 240 Hz), 146.45 (*J_C-F_* = 14.3 Hz), 140.6 (*J_C-F_* = 4.38 Hz), 137.29, 136.99, 135.17, 133.0 (*J_C-F_* = 5.05 Hz), 129.82, 129.68, 129.41 (*J_C-F_* = 3.01 Hz), 127.65, 123.34 (*J_C-F_*  = 28.1 Hz), 121.90 (*J _C-F_ =* 4.26 Hz), 63.62, 44.98, 43.47, 43.43. MS (ESI^+^) *m/z* 378.3 [M+H]^+^.

***N*-(5-chloro-2-(4-methylpiperazin-1-yl)phenyl)-2-(5-methyl-2-phenyloxazol-4-yl)acetamide (2)**

Prepared according to general procedure B using 2-(5-methyl-2-phenyloxazol-4-yl)acetic acid (140 mg, 0.644 mmol) and 5-chloro-2-(4-methylpiperazin-1-yl)aniline hydrochloride (169 mg, 0.644 mmol). The crude was recrystallized from acetonitrile yielding an off-white solid (218 mg, 0.487 mmol, 76% yield). ^1^H NMR (400 MHz, CDCl_3_) δ 8.92 (s, 1H), 8.46 (d, *J* = 1.9 Hz, 1H), 8.08 – 7.97 (m, 2H), 7.50 – 7.40 (m, 3H), 7.00 (d, *J* = 2.8 Hz, 2H), 3.70 (s, 2H), 2.79 – 2.64 (m, 4H), 2.42 (s, 3H), 2.23 (s, 4H), 2.00 (s, 3H). ^13^C NMR (101 MHz, CDCl_3_) δ 167.57, 160.31, 146.23, 139.94, 134.24, 130.76, 130.45, 129.86, 128.87, 127.39, 126.25, 123.85, 121.53, 119.92, 55.05, 52.06, 45.43, 35.66, 10.41. HRMS (ESI^+^) *m/z* [M+H]^+^ calculated for C_23_H_25_N_4_O_2_Cl_1_ 425.1739, found 425.1741.

***N*-(4-((dimethylamino)methyl)benzyl)-2-(4-(pyrimidin-5-yl)phenyl)acetamide (5l)**

Prepared according to general procedure B using 2-(4-(pyrimidin-5-yl)phenyl)acetic acid (100 mg, 0.467 mmol) and (4‑(aminomethyl)phenyl)-*N*,*N*-dimethylmethanamine hydrochloride (94.0 mg, 0.467 mmol). The crude was recrystallized from acetonitrile yielding a white solid (125 mg, 0.329 mmol, 71% yield). ^1^H NMR (400 MHz, CDCl_3_) δ 9.20 (d, *J* = 2.4 Hz, 1H), 8.93 (d, *J* = 2.4 Hz, 2H), 7.56 (dd, *J* = 8.3, 2.5 Hz, 2H), 7.44 (dd, *J* = 8.3, 2.5 Hz, 2H), 7.28 (d, *J* = 2.4 Hz, 2H), 7.18 (dd, *J* = 8.1, 2.5 Hz, 2H), 5.86 (s, 1H), 4.43 (dd, *J* = 6.1, 2.4 Hz, 2H), 3.67 (d, *J* = 2.5 Hz, 2H), 3.47 (d, *J* = 2.6 Hz, 2H), 2.33 – 2.20 (m, 6H). ^13^C NMR (101 MHz, CDCl_3_) δ 170.22, 157.57, 154.83, 137.23, 137.08, 135.89, 133.83, 133.33, 130.50, 129.69, 127.70, 127.53, 63.60, 44.96, 43.50, 43.35. MS (ESI^+^) *m/z* 361.2 [M+H]^+^.

***N*-(4-((dimethylamino)methyl)benzyl)-2-(4-(pyridin-4-yl)phenyl)acetamide (5m)**

Prepared according to general procedure B using 2-(4-(pyridin-4-yl)phenyl)acetic acid (**6**) (125 mg, 0.586 mmol) and (4‑(aminomethyl)phenyl)-*N*,*N*-dimethylmethanamine hydrochloride (118 mg, 0.586 mmol). The crude was recrystallized from acetonitrile yielding a yellowish amorphous solid (155 mg, 0.410 mmol, 70% yield). ^1^H NMR (400 MHz, DMSO) δ 8.68 (t, *J* = 6.0 Hz, 1H), 8.65 – 8.56 (m, 2H), 7.76 (d, *J* = 8.3 Hz, 2H), 7.72 – 7.66 (m, 2H), 7.45 – 7.37 (m, 4H), 7.30 (d, *J* = 7.8 Hz, 2H), 4.31 (d, *J* = 6.0 Hz, 2H), 4.11 (s, 2H), 3.57 (s, 2H), 2.61 (s, 6H). ^13^C NMR (101 MHz, DMSO) δ 170.43, 150.70, 147.21, 141.09, 138.13, 135.71, 131.11, 130.37, 128.35, 127.91, 127.14, 121.50, 60.34, 42.57, 42.47, 42.34. MS (ESI^+^) *m/z* 360.2 [M+H]^+^.

**2-(4'-(dimethylamino)-[1,1'-biphenyl]-4-yl)-*N*-(4-((dimethylamino)methyl)benzyl)acetamide (5n)**

Prepared according to general procedure B using 2-(4'-(dimethylamino)-[1,1'-biphenyl]-4-yl)acetic acid (**13**) (80.0 mg, 0.313 mmol) and (4‑(aminomethyl)phenyl)-*N*,*N*-dimethylmethanamine hydrochloride (62.9 mg, 0.313 mmol). The crude was recrystallized from acetonitrile yielding an off-white solid (79.0 mg, 0.197 mmol, 63% yield). ^1^H NMR (400 MHz, CDCl_3_) δ 7.53 (d, *J* = 8.2 Hz, 2H), 7.48 (d, *J* = 8.8 Hz, 2H), 7.29 (d, *J* = 8.1 Hz, 2H), 7.25 (d, *J* = 8.2 Hz, 3H), 7.15 (d, *J* = 8.0 Hz, 2H), 6.80 (d, *J* = 8.8 Hz, 2H), 5.75 (s, 1H), 4.42 (d, *J* = 5.9 Hz, 2H), 3.65 (s, 2H), 3.42 (s, 2H), 3.00 (s, 6H), 2.24 (s, 6H). ^13^C NMR (101 MHz, CDCl_3_) δ 171.06, 150.08, 140.37, 132.22, 129.85, 129.51, 128.36, 127.58, 127.50, 127.48, 126.84, 112.75, 63.81, 45.17, 43.49, 43.32, 40.56. MS (ESI^+^) *m/z* 402.3 [M+H]^+^.

**4'-(2-((4-((dimethylamino)methyl)benzyl)amino)-2-oxoethyl)-[1,1'-biphenyl]-4-carboxylic acid (5o)**

Prepared according to general procedure A using 2-(4-bromophenyl)-*N*-(4-((dimethylamino)methyl)benzyl)acetamide (150 mg, 0.415 mmol) and 4-carboxyphenylboronic acid (103 mg, 0.623 mmol). The crude was recrystallized from acetonitrile yielding a white solid (156 mg, 0.388 mmol, 93% yield). ^1^H NMR (400 MHz, DMSO) δ 11.32 (s, 1H), 8.91 (t, *J* = 6.0 Hz, 1H), 8.01 (d, *J* = 8.3 Hz, 2H), 7.79 (d, *J* = 8.2 Hz, 2H), 7.68 (d, *J* = 8.1 Hz, 2H), 7.54 (d, *J* = 7.9 Hz, 2H), 7.42 (d, *J* = 8.0 Hz, 2H), 7.30 (d, *J* = 7.9 Hz, 2H), 4.30 (d, *J* = 5.9 Hz, 2H), 4.22 (s, 2H), 3.58 (s, 2H), 2.62 (s, 6H). ^13^C NMR (101 MHz, DMSO) δ 170.57, 167.58, 144.57, 141.25, 137.55, 137.18, 131.52, 130.42, 130.28, 129.91, 129.41, 127.86, 127.26, 127.10, 59.31, 42.38, 42.29, 41.69. MS (ESI^+^) *m/z* 403.3 [M+H]^+^.

**2-(4-(pyridin-4-yl)phenyl)acetic acid (6)**

Prepared according to general procedure A using 4-pyridinylboronic acid (171 mg, 1.40 mmol) and 4-bromophenylacetic acid (200 mg, 0.930 mmol) to obtain 2-(4-(pyridin-4-yl)phenyl)acetic acid (137 mg, 0.642 mmol, 69% yield). ^1^H NMR (400 MHz, CDCl_3_) δ 8.77 (d, *J* = 6.3 Hz, 2H), 7.60 (d, *J* = 6.3 Hz, 2H), 7.46 (d, *J* = 8.3 Hz, 2H), 7.19 (d, *J* = 8.3 Hz, 2H), 3.62 (s, 2H). ^13^C NMR (101 MHz, CDCl_3_) δ 175.28, 149.59, 146.28, 132.93, 131.71, 131.16, 121.91, 121.25, 40.63. MS (ESI^+^) *m/z* 214.2 [M+H]^+^.

**2-(4-(2-fluoropyridin-4-yl)phenyl)acetic acid** **(7)**

Prepared according to general procedure A using (2-fluoropyridin-4-yl)boronic acid (246 mg, 1.74 mmol) and 4-bromophenylacetic acid (250 mg, 1.16 mmol); The crude was recrystallized from acetonitrile to obtain 2-(4-(2-fluoropyridin-4-yl)phenyl)acetic acid (199 mg, 0.861 mmol, 74% yield). ^1^H NMR (400 MHz, CDCl_3_) δ 8.21 (dt, *J* = 4.8, 1.5 Hz, 1H), 7.87 (ddd, *J* = 9.6, 7.4, 2.0 Hz, 1H), 7.55 (dd, *J* = 8.3, 1.8 Hz, 2H), 7.44 – 7.36 (m, 2H), 7.28 (ddd, *J* = 7.4, 4.8, 1.8 Hz, 1H), 3.73 (s, 2H). ^13^C NMR (101 MHz, CDCl_3_) δ 176.93, 160.41 (*J_C-F_* = 246 Hz), 146.32 (*J_C-F_* = 14.6 Hz), 140.74 (*J_C-F_* =4.36 Hz), 133.70, 132.90, 131.78, 131.14, 123.49 (*J_C-F_* = 28.48 Hz), 121.92 (*J_C-F_* = 4.61 Hz), 40.39. MS (ESI^+^) *m/z*  232.2 [M+H]^+^.

**2-(4-(pyrimidin-5-yl)phenyl)acetic acid (8)**

Prepared according to general procedure A using 5-pyrimidinylboronic acid (216 mg, 1.74 mmol) and 4-bromophenylacetic acid (250 mg, 1.16 mmol). The crude was recrystallized from acetonitrile yielding 2-(4-(pyrimidin-5-yl)phenyl)acetic acid (135 mg, 0.630 mmol, 54% yield). ^1^H NMR (400 MHz, DMSO) δ 9.18 (s, 1H), 9.14 (s, 2H), 7.79 – 7.73 (m, 2H), 7.48 – 7.39 (m, 2H), 3.66 (s, 2H). ^13^C NMR (101 MHz, DMSO) δ 172.92, 157.57, 155.08, 136.50, 133.49, 132.48, 130.86, 127.26, 40.76. MS (ESI^+^) *m/z* 215.2 [M+H]^+^.

**2-(4-(pyridin-2-yl)phenyl)acetic acid (9)**

Prepared according to general procedure A using 4-(carboxymethyl)phenylboronic acid pinacol ester (498 mg, 1.90 mmol) and 2-bromopyridine (200 mg, 120 µL, 1.27 mmol). The crude was recrystallized from acetonitrile yielding 2-(4-(pyridin-2-yl)phenyl)acetic acid (211 mg, 0.99 mmol, 78% yield). ^1^H NMR (400 MHz, DMSO) δ 8.82 (dd, *J* = 5.6, 1.6 Hz, 1H), 8.42 (d, *J* = 7.9 Hz, 1H), 8.29 (d, *J* = 8.1 Hz, 1H), 8.03 (d, *J* = 8.1 Hz, 2H), 7.81 (t, *J* = 6.5 Hz, 1H), 7.50 (d, *J* = 8.1 Hz, 2H), 3.71 (s, 2H). ^13^C NMR (101 MHz, DMSO) δ 172.78, 153.16, 145.13, 143.92, 138.89, 132.18, 130.82, 128.15, 124.93, 124.20, 40.79. MS (ESI^+^) *m/z* 214.2 [M+H]^+^.

**2-(4-(pyrimidin-2-yl)phenyl)acetic acid (10)**

Prepared according to general procedure A using 4-(carboxymethyl)phenylboronic acid pinacol ester (495 mg, 1.89 mmol) and 2-bromopyrimidine (200 mg, 1.26 mmol). The crude was recrystallized from acetonitrile yielding 2-(4-(pyridin-2-yl)phenyl)acetic acid (195 mg, 0.91 mmol, 72% yield). ^1^H NMR (400 MHz, DMSO) δ 12.45 (s, 1H), 8.90 (d, *J* = 4.8 Hz, 2H), 8.34 (d, *J* = 8.0 Hz, 2H), 7.62 – 7.25 (m, 3H), 3.67 (s, 2H). ^13^C NMR (101 MHz, DMSO) δ 172.93, 163.63, 158.18, 138.42, 136.11, 130.30, 128.05, 120.30, 40.97. MS (ESI^+^) *m/z* 215.2 [M+H]^+^.

**2-(4-(5-methoxypyridin-3-yl)phenyl)acetic acid (11)**

Prepared according to general procedure A using 4-(carboxymethyl)phenylboronic acid pinacol ester (376 mg, 1.44 mmol) and 2 3-bromo-5-methoxypyridine (180 mg, 0.96 mmol) to obtain 2-(4-(5-methoxypyridin-3-yl)phenyl)acetic acid (223 mg, 0.92 mmol, 96% yield). ^1^H NMR (400 MHz, DMSO) δ 12.41 (s, 1H), 8.48 (d, *J* = 1.8 Hz, 1H), 8.28 (d, *J* = 2.7 Hz, 1H), 7.75 – 7.64 (m, 2H), 7.61 (dd, *J* = 2.8, 1.9 Hz, 1H), 7.42 – 7.34 (m, 2H), 3.90 (s, 3H), 3.64 (s, 2H). ^13^C NMR (101 MHz, DMSO) δ 172.82, 155.86, 140.63, 136.92, 136.89, 135.19, 133.22, 129.90, 119.05, 55.71, 40.80. MS (ESI^+^) *m/z* 244.2 [M+H]^+^.

**2-(4-(5-methoxypyridin-2-yl)phenyl)acetic acid (12)**

Prepared according to general procedure A using 4-(carboxymethyl)phenylboronic acid pinacol ester (261 mg, 1.00 mmol) and 2 3-bromo-5-methoxypyridine (125 mg, 0.66 mmol) to obtain 2-(4-(5-methoxypyridin-2-yl)phenyl)acetic acid (140 mg, 0.58 mmol 86% yield). ^1^H NMR (400 MHz, CDCl_3_) δ 8.42 (s, 1H), 7.79 – 7.68 (m, 2H), 7.60 (d, *J* = 8.7 Hz, 1H), 7.37 (d, *J* = 7.9 Hz, 2H), 7.31 (dt, *J* = 9.0, 1.9 Hz, 1H), 3.91 (s, 3H), 3.68 (s, 2H). ^13^C NMR (101 MHz, DMSO) δ 173.72, 163.63, 154.89, 149.34, 138.60, 137.21, 133.59, 129.31, 127.26, 120.75, 55.77, 40.96. MS (ESI^+^) *m/z* 244.2 [M+H]^+^.

**2-(4'-(dimethylamino)-[1,1'-biphenyl]-4-yl)acetic acid (13)**

Prepared according to general procedure A using (4-(dimethylamino)phenyl)boronic acid (238 mg, 1.40 mmol) and 4-bromophenylacetic acid (200 mg, 0.96 mmol) to obtain 2-(4'-(dimethylamino)-[1,1'-biphenyl]-4-yl)acetic acid (197 mg, 0.77 mmol, 83% yield). ^1^H NMR (400 MHz, CDCl_3_) δ 7.52 (d, *J* = 7.9 Hz, 2H), 7.51 – 7.45 (m, 2H), 7.31 (d, *J* = 7.9 Hz, 2H), 6.89 – 6.72 (m, 2H), 3.68 (s, 2H), 2.99 (s, 6H). ^13^C NMR (101 MHz, DMSO) δ 171.88, 163.63, 159.90, 139.87, 131.92, 129.54, 128.36, 127.49, 127.18, 112.55, 42.81, 40.49. MS (ESI^+^) *m/z* 256.2 [M+H]^+^.

**1-(4-chloro-2-nitrophenyl)-4-methylpiperazine (14)**

1,4-dichloro-2-nitrobenzene (3.00 g, 15.6 mmol, 1 eq.) and potassium carbonate (4.32 g, 31.2 mmol, 2 eq.) were dissolved in DMF (40 mL), 1-methylpiperazine (2 mL, 18.8 mmol, 1.2 eq.) was then added. The reaction mixture was stirred and refluxed for 4 hours. Once the reaction was completed, the reaction mixture was diluted with water (40 mL) and extracted with DCM (3 x 20 mL). The organic layer was dried over sodium sulfate, filtered and concentrated under pressure and further concentrated under high vacuum to afford 1-(4-chloro-2-nitrophenyl)-4-methylpiperazine (3.80 g, 14.9 mmol, 95% yield). ^1^H NMR (400 MHz, CDCl_3_) δ 7.76 (d, *J* = 2.5 Hz, 1H), 7.43 (dd, *J* = 8.8, 2.5 Hz, 1H), 7.09 (d, *J* = 8.8 Hz, 1H), 3.16 – 3.00 (m, 4H), 2.59 (dd, *J* = 6.0, 3.6 Hz, 4H), 2.36 (s, 3H). ^13^C NMR (101 MHz, CDCl_3_) δ 142.68, 139.88, 132.40, 126.78, 125.59, 122.14, 55.03, 50.92, 46.18. MS (ESI^+^) *m/z* 256.2 [M+H]^+^.

**5-chloro-2-(4-methylpiperazin-1-yl)aniline hydrochloride (15)**

1-(4-chloro-2-nitrophenyl)-4-methylpiperazine (**14**) (3.00 g, 11.7 mmol, 1 eq.) was dissolved in a solution of hydrochloric acid in ethanol (40 mL, 2.5M), stannous chloride (8.90 g, 46.9 mmol, 4 eq.) was added and the reaction mixture was refluxed for 2 hours. Once the reaction was completed, water ( 30 mL) was added to the reaction mixture, which was cooled to room temperature and further cooled on ice. The precipitate was vacuum filtered and washed with water (3 x 15 mL), then dried in vacuum oven at 40 °C until the mass was constant, affording 5-chloro-2-(4-methylpiperazin-1-yl)aniline hydrochloride (1.93 g, 8.5 mmol, 73% yield). ^1^H NMR (400 MHz, CDCl_3_) δ 6.91 (d, *J* = 8.2 Hz, 1H), 6.73 – 6.64 (m, 2H), 4.03 (br s, 2H), 2.90 (t, *J* = 4.8 Hz, 4H), 2.56 (br s, 3H), 2.35 (s, 3H). ^13^C NMR (101 MHz, CDCl_3_) δ 142.75, 137.72, 129.80, 121.04, 118.18, 114.69, 55.84, 51.07, 46.24. MS (ESI^+^) *m/z* 226.2 [M+H]^+^.

**HRMS spectra of key molecules**

**Compound 1**

**
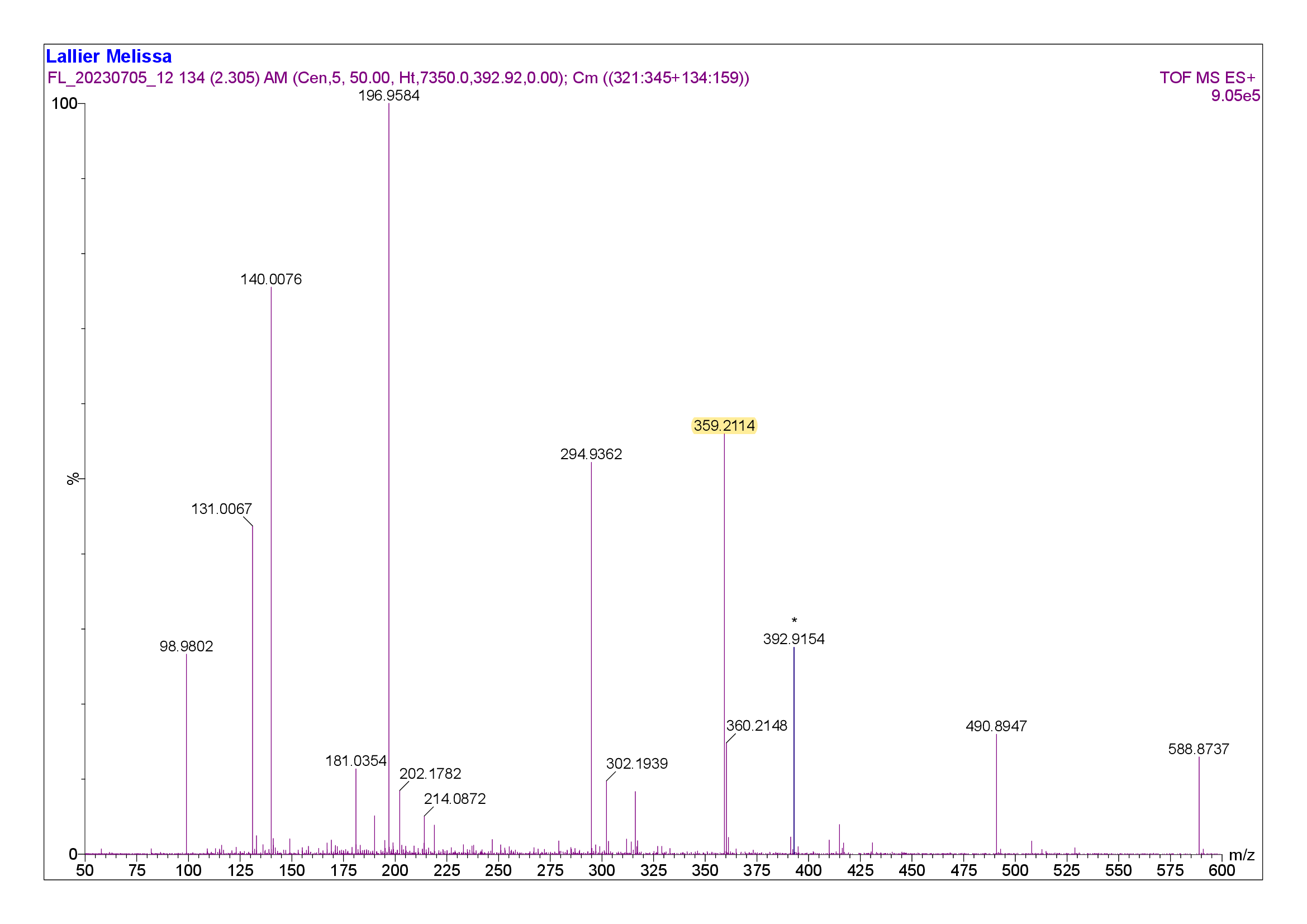
**


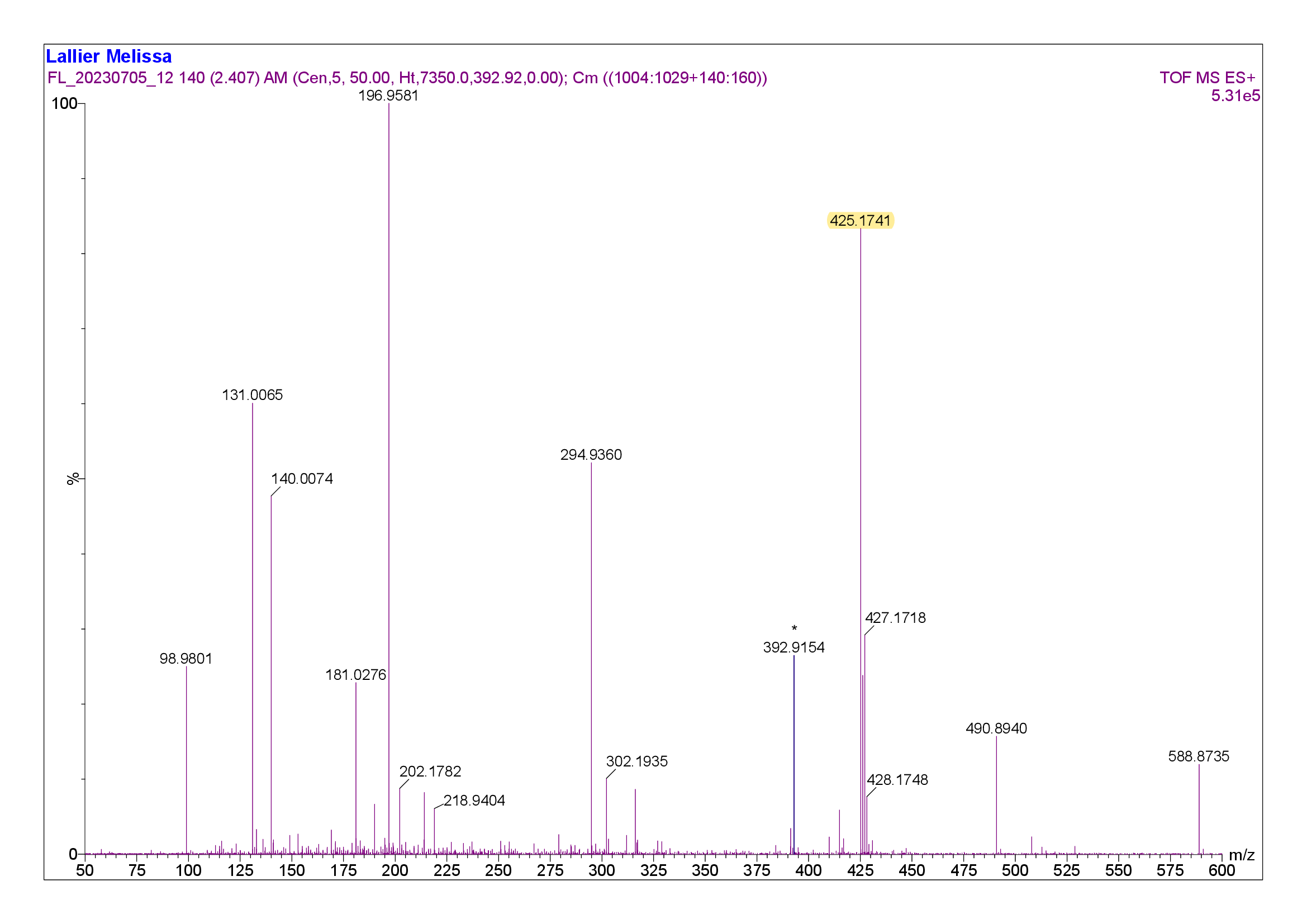
**Compound 2**

**Compound 5d**


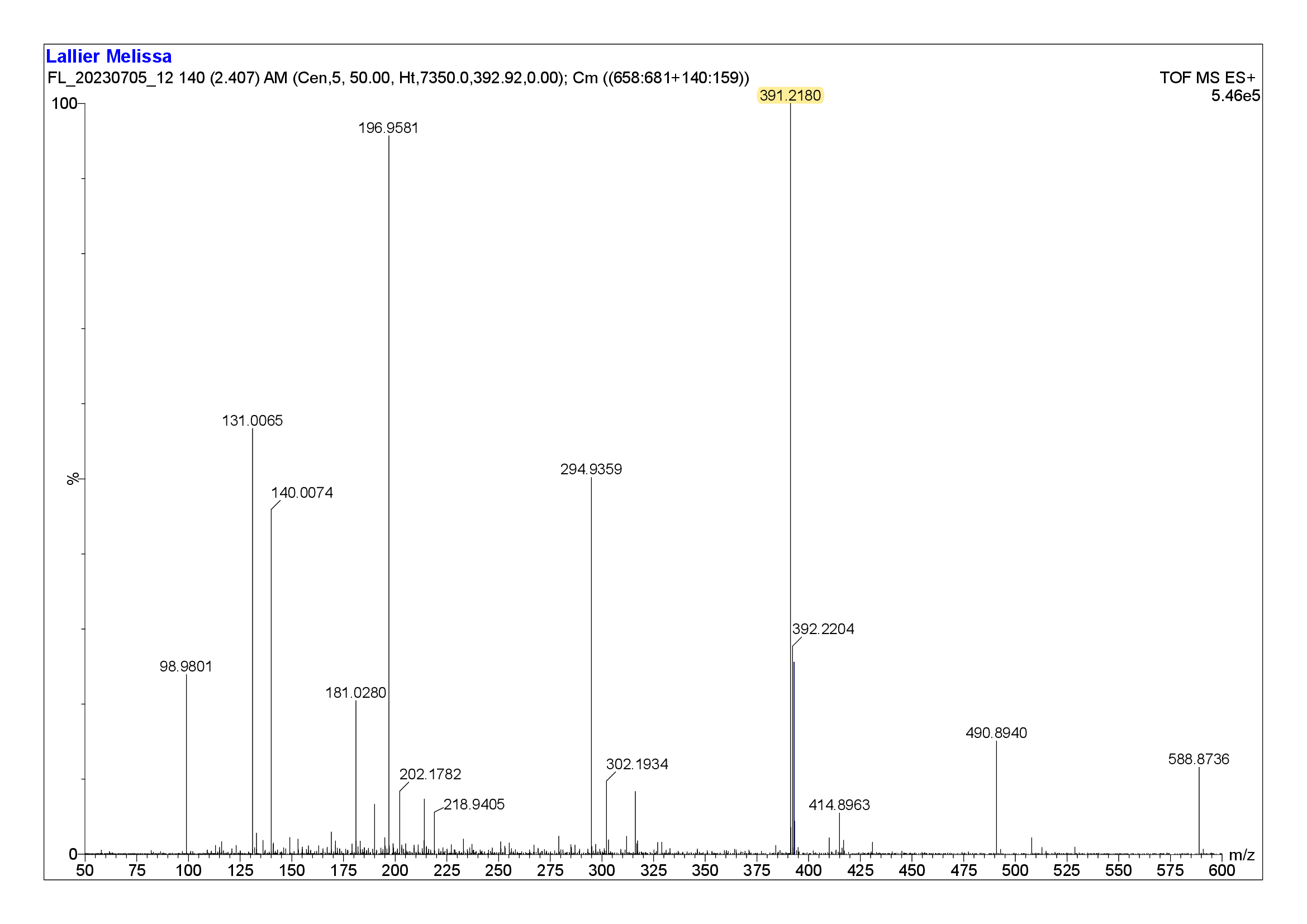


**Compound 5j**

**
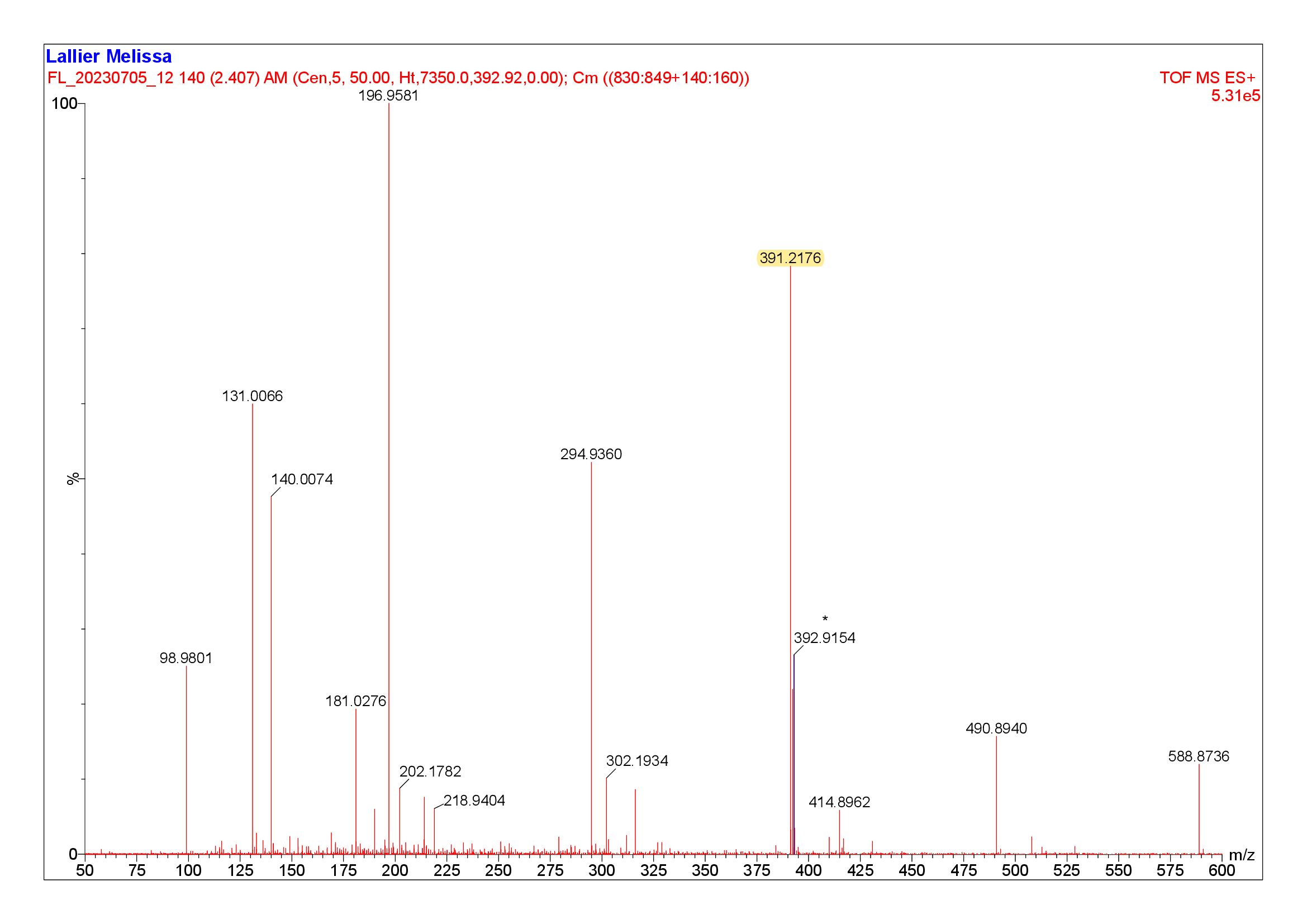
**

**NMR spectra of key molecules**

**^1^H NMR spectrum of compound 1**

**
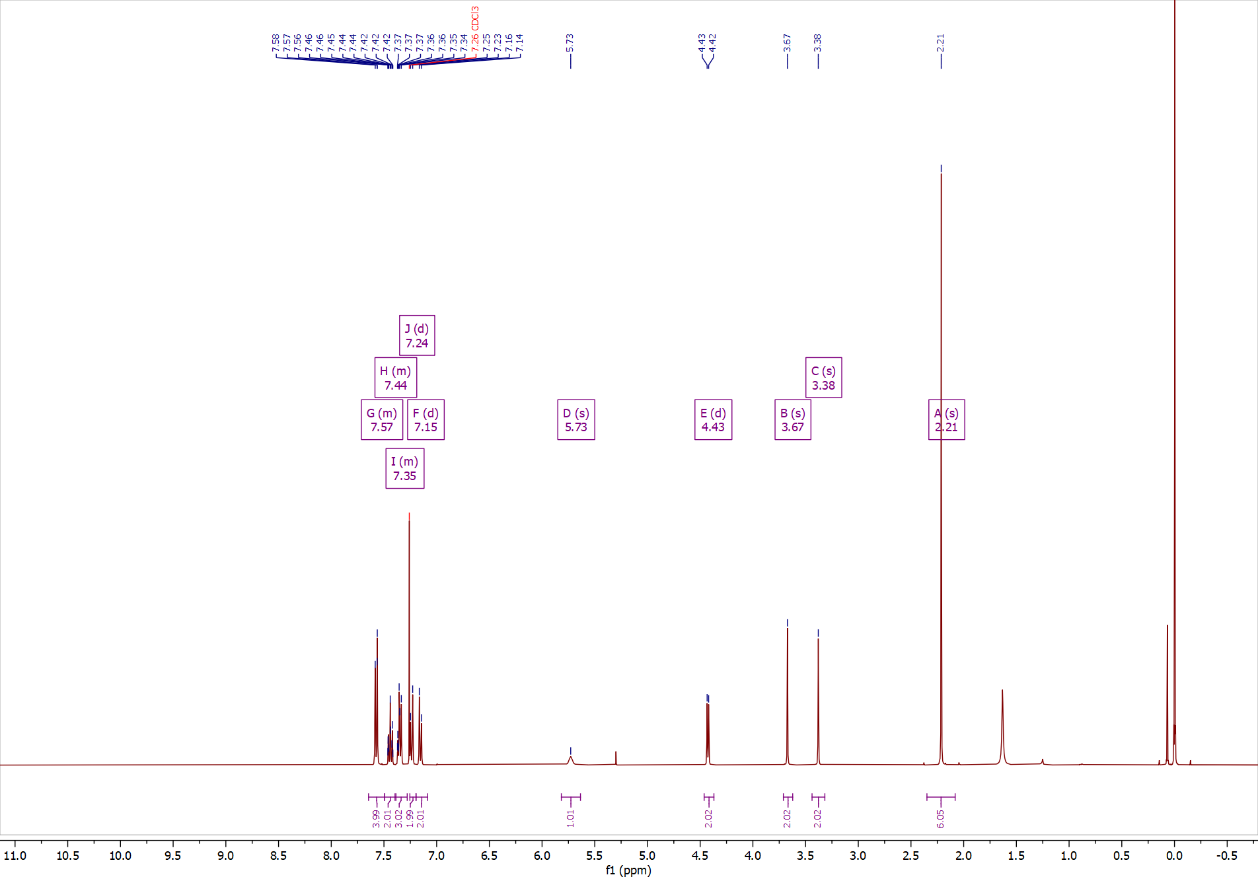
**

**^13^C NMR spectrum of compound 1
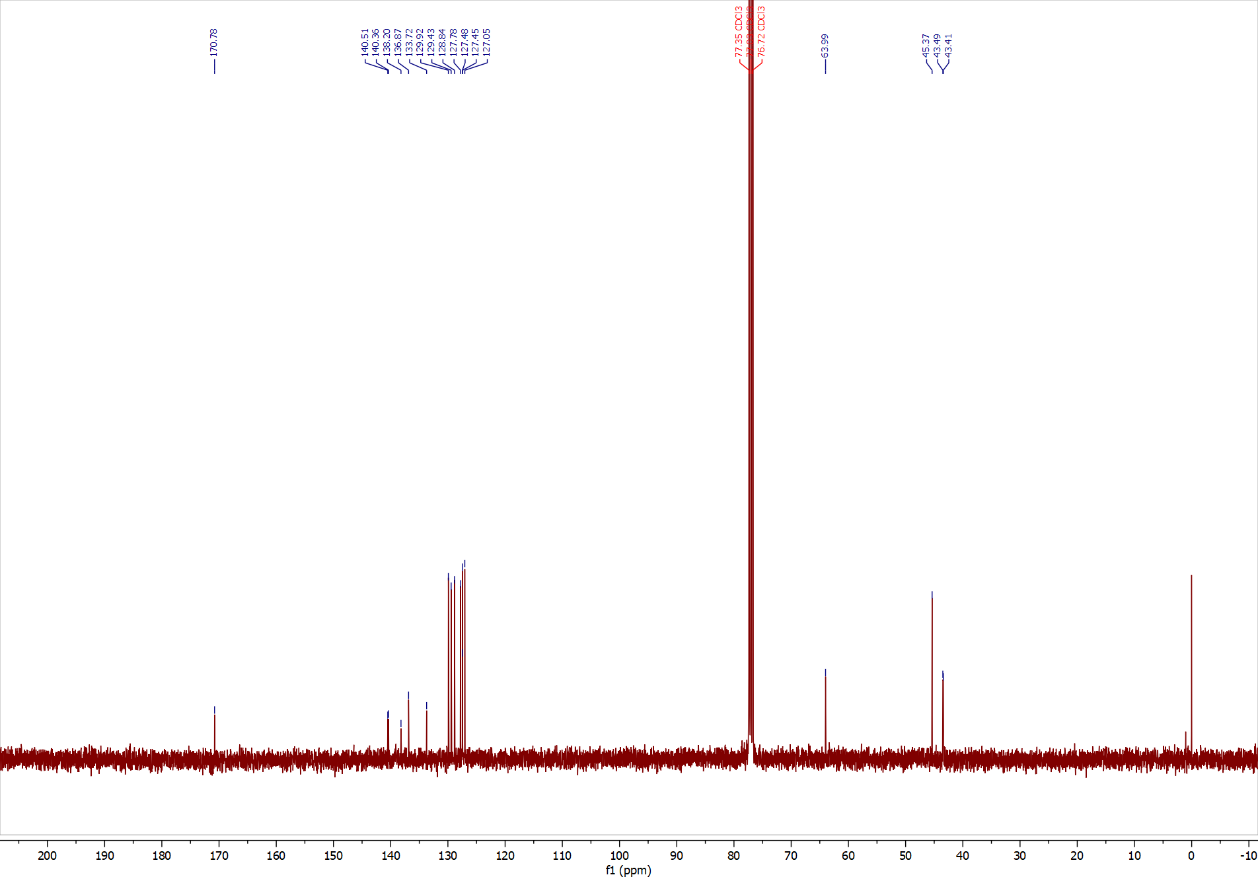
**

**^1^H NMR spectrum of compound 2**

**
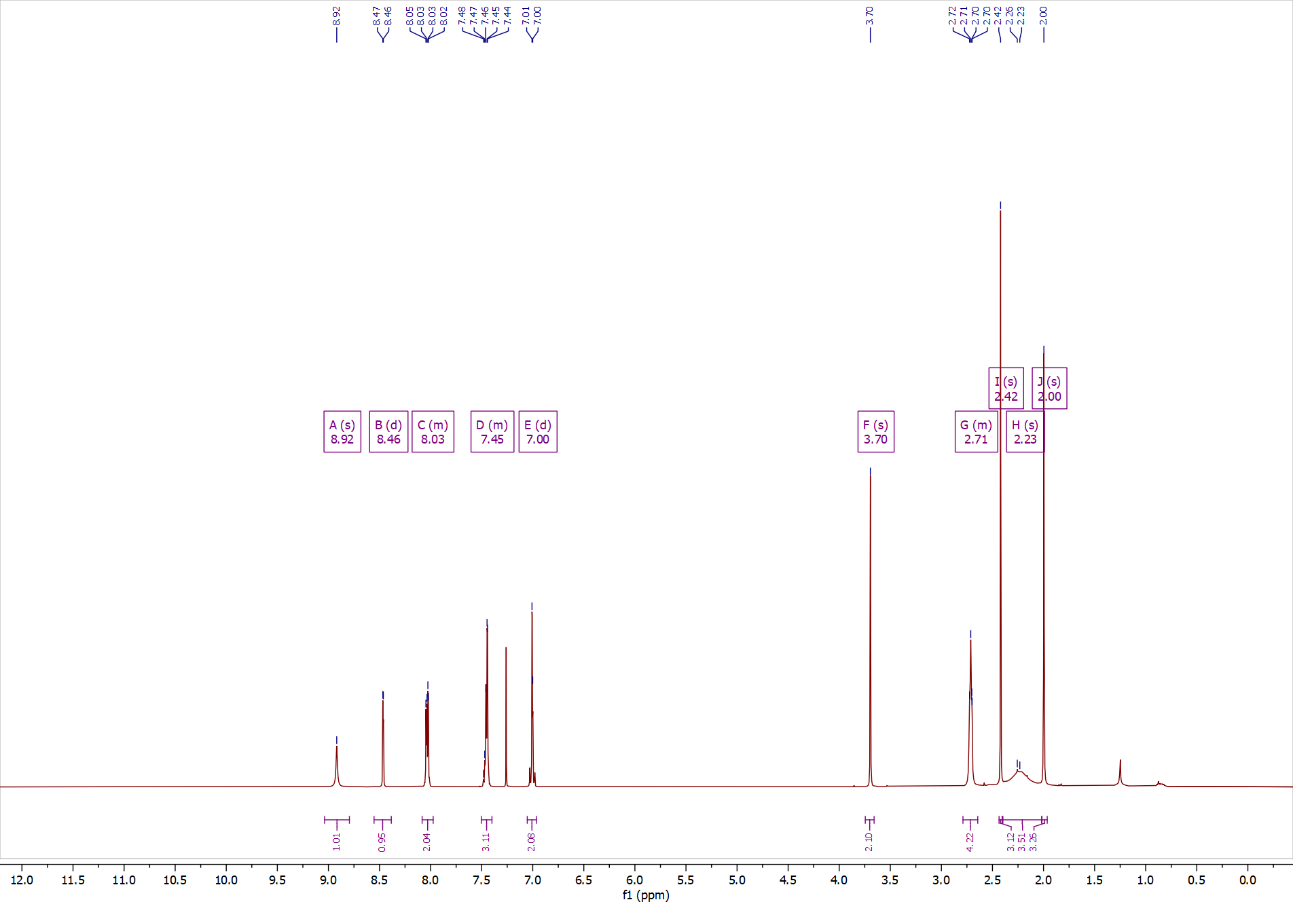
**

**^13^C NMR spectrum of compound 2**

**
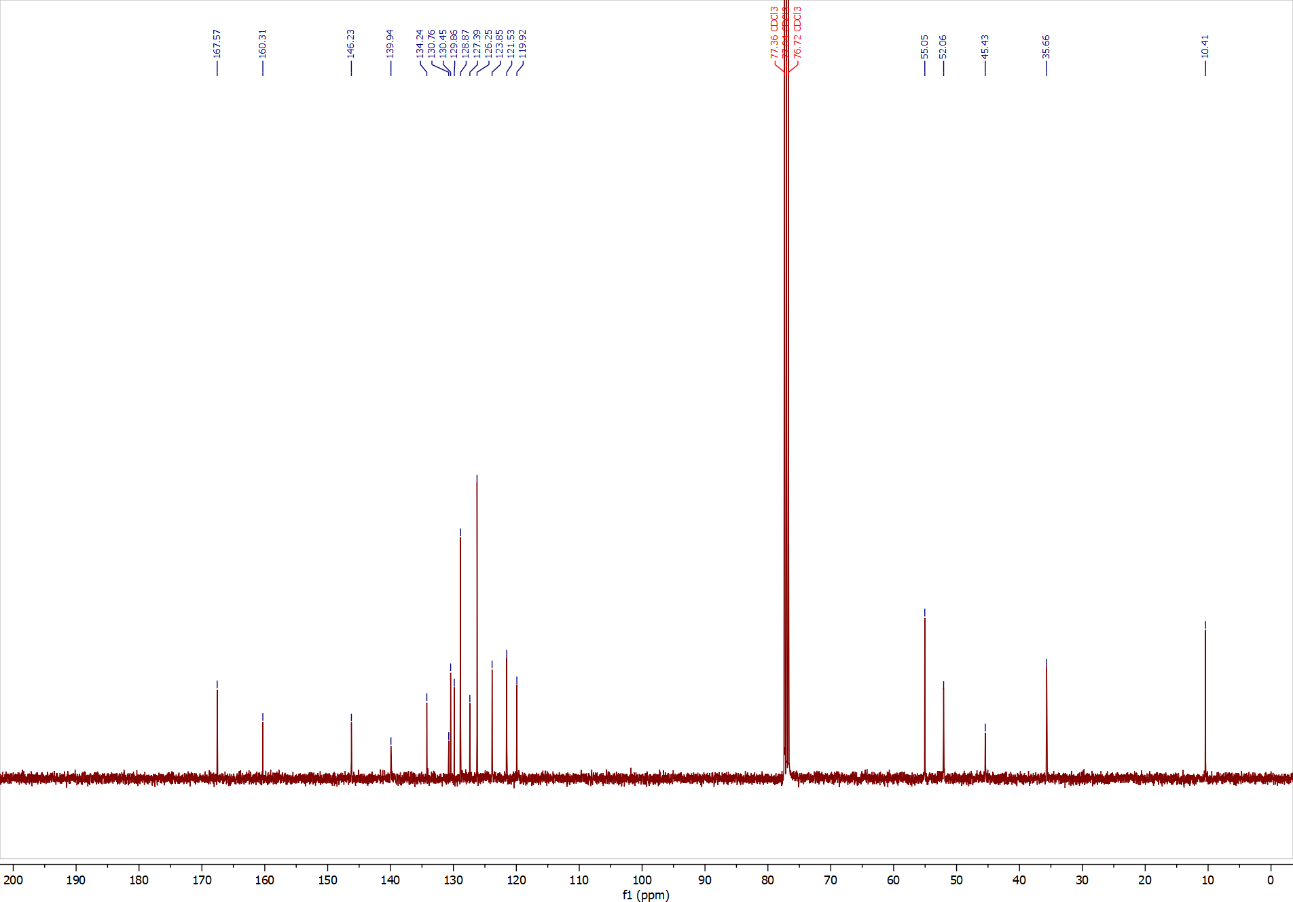
**

**^1^H NMR spectrum of compound 5d**

**
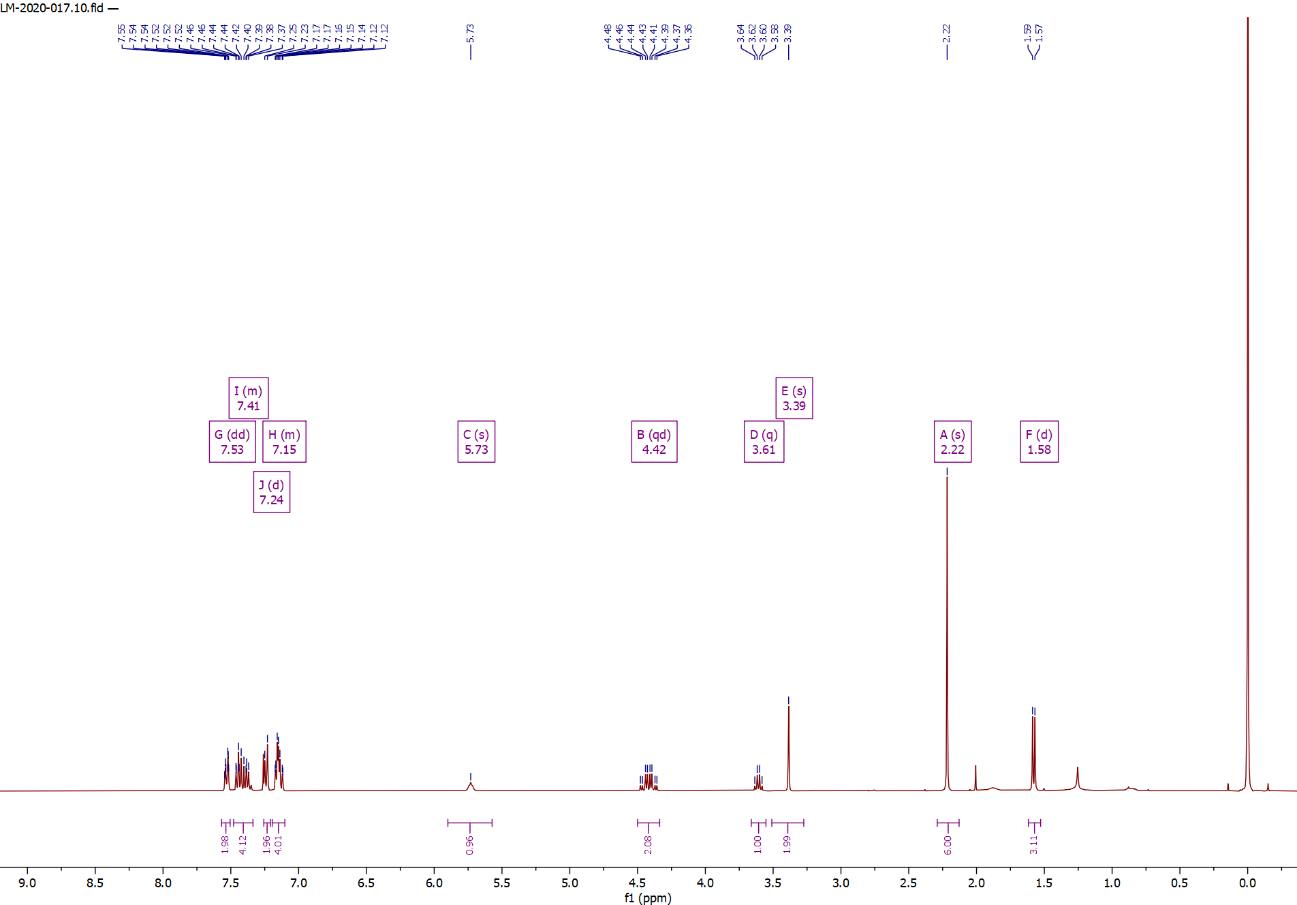
**

**^13^C NMR spectrum of compound 5d**

**
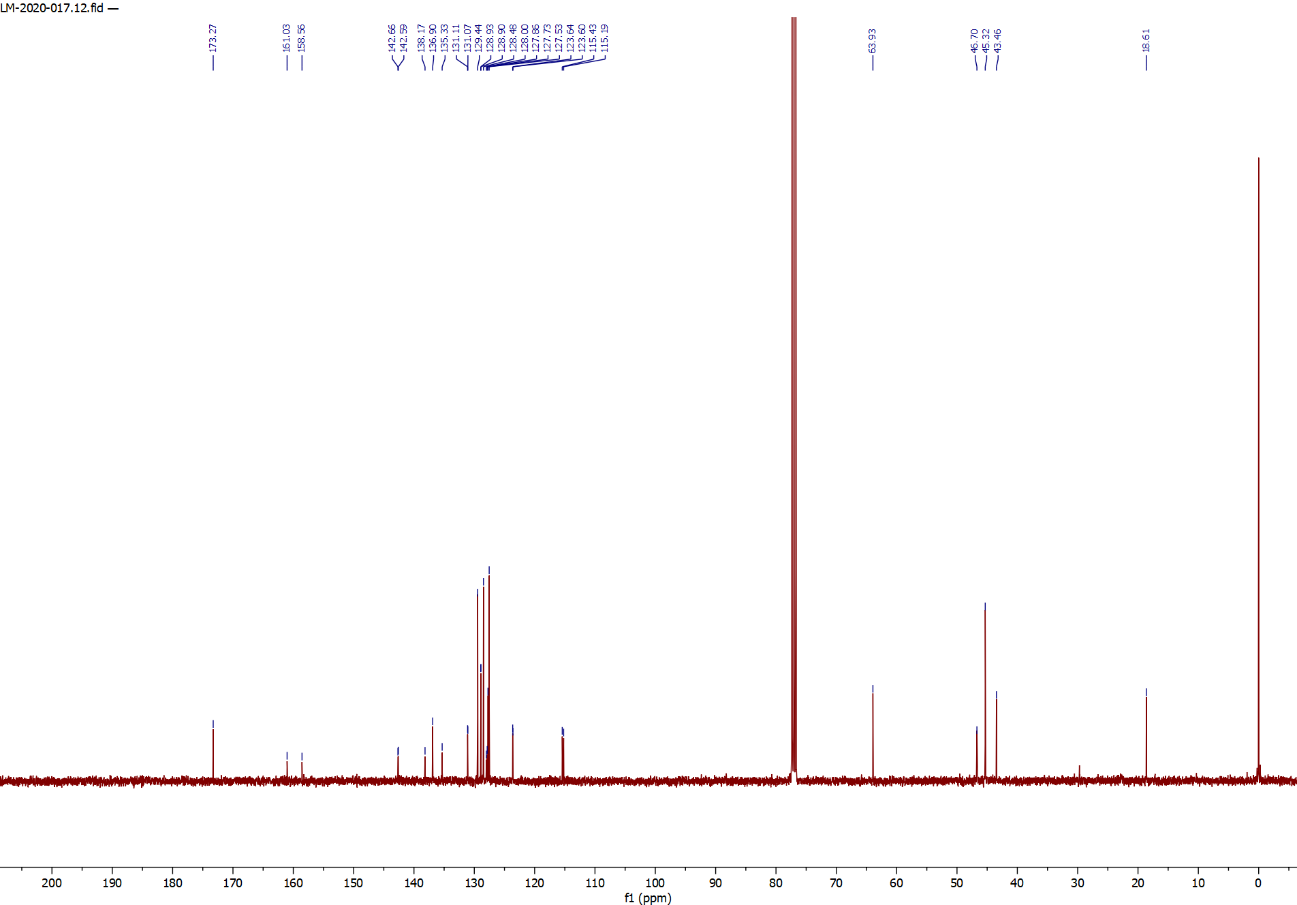
**

**^1^H NMR spectrum of compound 5j**

**
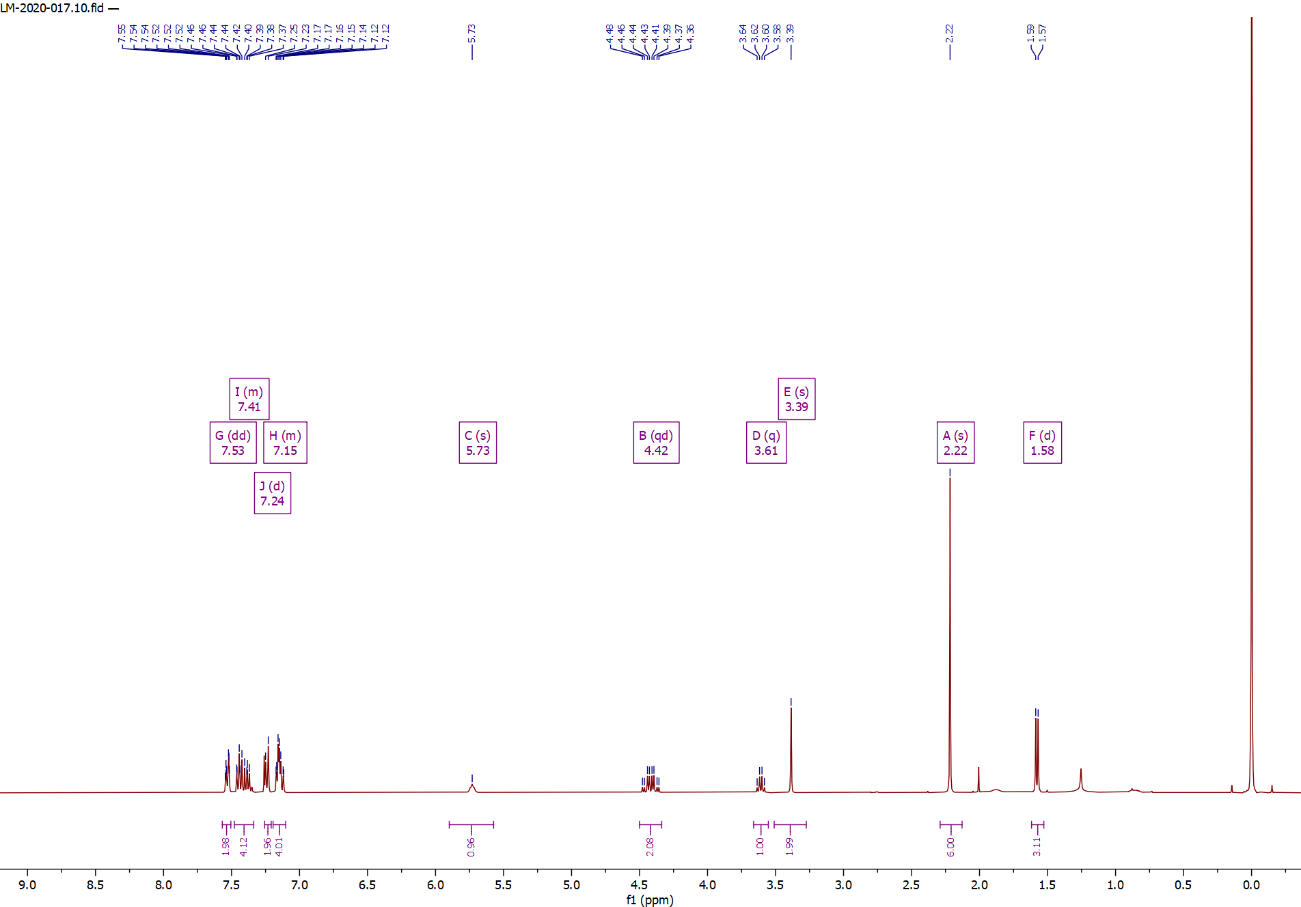
**

**^13^C NMR spectrum of compound 5j**

**
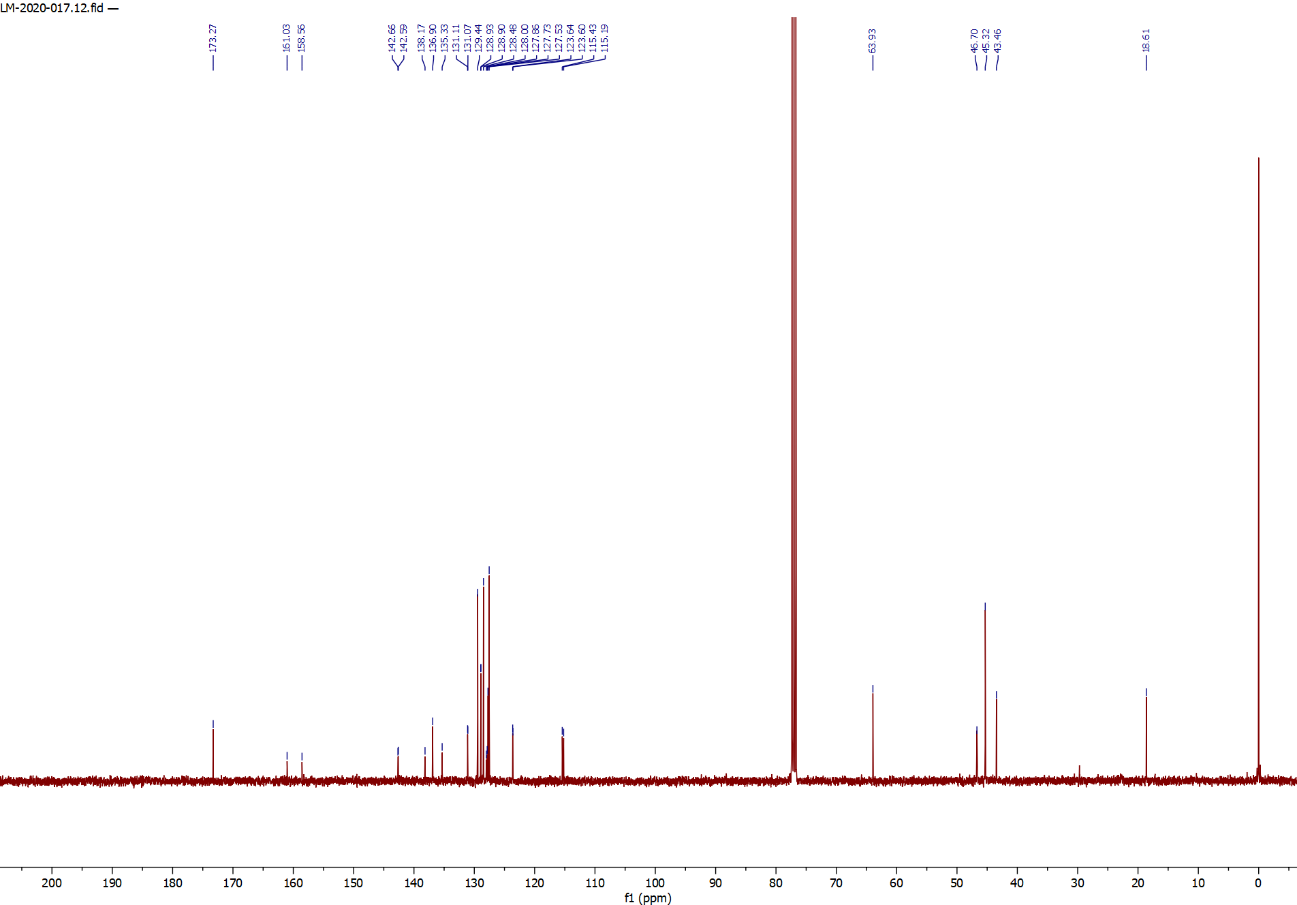
**
